# Using activated carbon produced from hazelnut shells as an adsorbent for the quantitative analysis of volatile organic compounds by GC-MS

**DOI:** 10.1007/s00216-026-06394-5

**Published:** 2026-02-18

**Authors:** Erdal Kusvuran, Ali Samil, Ender Gundogdu, Guray Kilincceker

**Affiliations:** 1 Chemistry Department, Arts and Sciences Faculty, Çukurovaukurova University, 01330 Adana, Turkey; 2 Chemistry Department, Sciences Faculty, Kahramanmaraş Sütcü İmam University, Kahramanmaraş, Turkey; 3 R&D Department, Alim Farm Ilac Kimya Medikal Ic Ve Dis Tic. Ltd., Sti, Ova Mah. 44160 Sk. No:30/5, 01100 Adana, Turkey

**Keywords:** Activated carbon, VOC, GC-MS, Adsorption, Agricultural waste

## Abstract

This study presents a sustainable approach utilizing activated carbon derived from hazelnut shells as an adsorbent for the simultaneous quantitative analysis of 48 different volatile organic compounds (VOCs) via GC-MS. The surface area of the produced activated carbon was successfully enhanced through the concurrent application of heat and steam. The effect of critical parameters, such as particle size and steam ratio, on the surface area and product yield was investigated. A high steam ratio was observed to increase the total surface area while decreasing the micropore surface area, which is attributed to the conversion of micropores into larger mesopores. Consequently, a 0.240–0.125 mm particle size and a 1.0 steam/AC ratio were selected, as they provided an optimal balance between back pressure and surface area. The method achieved exceptionally low limits of detection (LOD), with the lowest value of 3.72 µgm⁻^3^ obtained for chlorobenzene. The reliability of the method was confirmed by comparing its LOD values with those of the GC-MS instrument, revealing that LOD_Met_/LOD_GC-MS_ ratios were mostly between 1.0 and 2.0. The obtained LOD values were found to meet and often exceed the legal VOC limits of many countries, confirming the method's broad practical applicability.

**Graphical abstract:**

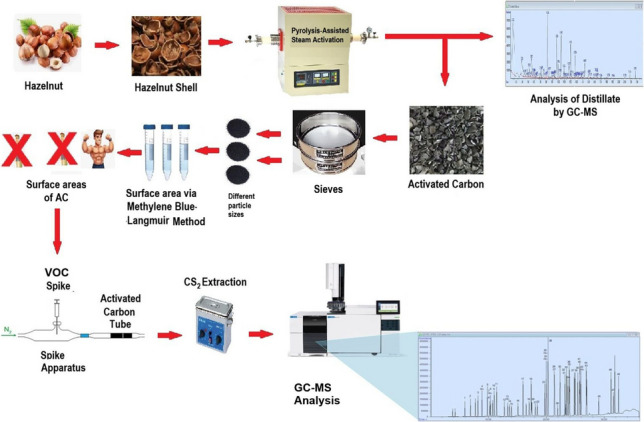

**Supplementary Information:**

The online version contains supplementary material available at 10.1007/s00216-026-06394-5.

## Introduction

Carbon has been a fundamental building block of human existence since the dawn of humanity. Beyond that, it is a material found in a wide range of applications, including industry [1, 2], health [3, 4], energy storage [5], the military defense industry [6], and digitalization [7]. In these sectors, carbon finds its use in forms such as coal, biochar, activated carbon (AC), and graphene. Among these high-carbon materials, AC is the most preferred carbon compound for use due to its superior qualities like its remarkably large surface area, chemical inertness, cost-effective production, and the ability to be produced in large quantities. The production of AC from various organic wastes, especially plant-based biomass, offers the dual advantages of environmental waste control and high-value waste utilization. Consequently, extensive research has been conducted on synthesizing AC from diverse plant-derived precursors [8–12]. In addition to its favorable lignocellulosic profile, hazelnut shell was selected as a cost-effective and abundant agricultural by-product, facilitating a sustainable approach to waste valorization in the production of high-performance adsorbents. This dual advantage of high performance and low cost makes hazelnut shells a superior precursor compared to other more expensive or less accessible biomass sources. The structural evolution of AC is fundamentally governed by the precursor’s lignocellulosic matrix and the activation chemistry employed. While wood-based materials such as sweet gum, cedar, and *Paulownia* provide extensive surface areas—reaching up to 2736 m^2^/g through ZnCl_2_ activation at a 4:1 impregnation ratio [8, 11, 12]—the choice of precursor remains a critical variable for specialized analytical applications. Lignin-rich precursors offer a distinct advantage; lignin typically comprises 35–40% of the hazelnut shell structure, which is significantly higher than the 20–25% found in standard hardwoods [9, 10]. This high lignin content ensures a more thermally stable and rigid carbon framework during carbonization. Furthermore, at optimized temperatures of 700–800 °C, the rapid release of volatiles facilitates the development of a tailored mesoporous architecture. This specific pore distribution is essential for the efficient adsorption and subsequent desorption of a diverse range of analytes. Through thermochemical conversion methods like pyrolysis and chemical activation, the surface area of AC has been reported to reach values as high as 2736 m^2^g⁻^1^ [12]. These ACs, with their remarkably large surface areas and numerous pores of varying sizes, are primarily utilized as powerful adsorbents for removing harmful chemicals from water and food and for eliminating industrial by-products.

In addition to these applications, studies are also being conducted on the removal of volatile organic compounds (VOCs), which are primarily emitted from various industrial sources. These sources include pesticide production and use [13, 14], adhesive manufacturing and application [15], pharmaceutical production [16], electronic component manufacturing [17], and the production of dyes [18], solvents, synthetic resins [19], and polymers [20], as well as numerous other industrial processes. VOCs are a large group of carbon-based chemicals that readily evaporate at room temperature. VOCs are classified as major contributors to air pollution, with many of these compounds having diverse environmental and toxicological effects. They act as both indirect contributors to environmental toxicity [21]. VOCs can be categorized as halogenated compounds (both aromatic and non-aromatic), aldehydes, aromatic hydrocarbons, and polycyclic aromatic hydrocarbons [22]. Alonso et al. [23] reported that halogenated VOCs exhibit strong bioaccumulation, acute toxicity, and refractory structures, making them harmful compounds. Because these compounds are highly volatile and often have long atmospheric lifetimes, humans can be exposed through various routes, including drinking water and inhalation [24, 25]. The second group of VOCs is comprised of aldehydes; even low-dose exposure can lead to symptoms such as throat irritation, shortness of breath, eye irritation, and chest tightness [26]. Elevated exposure to aldehydes increases the risk of acute poisoning, while chronic exposure may result in detrimental effects like chronic toxicity in humans [26, 27]. Aromatic hydrocarbons constitute another category of VOCs. Even at low concentrations, they can cause numerous adverse effects on human health, including weakness, confusion, nausea, loss of appetite and memory, tiredness, and vision impairment. Moreover, high concentrations can lead to unconsciousness, dizziness, or even death [16, 26]. Polycyclic aromatic hydrocarbons (PAHs), known carcinogens [28], constitute the last group of VOCs. They cause pulmonary and cardiovascular diseases, immune system impairment, and adverse birth outcomes [29]. Additionally, exposure, whether prenatal or in adulthood, can result in cardiovascular disease [29] and contribute to adverse birth outcomes like preterm birth [30, 31] and neural tube defects during gestation [31]. Some VOCs, particularly chlorinated ones, exhibit greater toxicity and high chemical stability. Because they are considered hazardous air pollutants, many countries classify them as harmful emissions [32]. Because current legislation restricts VOC emissions, effective emission reduction technologies are necessary. To achieve this goal, various techniques have been investigated by many researchers. These include adsorption techniques such as AC [33], zeolites [34], and polymeric adsorbents [35]; low-temperature condensation [36, 37]; membrane separation [38]; and oxidation techniques like thermal [39], catalytic [14, 40], UV/catalytic [41], or bacteriological methods [42]. Beyond the removal and reduction of VOCs, their quantitative analysis is also crucial, as accurate determination of VOC concentration is essential for the success of removal and reduction processes. Most researchers have focused on removing VOCs from the atmosphere and reducing emissions from their sources to create a healthier environment [14, 40]. While these studies often investigate a limited number of VOCs, some have specifically focused on their quantitative analysis [37, 43–45]. To enhance the accuracy of VOC analysis, several instruments are used, including gas chromatography (GC), gas chromatography-mass spectrometry (GC-MS) [45, 46], proton transfer reaction mass spectrometry (PTR-MS)/proton transfer reaction time-of-flight mass spectrometry (PTR-ToF-MS) [47], and selected ion flow tube-mass spectrometry (SIFT-MS) [43]. Even micro-sensors have been employed to determine the concentration of specific VOCs like acetone, hexanal, and 2-pentanone [48].

In this study, high surface area AC was first produced from hazelnut shells. Using this AC, a simultaneous quantitative analysis of 48 different VOCs was performed. These VOCs included aliphatic and aromatic compounds, as well as their mono-, di-, and tri-substituted chlorine, bromine, and methyl derivatives. Various validation parameters of the applied method were determined, such as the limit of detection (LOD), linearity, repeatability, reproducibility, and accuracy. The LOD was calculated and comparatively discussed using three different approaches: the LOD of the instrument used, the LOD obtained from the calibration curve, and the LOD of the applied.

## Materials and method

### Materials

The hazelnut shells used for the synthesis of AC were obtained from hazelnut producers in Giresun, Turkey. An Isotherm brand furnace, operating between 200 and 1200 °C with a temperature deviation of ± 10 °C, was used for the carbonization process. A Shimadzu UV-2101PC UV-VIS double-beam scanning spectrophotometer was used at a wavelength of 660 nm to determine the equilibrium concentration of methylene blue, which was used to find the surface area of the AC. A PerkinElmer Spectrum 100 FTIR instrument was utilized to monitor the process of converting hazelnut shells into AC. The Brunauer, Emmett, and Teller (BET) surface area of the produced AC was measured using a Micromeritics, TriStar II Plus 2.00 instrument. The surface morphology of the AC was monitored by Field Electron and Ion Company Quanta FEG650 scanning electron microscopy (SEM). All chemicals used in this study were obtained from Merck. Both VOC and distillate analyses were performed using an Agilent 7890A gas chromatograph coupled with an Agilent 5975C Triple-Axis mass selective detector (MSD) and equipped with an Agilent 7693A Autosampler. Data acquisition and processing were carried out using the Agilent ChemStation software. Probable compounds were identified using the National Institute of Standards and Technology Mass Spectral Library (NIST 11) by comparing the obtained product patterns with those in the reference database. For the VOC analysis, a Restek Rtx-Volatiles GC capillary column (30 m × 0.25 mm, 1.0 µm film thickness) was used, while a J&W DB-624 capillary column (60 m × 0.32 mm, 1.8 µm film thickness) was used for the distillate analysis.

### Methods

#### Production of AC from hazelnut shell

First, all of the obtained hazelnut shells were ground to a particle size between 4.00 and 2.00 mm. One hundred grams of these ground hazelnut shells was weighed and placed into a reactor before being positioned in a furnace, where they were simply exposed to heat under nitrogen gas for 1 h at a temperature of 200 °C. After carbonization, the reactor was cooled to room temperature using tap water. The contents of the reactor were weighed again and then ground using a laboratory grinder (Sinbo SCM 2934). The ground material was then separated into three different particle size ranges using sieves: 0.075–0.125 mm, 0.125–0.240 mm, and 0.240–0.420 mm, and stored for further analysis. The same procedure was applied at 300 °C, 400 °C, 500 °C, 600 °C, 700 °C, 800 °C, and 900 °C, respectively.

#### Steam activation for enhanced surface area

To investigate the effect of steam on improving the AC surface, steam was sent to the hot AC during pyrolysis. For this purpose, 100 g of the hazelnut shells, also ground to a particle size between 4.00 and 2.00 mm, was placed in a reactor and then positioned in the furnace. The amount of water corresponding to the pre-determined steam/AC ratios was weighed and vaporized in a pre-heating zone prior to its introduction into the furnace. Following this, steam was passed through the reactor from bottom to top for 1 h at the designated furnace temperature while nitrogen gas was passed from the furnace to purge oxygen. Specifically, the steam, carried by nitrogen (N₂) carrier gas, was introduced at a controlled rate from the bottom of the reactor through a fishbone-shaped pipe manifold featuring small perforations. This design ensured an even distribution of the steam throughout the carbon bed for uniform activation. However, this procedure was applied only at 900 °C because the highest surface area of AC was obtained at this temperature during the previous pyrolysis study. After it was cooled, the material was similarly separated into three particle size ranges (0.075–0.125 mm, 0.125–0.240 mm, and 0.240–0.420 mm) and stored. Thereafter, the surface areas were determined using methylene blue (MB) and the Langmuir equation.

#### Material characterization

The changes in the functional groups of the hazelnut shell as a function of temperature were observed using FTIR spectroscopy. For this analysis, previously produced ACs with a particle size range of 0.125–0.075 mm for each temperature were kept in an oven at 105 °C for 3 h, cooled in a desiccator, and then prepared into pellets with potassium bromide for FTIR analysis.

To identify the thermal cracking products formed during the carbonization process, 100 g of the sample was loaded into a reactor. A condenser was connected to the reactor outlet to collect the resulting pyrolysis distillate while the furnace temperature was reaching 500 °C. The collected distillate was subsequently analyzed using a GC-MS instrument.

SEM analyses were performed to investigate in detail the surface morphology, pore structure, and topographical properties of the AC. The AC with a particle size range of 0.125–0.075 mm, produced at 900 °C in the presence of steam, was selected for this process due to its superior surface area.

#### Determination of adsorbent surface area

The surface area of the produced AC was determined by MB adsorption, with the equilibrium data fitted to Langmuir equation (Eq. 1). According to Eq. 1, When the values of ${\mathrm{C}}_{\mathrm{e}}/{\mathrm{q}}_{\mathrm{e}}$ are plotted against ${\mathrm{C}}_{\mathrm{e}}$, the inverse of the slope of the resulting line gives the maximum adsorption capacity $({Q}_{\mathrm{max}})$.

1 $$\frac{{\mathrm{C}}_{\mathrm{e}}}{{\mathrm{q}}_{\mathrm{e}}}\text{ = }\frac{1}{{{Q}_{\mathrm{max}}K}_{\mathrm{L}}} \, \mathrm{+}\frac{1}{{Q}_{\mathrm{max}}}{\mathrm{C}}_{\mathrm{e}}$$

Here, ${\mathrm{C}}_{\mathrm{e}}$ is the Equilibrium concentration of the MB (mg/L); ${\mathrm{q}}_{\mathrm{e}}$ is the equilibrium adsorption capacity (mg/g); ${K}_{L}$ is the Langmuir constant (L/mg), which is related to the affinity of the binding sites on the adsorbent; and ${Q}_{\mathrm{max}}$ is the maximum adsorption capacity (mg/g).

Once ${Q}_{\mathrm{max}}$ is obtained from the Langmuir equation, the surface area of the AC can be easily calculated as shown below (Eq. 2).

2 $$SA=\frac{{Q}_{max}.{N}_{A}.A}{{M}_{W}}$$

where $SA$ is the surface area of AC (m^2^/g); ${N}_{A}$ is the Avogadro’s number (6.022.10^23^); $A$ is the MB cross-sectional area (130 Å^2^); and ${M}_{W}$ is the molecular mass of MB (355.89 g/mol).

To carry out the surface area of AC, six concentrations were prepared: 100, 200, 300, 400, 500, and 600 mg/L. For three replicate experiments, 25 mg of AC was weighed into 15-mL Falcon tubes with screw caps. Ten milliliters of 100 mg/L MB solution was added to each. The same procedure was applied for 200, 300, 400, 500, and 600 mg/L MB solutions. After the caps were closed, the tubes, including the blank tubes containing only MB solution, were shaken on a shaker for 8 h at 25 ± 1 °C and 150 rpm. Subsequently, a 2-mL sample was withdrawn from each tube using an injector and filtered (0.1 µm pore size) into a vial for the UV/Vis instrument and measured at a 660-nm wavelength. For each of the three different particle size ranges (0.075–0.125 mm, 0.125–0.240 mm, and 0.240–0.420 mm), the same procedure was repeated.

BET surface area measurements were conducted on a few ACs with the highest surface areas, which were determined as a result of MB and Langmuir measurements. In order to compare them on the same metric, BET surface area measurements were conducted on a few ACs that had the highest surface areas determined by the MB and Langmuir measurements. There are significant differences between the surface areas of MB molecules and N_2_ molecules, which is why the measurement results are different; BET typically yields higher results.

Even though the 0.075–0.125 mm particle size fraction produced with steam exhibited the highest surface area, the 0.125–0.240 mm particle size fraction was chosen for further VOC analysis because it created less back pressure during gas flow. 0.4000 g of these samples was weighed, placed in silica tubes, and the tubes were sealed at both ends using a flame for storage until VOC analysis.

### Quantitative analysis of VOC

To determine the VOC concentrations in this study, VOC-Mix 20 (Lot Number: 30412ME), purchased from Dr. Ehrenstorfer GmbH, was used as the standard without further purification. For the quantitative analyses of VOCs, solutions of each VOC were prepared at concentrations ranging from 1250.00 µg/L to 19.53 µg/L in methanol. Each standard solution was injected in triplicate using GC-MS. For a typical VOC analysis, the injection port and interface temperatures were maintained at 250 °C, while the ion source temperature was set to 230 °C. The injection was performed in splitless mode for 0.5 min using helium as the carrier gas at a constant flow rate of 1.5 mL/min. The oven temperature program was initiated at 40 °C (held for 3 min), increased to 90 °C at a rate of 8 °C/min (held for 4 min), and finally raised to 200 °C at 6 °C/min, with a final hold time of 4.5 min.

Six compounds (1,2-dichloroethane-d_4_ (> 99.0%), trichloromethane-d_1_ (≥ 99.9%), 1,2-dichloropropane-d_6_ (98.0%), 1,1,1,2-tetrachloroethane-d_2_ (≥ 98.5%), ethylbenzene-d_10_ (> 99.0%), and 1,4-dichlorobenzene-d_4_ (98.0%)) were purchased from Sigma for using internal standards. 1,2-Dichloroethane-d_4_ abbreviated as *IS*_*1*_ was used as internal standards for the quantification of 1,1-dichloroethene (99.7%), trans-1,2-dichloroethene (99.6%), cis-1,2-dichloroethene (98.9%), 1,2-dichloroethane (99.7%), and 1,2-dibromoethane (99.7%). Trichloromethane-d_1_, similarly abbreviated as *IS*_*2*_, was used for the quantification of dibromomethane (99.5%), bromochloromethane (98.6%), trichloromethane (99.8%), tribromomethane (99.0%), dibromochloromethane (99.2%), and bromodichloromethane (97.4%). 1,2-Dichloropropane-d_6_, *IS*_*3*_, was used in the quantification of the group of compounds such as 1,2-dichloropropane (99.8%), 1,3-dichloropropene (cis (97.5%) and trans (98.4%) isomers), 1,3-dichloropropane (98.3%), 1,1-dichloro-1-propene (95.6%), 1,2-dibromo-3-chloropropane (98.1%), and hexachloro-1,3-butadiene (96.6%). The concentrations of the polychlorinated compounds trichloroethene (99.7%), 1,1,1-trichloroethane (97.1%), 1,1,2-trichloroethane (99.6%), 1,1,1,2-tetrachloroethane (99.3%), tetrachloroethene (99.9%), and 1,1,2,2-tetrachloroethane (98.6%) were determined using 1,1,1,2-tetrachloroethane-d_2_ (*IS*_*4*_) as an internal standard. On the other hand, the concentrations of alkyl-substituted aromatic rings, specifically benzene (99.9%), toluene (99.8%), p-xylene (99.7%), m-xylene (99.7%), o-xylene (99.0%), styrene (99.9%), ethylbenzene (99.6%), isopropylbenzene (98.5), n-propylbenzene (99.9%), 1,3,5-trimethylbenzene (99.7%), tert-butylbenzene (99.5%), 1,2,4-trimethylbenzene (99.7%), sec-butylbenzene (99.9%), 4-isopropyltoluene (97.7%), and n-butylbenzene (98.6%), were determined via ethylbenzene-d_10_ (*IS*_*5*_) internal standard. Finally, the concentrations of the halogenated aromatic rings, including chlorobenzene (99.9%), bromobenzene (99.5%), 1,3-dichlorobenzene (99.1%), 1,4-dichlorobenzene (99.9%), 1,2-dichlorobenzene (99.9%), 1,2,4-trichlorobenzene (99.8%), 1,2,3-trichlorobenzene (99.9%), 4-chlorotoluene (99.9%), and 2-chlorotoluene (99.9), were determined using 1,4-dichlorobenzene-d_4_ (*IS*_*6*_) as an internal standard. Following the adsorption of VOCs onto AC, desorption was carried out using carbon disulfide (CS_2_, Merck, Germany) as the desorption solvent. A Nüve NF 800R (Ankara, Turkey) centrifuge and a Kudos SK-5210HP ultrasonic bath were employed in the desorption procedure.

A commercial activated carbon (CAC) (TCRTECCORA brand, Corsico, Italy, Lot No.: 0870) was used to compare its VOC adsorption/desorption properties with the AC synthesized in this research. The evaluation was carried out at VOC concentrations of 150 and 2400 mg/L.

#### Analytical method performance parameters

In this section, performance parameters such as the LOD, linearity, repeatability, and reproducibility were determined. Primarily, the limit of detection of the GC-MS instrument (LOD_GC-MS_) was determined using standard VOC solutions. For this, 48 VOC compounds, each at a concentration of 100 µg/L, were injected into the GC-MS. The signal-to-noise ratios (S/N) were then determined for each peak; compound peaks with an S/N of less than 6 were not accepted as signal peaks. Each VOC concentration was normalized to achieve an S/N of 6, and these concentrations were set as the instrument’s LOD. The normalization process was carried out with the simple equation shown below (Eq. 3).

3 $${C}_{\mathrm{norm}}={C}_{\mathrm{inj}}\frac{({S/N)}_{\mathrm{thresh}}}{(S/N{)}_{100}}$$

where ${C}_{\mathrm{norm}}$ is the lowest concentration of VOC corresponding to the S/N 6 value;

${C}_{\mathrm{inj}}$ is the concentration of VOC injected GC-MS, µg/L; 

$(S/N{)}_{100}$ is the S/N value of VOC at 100 µg/L; and 

$({S/N)}_{\mathrm{thresh}}$ is the S/N (6) value accepted as a peak.

Two different LOD approaches were used, with the first approach accepting the normalized VOC concentrations as their GC-MS detection limits (LOD_GC-MS_); this included the evaluation of 1,2-dibromo-3-chloropropane (with the lowest S/N ratio) and chlorobenzene (with the highest S/N ratio) at 100 µg/L. The other approach determines the LOD based on the relationship between the mean and standard deviation of recovery values from spiked samples, which were prepared at a concentration slightly above a signal-to-noise (S/N) ratio of 6, as shown below (Eq. 4).

4 $${\mathrm{LOD}}_{\mathrm{Met}}=C+3\sigma$$

where $C$ is the VOC concentration slightly above S/N 6, and $\sigma$ is the standard deviation of the recovery values for the VOC concentration. The abbreviation LOD_Met_ was used to denote the LOD of the applied method. To determine the LOD_Met_ levels for each VOC, spikes were performed at concentrations slightly above their respective LOD_GC-MS_ levels. The sampling apparatus used for this procedure can be seen in Supplementary Materials 1 (S.1). The mean values of the ten individual recovery studies resulting from the spike studies were calculated and determined as the LOD_Met_ of the applied method. For the linearity study of the applied method, six concentrations ranging from 75 to 2400 µg/L were selected, and spiking was performed.

#### Adsorption and desorption procedure

The adsorption and desorption conditions were optimized based on modified NIOSH 1501 [49] and EPA TO-17 [50] protocols to accommodate the characteristics of the hazelnut-shell-derived AC. For the recovery experiments, a custom-made silica apparatus was designed to ensure controlled and uniform loading of the target VOCs. The silica tubes containing 0.4000 g of AC were opened at both ends and integrated into the sampling system using short silicone connectors. Target VOC concentrations (spikes) were then injected into the apparatus via a silicone inlet using a syringe. High-purity nitrogen (N_2_, 99.999%) at a constant flow rate of 0.5 L/min was maintained for 30 min to facilitate the complete transfer of the analytes into the AC bed. This setup ensured optimal interaction between the VOCs and the adsorbent while preventing breakthrough throughout the loading process.

Following the adsorption, the silica tube was opened, and the contents were transferred to 15-mL polypropylene tubes for the desorption stage. Carbon disulfide (CS_2_) was selected as the desorption solvent due to its high efficiency for the target analytes. A volume of 4 mL CS_2_ was added, and the mixture was subjected to ultrasonic treatment for 10 min. Throughout the extraction and subsequent 5-min centrifugation, a temperature of +2 °C was strictly maintained to prevent the evaporation of highly volatile compounds. The resulting supernatant (1.5 mL) was transferred to screw-capped vials for GC-MS analysis. The same procedure was repeated for the blank. Spike and blank studies were both replicated 10 times.

To determine whether the AC was compatible with the applied VOC analysis method, repeatability and reproducibility studies were conducted. For this purpose, repeatability experiments were performed by three different researchers at two VOC concentrations (100 and 1000 µg/L), with five replicates for each. For reproducibility, these same three researchers conducted experiments on five different days at the same concentrations.

### GC-MS analysis of VOCs

The analyses of VOCs were carried out using GC-MS as described previously. GC oven was held at 40 °C for 3 min, then raised by 8 °C/min to 90 °C and held on this temperature for 4 min. Then, the oven temperature was raised 6 °C/min to 200 °C and maintained for 4.5 min at 200 °C. The temperature of the injection port was set at 250 °C while the interface and ion source temperatures were 250 and 230 °C, respectively. The carrier gas, He, flowrate was adjusted to 1.5 mLmin^−1^. The quantification of VOCs was performed in the electron ionization (EI) and selected ion monitoring (SIM) mode. Before SIM mode was applied, each VOC (1 µL, 1000 µg/L) was injected into GC-MS and the product ion (Q_1_) of each VOC for quantitation was determined. The Q_1_ ions were used as the quantification ion in SIM mode. The other ions (Q_2_, Q_3_, and Q_4_) of each VOC were used as confirmation ions. For the quantitative analyses of VOCs, a standard solution of each VOC was prepared at a concentration range similar to the method mentioned above in *methanol *and analyzed three times using GC-MS.

## Results and discussion

### Hazelnut shell carbonization and product yield

**Fig. 1:**
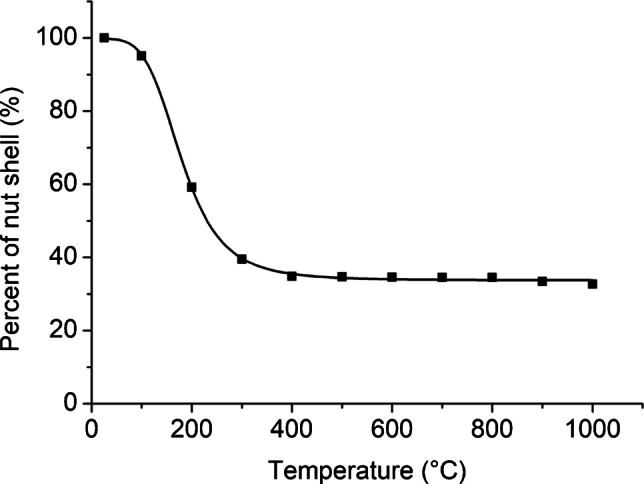
Temperature-dependent mass loss of hazelnut shell

**Fig. 2:**
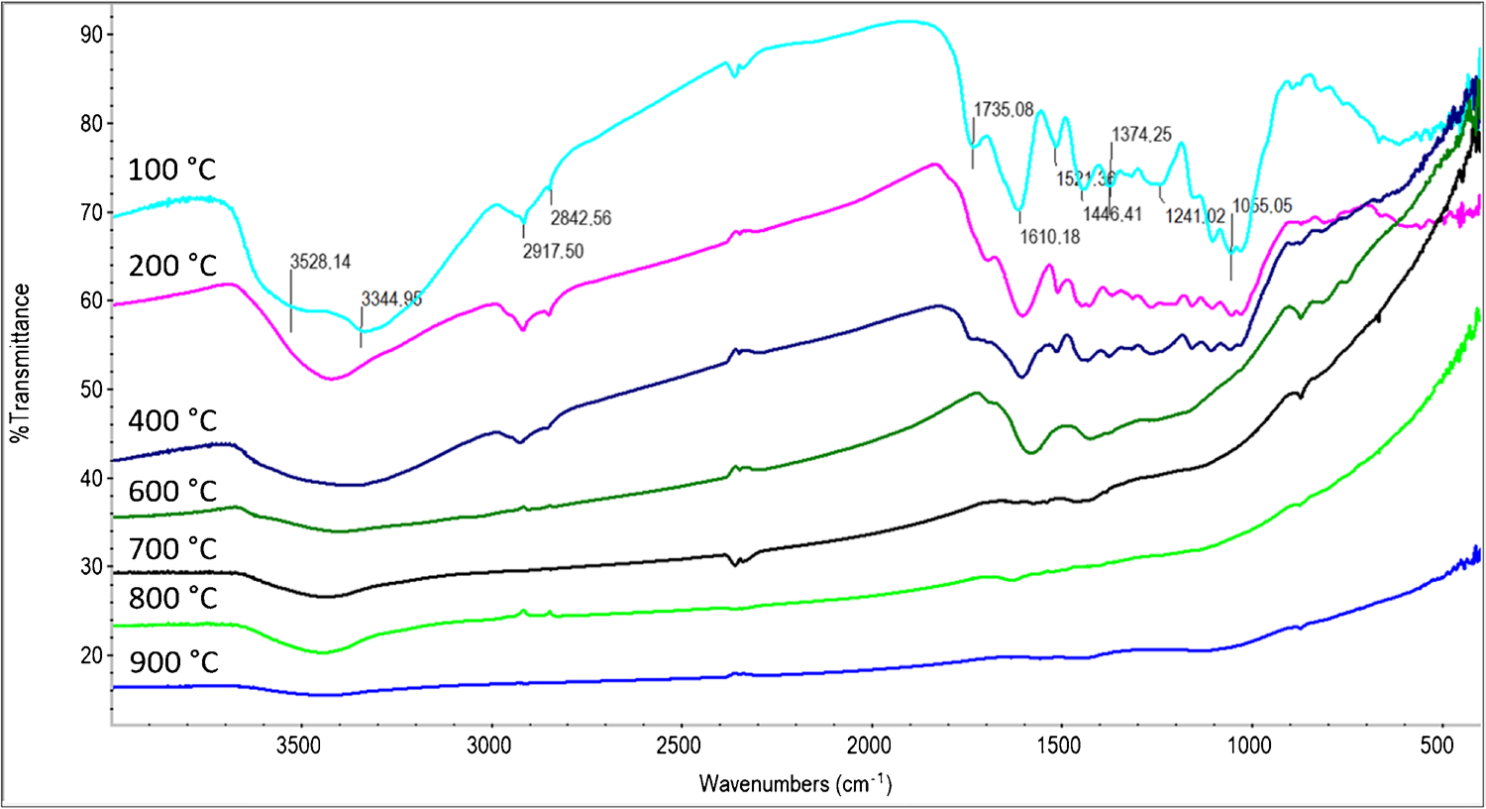
FTIR spectroscopy of temperature-dependent changes in the functional groups of hazelnut shells

**Fig. 3:**
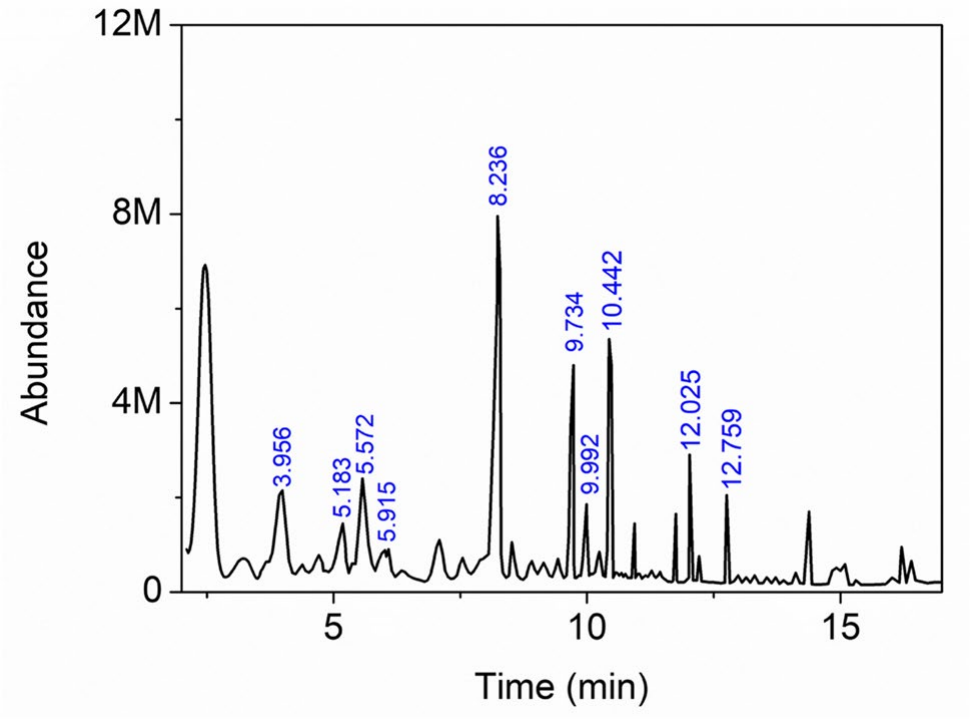
Total ion chromatogram of the distillate from hazelnut shell at 500 °C

For the carbonization of hazelnut shells, 100 g of shells was placed in a stainless-steel reactor. This process was repeated at each temperature, with carbonization performed separately at 200, 300, 400, 500, 600, 700, 800, 900, and 1000 °C for 1 h. The hazelnut shell mass loss at each temperature was determined, and the results are given in Fig. 1. Figure 1 illustrates the mass loss of hazelnut shells at each temperature. At 100 °C, the total mass loss ratio was 4.9%, corresponding to the moisture content. When the temperature was increased to 200 °C, the mass loss ratio was 40.8%. FTIR spectra of hazelnut shells treated at 100 and 200 °C showed no significant difference, except for a peak near 3600–3300 cm⁻^1^ (Fig. 2) [51]. This peak indicates the stretching of bonds between the oxygen and hydrogen atoms of phenolic groups. Since there are many phenolic groups in the lignin structure, it can be concluded that these groups undergo thermal cracking at 200 °C. The total mass loss during carbonization at 300 °C was 60.5%. Corresponding to the decreases in the peaks at 2917 and 2843 cm⁻^1^, which belong to aliphatic groups, decreases were also observed in the peaks in the 1735–1000 cm⁻^1^ region. In this region, the vibrations of aromatic C=C bonds correspond to the range of 1550–1350 cm⁻^1^, the peak at 1735 cm⁻^1^ corresponds to the stretching vibrations of the C=O bonds of carbonyl groups, and the peak at 1241 cm⁻^1^ corresponds to the vibrations of aromatic C-O bonds [51]. After the carbonization temperature reached 400 °C, the mass loss increase was less than at the previous temperature (total 65.2%). At this temperature, the intensities of the aliphatic C-H stretching peaks at 2917 and 2843 cm⁻^1^ decreased significantly, remaining only as weak shoulder bands. These peaks almost entirely vanished at higher temperatures, indicating the advanced stage of the carbonization process. On the other hand, the distillate formed during carbonization of hazelnut shells at 500 °C was collected and analyzed by GC-MS (Fig. 3). According to the library search results in ChemStation software, possible chemical compounds are shown in Table 1. Table 1 shows that most of the distillate products consist of phenol or phenol-derived compounds [52]. These results indicate that thermal cracking occurred predominantly in lignin structures. Finally, at higher temperatures, the mass loss remained almost constant, reaching a total of 67.2% at 900 °C. When the FTIR spectra corresponding to these temperatures were evaluated, the peaks in the 1600–1000 cm⁻^1^ region gradually decreased with increasing temperature and completely disappeared after 700 °C. This can be considered an indicator of further carbonization.

**Table 1:** The top 10 compounds with the highest relative abundance identified by the instrument’s software

No.	*t*_*R*_	Compound	Possibility	Area, %
1	3.956	Phenol	90	4.90
2	5.183	2-Methyl-phenol	97	2.87
3	5.572	p-Cresol	97	4.81
4	5.915	2-Methoxy-phenol	95	2.19
5	8.236	Catechol	91	10.59
6	9.734	3-Methyl-1,2-benzenediol	95	5.34
7	9.992	Hydroquinone	91	1.87
8	10.442	4-Methyl-1,2-benzenediol	97	4.12
9	12.025	2,6-Dimethoxy-phenol	97	3.91
10	12.759	4-Methoxy-1,3-benzenediamine	86	2.08

### Surface area and particle size effect

In this section, the relationship between the surface area of carbonized hazelnut shells, carbonization temperature, and particle size was investigated. The results showed a clear variation in the surface area depending on these two parameters. To determine the surface areas of heat-treated hazelnut shells, surface areas were determined using the MB-Langmuir method with three distinct particle size fractions: 0.420–0.240 mm, 0.240–0.125 mm, and 0.125–0.075 mm (Table 2). Initially, an amount of approximately 20 mg of AC was utilized for all particle size ranges. However, the regression coefficients for the 0.420–0.240 mm fraction were consistently below 0.95. This deviation was attributed to the inherent heterogeneity of the small sample amount. Consequently, upon increasing the AC amount to 50 mg, we were able to successfully achieve regression coefficients consistently exceeding 0.95 (S2). Table 2 demonstrates that a smaller particle size is associated with a higher regression coefficient. A similar situation was observed for the ACs obtained at each temperature. For example, at 200 °C, the AC surface area was 140 m^2^/g for the 0.420–0.240 mm fraction, while it was 150 m^2^/g for the 0.240–0.125 mm fraction and 152 m^2^/g for the 0.125–0.075 mm fraction (S3 and S4). Thermal carbonization alone was sufficient to improve the surface area of the AC to 155 m^2^/g at 900 °C. However, this value was further enhanced through a grinding process: the surface area increased to 165 m^2^/g for the 0.240–0.125 mm fraction and to 196 m^2^/g for the 0.125–0.075 mm fraction [53]. When evaluating the AC surface area as a function of temperature for the 0.420–0.240 mm fraction, a sharp decrease was observed at 300 °C. The surface area then remained relatively stable until 700 °C, after which it began to increase. This result is normal, as the GC-MS analysis of the distillate and the FTIR analysis of the AC both supported this observation. This is because thermal cracking causes the removal of numerous functional groups from the hazelnut body that influence adsorption. At higher temperatures, the continuation of thermal cracking led to the formation of pores in the structure that are effective for adsorption, and thus the surface area increased again [54].

**Table 2:** Temperature-dependent surface area of AC by MB and Langmuir equation

Temp. (°C)	Langmuir constants														
	0.420–0.240 mm					0.240–0.125 mm					0.125–0.075 mm				
	*K*_L_	Slope (g/mg)	*Q*_max_ (mg/g)	AC surface (m^2^/g)	*R*^2^	*K*_L_	Slope (g/mg)	*Q*_max_ (mg/g)	AC surface (m^2^/g)	*R*^2^	*K*_L_	Slope (g/mg)	*Q*_max_ (mg/g)	AC surface (m^2^/g)	*R*^2^
200	0.012	0.0157	63.7	140	0.9567	0.425	0.0147	68.0	150	0.9866	2.266	0.0145	69.0	152	0.9972
300	0.097	0.0670	14.9	33	0.9725	0.074	0.0422	23.7	52	0.9866	0.156	0.0407	24.6	54	0.9976
400	0.048	0.0686	14.6	32	0.9622	0.066	0.0470	21.3	47	0.9862	0.120	0.0430	23.3	51	0.9968
500	0.051	0.0550	18.2	40	0.9692	0.414	0.0490	20.4	45	0.9888	0.241	0.0415	24.1	53	0.9975
600	0.541	0.0669	14.9	33	0.9575	0.205	0.0550	18.2	40	0.9873	0.286	0.0500	20.0	44	0.9977
700	0.030	0.0450	22.2	49	0.9607	0.132	0.0328	30.5	67	0.9854	0.187	0.0128	78.1	172	0.9987
800	0.021	0.0155	64.5	142	0.9744	0.704	0.0145	69.0	152	0.9901	0.098	0.0121	82.6	182	0.9984
900	0.013	0.0142	70.4	155	0.9650	0.110	0.0134	74.9	165	0.9882	0.120	0.0112	89.3	196	0.9988
900^a^	0.159	0.0088	114.0	250	0.9845	0.038	0.0038	263.2	579	0.9962	0.146	0.0032	312.5	688	0.998

^a^Water vapor/AC ratio 1

### Steam activation and its effects

Further, the surface area of the AC was also improved by the concurrent application of heat and steam, and the results are presented in Table 2. For this process, a temperature of 900 °C was chosen, as it was determined from previous pyrolysis studies. The study observed a significant increase in the surface area of the synthesized AC when the steam/AC ratio (w/w) was set to 1, compared to the previous work without steam. The material was ground and sieved into fractions of 0.420–0.240 mm, 0.240–0.125 mm, and 0.125–0.075 mm. The corresponding surface areas were determined to be 250 m^2^/g, 579 m^2^/g, and 688 m^2^/g, respectively. While steam increased the surface area, it also decreased the AC yield. For instance, when the steam/AC ratio (w/w) was 1, the 32.8% pyrolysis yield fell significantly to 10%. When the same ratio was 1.5 and 2.0, the yields dropped to 6.3% and 3.6%, respectively. On the other hand, for the 0.420–0.240 mm fraction, the surface areas for ratios of 1.5 and 2.0 increased to 278 m^2^/g and 312 m^2^/g, respectively. It can be said that the low yield clearly overshadowed the appeal of the high surface area. Therefore, a ratio of 1 was selected for further VOC analysis, as it provided the optimum balance between high yield and high surface area. The BET value was measured as 1108 m^2^/g, which corresponded to the 688 m^2^/g surface area of the 0.125–0.075 mm fraction produced using this ratio. Similarly, the BET surface areas corresponding to the 579 m^2^/g and 250 m^2^/g surface areas for the 0.240–0.125 mm and 0.420–0.240 mm fractions were found to be 820 m^2^/g and 670 m^2^/g, respectively. The observation of a significant difference between MB-Langmuir and BET measurements is an expected outcome, considering the vast disparity in the molecular cross-sectional areas of methylene blue (130 Å^2^) and nitrogen gas (16.2 Å^2^). Although the smaller 0.125–0.075 mm fraction offers a superior surface area of 1108 m^2^/g, it was not selected because it increased the resistance to gas flow, which is a practical limitation. The larger particle size (0.240–0.125 mm) provides an optimal balance between a high surface area suitable for adsorption and the necessary operational conditions for VOC analysis.

In addition, high-ratio steam usage was also observed to reduce the surface areas of the micropores [55]. The micropore surface areas for the 0.240–0.125 mm particle size range, which was selected for a VOC study, were found to be 702, 639, and 540 m^2^/g for the ratios 1.0, 2.5, and 2.0, respectively (S5). Additionally, the total surface area values corresponding to these micropore surface areas were measured as 820, 926, and 1050 m^2^/g, respectively. Finally, the high micropore surface area of the 0.240–0.125 mm fraction, combined with its low back pressure, was a key factor in its selection.

### Morphological analysis (SEM)

**Fig. 4:**
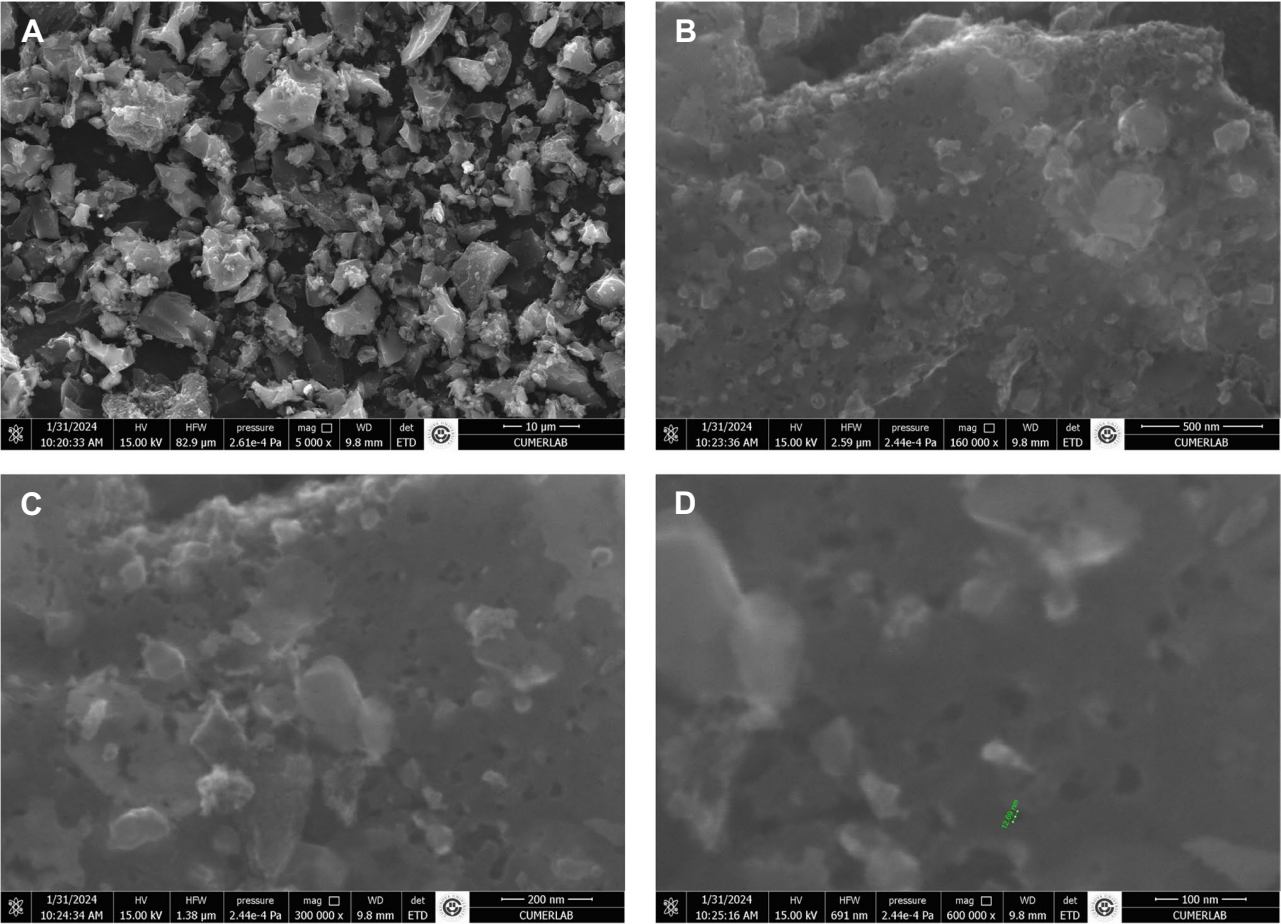
SEM images of the samples at different magnifications: **A** 5000× (S12), **B** 160,000× (S13), **C** 300,000× (S14), and **D** 600,000× (S15)

In Fig. 4, the scanning electron microscope (SEM) image of this AC is presented. According to Fig. 4A, the AC surface exhibits a highly rough and heterogeneous morphology. This irregular structure is a result of the high-temperature activation process and indicates the formation of a large surface area, which increases the AC’s adsorption capacity. Upon closer inspection (Fig. 4B), while the porous network structure is not yet distinct, with further magnification (Fig. 4C), the pores become clearly visible. The closest view of the AC can be seen in Fig. 4D, where the average pore diameter was measured as 12.69 nm. The macro- and mesopores facilitate the easier diffusion of molecules to the internal surfaces of the adsorbent, thus increasing the adsorption efficiency.

### Desorption of VOC from AC

**Fig. 5:**
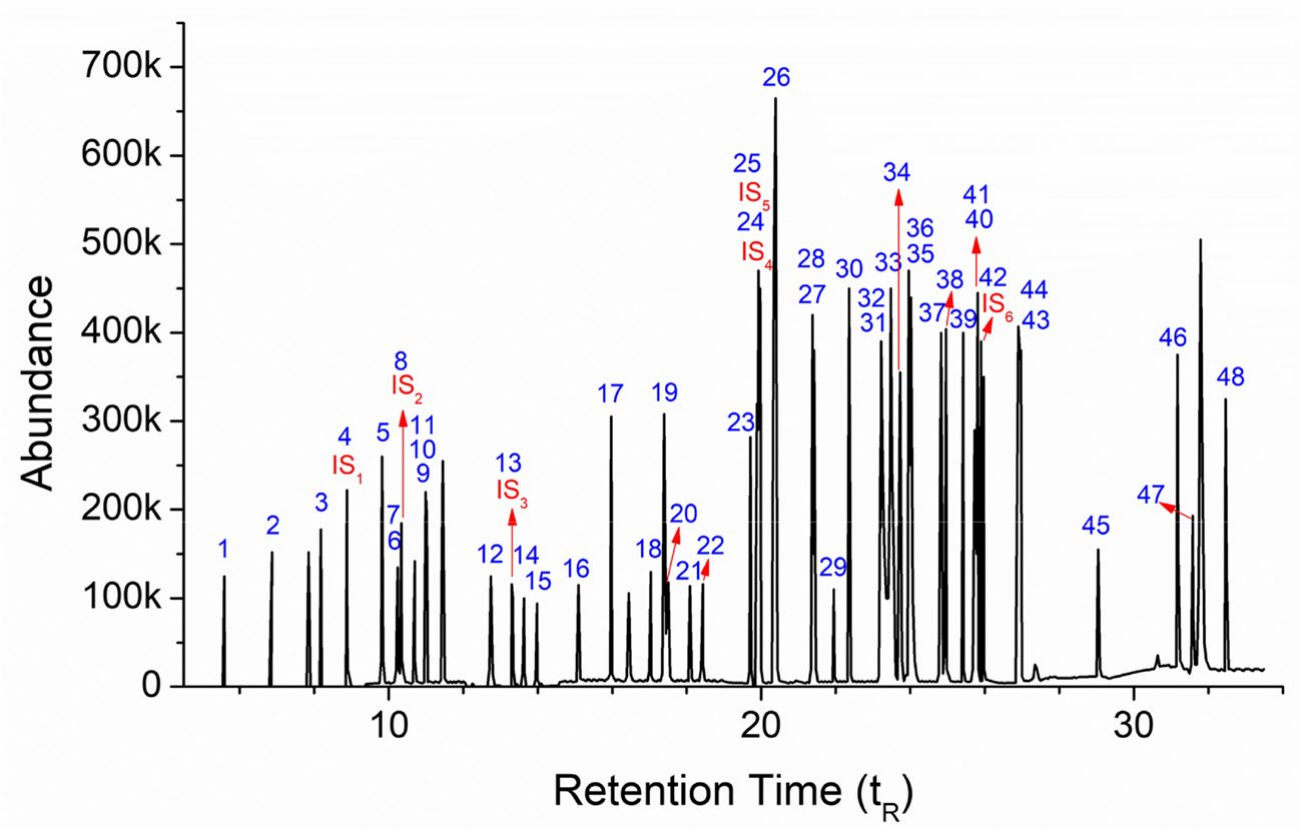
The total ion chromatogram of 48 VOCs

In this section, primarily, the determination of GC-MS analysis parameters to ensure satisfactory peak resolution for each VOC was carried out. The analytical parameters for the 48 target VOCs, including product ions, quantitation ions (Q_1_), signal-to-noise ratios (S/N), and retention times (*t*_R_), are comprehensively presented in Table 3. Based on these optimized setting values, the total ion chromatogram of 48 VOCs, obtained in under 33 min, is given in Fig. 5. These GC-MS setting values were used throughout the entire study. The experimental parameters for the desorption process, including solvent volume and contact time, were determined through preliminary optimization studies to ensure maximum recovery for all 48 target VOCs. These conditions were selected to provide the highest desorption efficiency while maintaining analytical precision. Recovery studies were examined for six different VOC concentrations dependent on spike levels for AC. The results of the 10 individual studies, along with their average recoveries, are provided in S.6. Plotting these recovery data for the 48 VOCs versus spike concentrations allowed us to evaluate the relationship between spike and recovery (desorption), where the slope corresponds to their average recoveries (S.7). These data also demonstrate the linearity of the applied method. With this acceptance criterion, an overall assessment of the VOC desorption results revealed that their recovery ratios, corresponding to the applied spike levels, ranged from 77.8% (n-butylbenzene) to 91.5% (1,1,1-trichloroethane and cis-1,2-dichloroethene), representing the lowest and highest values, respectively. Indeed, this is quite a good recovery range. Although Fabrizi et al. [46] successfully analyzed and reported on 57 different VOCs, they found that the recovery values for the vast majority of the VOCs ranged between 65 and 93%.

**Table 3:** Analytical parameters for VOCs: product ions, quantitation ions (Q_1_), signal-to-noise ratio (S/N), and retention times

No.	Compounds	*t*_R_	Product ions				Concentration (µg/L)	S/N for Q_1_	Concentration normalized for S/N 6 (µg/L)
			Q_1_	Q_2_	Q_3_	Q_4_			
1	Fluorotrichloromethane	5.873	101	103	105	66	100.0	18.0	33.3
2	1,1-Dichloroethene	6.872	61	96	98	63	100.0	12.0	50.0
3	*Trans*-1,2-dichloroethene	8.174	61	96	98	63	100.0	18.6	32.3
**IS**_**1**_	**1,2-Dichloroethane-*d***_***4***_	**8.878**	67	**69**	**85**	**89**	**100.0**	**14.1**	**42.5**
4	1,2-Dichloroethane	8.882	63	65	83	85	100.0	15.2	39.5
5	*Cis*-1,2-dichloroethene	9.815	77	61	96	98	100.0	7.6	85.7
6	Bromochloromethane	10.237	130	49	128	93	100.0	13.0	46.2
**IS**_**2**_	**Trichlromethane-*d***_***1***_	**10.340**	84	**86**	**47**	**88**	**100.0**	**13.1**	**45.8**
7	Trichlromethane	10.344	83	85	47	87	100.0	21.3	28.1
8	1,1,1-Trichloroethane	10.695	97	99	61	117	100.0	21.0	28.6
9	1,1-Dichloro-1-propene	10.986	75	117	119	110	100.0	14.3	42.0
10	Benzene	11.008	77	91	43	52	100.0	13.2	45.4
11	Tetrachloromethane	11.016	117	119	75	121	100.0	11.1	54.1
12	Trichloroethene	12.741	132	130	95	97	100.0	15.7	38.2
**IS**_**3**_	**1,2-Dichloropropane-*d***_***6***_	**13.302**	69	**68**	**82**	**49**	**100.0**	**8.1**	**74.1**
13	1,2-Dichloropropane	13.329	63	62	76	41	100.0	7.2	83.3
14	Dibromomethane	13.632	174	93	95	172	100.0	21.4	28.0
15	Bromodichloromethane	13.977	83	85	47	129	100.0	14.5	41.4
16	1,3-Dichloropropene (*cis*+*trans*)	15.095	75	39	77	110	100.0	8.6	69.8
17	Toluene	15.969	91	92	65	39	100.0	24.2	24.8
18	1,1,2-Trichloroethane	17.034	97	83	99	85	100.0	6.6	90.9
19	Tetrachloroethene	17.396	166	164	129	131	100.0	20.8	28.8
20	1,3-Dichloropropane	17.497	76	41	78	39	100.0	8.6	69.8
21	Dibromochloromethane	18.092	129	127	131	81	100.0	15.6	38.5
22	1,2-Dibromoethane	18.437	107	109	79	93	100.0	12.0	50.0
23	Chlorobenzene	19.705	112	77	114	51	100.0	43.1	13.9
**IS**_**4**_	**1,1,1,2-Tetrachloroethane-*d***_***2***_	**19.892**	133	**135**	**119**	**121**	**100.0**	**9.3**	**64.5**
24	1,1,1,2-Tetrachloroethane	19.923	131	133	117	119	100.0	8.2	73.2
**IS**_**5**_	**Ethylbenzene-*d***_***10***_	**19.971**	101	**116**	**87**	**102**	**100.0**	**32.4**	**18.5**
25	Ethylbenzene	19.977	91	106	77	92	100.0	31.5	19.0
26	*p,m*-Xylene	20.292	91	106	105	77	200.0	30.3	39.6
27	*o*-Xylene	21.380	91	106	105	77	100.0	25.3	23.7
28	Styren	21.422	104	103	78	77	100.0	16.2	37.0
29	Tribromomethane	21.951	173	171	175	93	100.0	14.9	40.3
30	Isopropylbenzene	22.361	105	120	77	79	100.0	31.4	19.1
31	1,1,2,2-Tetrachloroethane	23.200	83	85	131	95	100.0	8.5	70.6
32	Bromobenzene	23.217	77	156	158	51	100.0	10.8	55.6
33	*n*-Propylbenzene	23.479	91	120	92	65	100.0	21.6	27.8
34	2-Chlorotoluene	23.729	91	126	89	128	100.0	10.1	59.4
35	1,3,5-Trimethylbenzene	23.955	105	120	119	77	100.0	11.7	51.3
36	4-Chlorotoluene	24.020	91	126	125	128	100.0	18.3	32.8
37	*Tert*-butylbenzene	24.829	119	91	134	77	100.0	35.8	16.8
38	1,2,4-Trimethyl Benzene	24.960	105	120	119	77	100.0	13.3	45.1
39	*Sec*-butylbenzene	25.417	105	134	91	77	100.0	17.9	33.5
40	1,3-Dichlorobenzene	25.732	146	148	111	75	100.0	37.5	16.0
41	4-Isopropyltolune	25.810	119	134	91	117	100.0	16.7	35.9
**IS**_**6**_	**1,4-Dichlorobenzene-*d***_***4***_	**25.901**	150	**152**	**115**	**49**	**100.0**	**40.5**	**14.8**
42	1,4-Dichlorobenzene	25.970	146	148	111	75	100.0	42.0	14.3
43	*n*-Butylbenzene	26.898	91	92	134	65	100.0	16.5	36.4
44	1,2-Dichlorobenzene	26.969	146	148	111	75	100.0	32.9	18.2
45	1,2-Dibromo-3-chloro propane	29.040	157	155	75	39	100.0	6.3	95.2
46	1,2,4-Trichlorobenzene	31.167	180	182	145	109	100.3	27.1	22.2
47	Hexachloro-1,3-butha diene	31.577	225	227	223	190	100.0	11.6	51.7
48	1,2,3-Trichlorobenzene	32.463	180	182	184	145	100.0	18.4	32.6

Twenty-three out of the 48 VOCs are benzene-derived compounds. Within this group, n-butylbenzene showed the lowest recovery value of 77.8%, while sec-butylbenzene displayed the highest recovery value of 87.1%. Among these benzene-derived compounds, 14 have alkyl substitutions, and the remaining structures are halogen-substituted. The recovery rates for alkyl-substituted VOCs were observed to range between 77.8 and 87.1%, and for halogen-substituted VOCs, between 81.5 and 86.7%. Considering these results, it can be stated that there was no significant difference in recovery between the two groups in the adsorption-desorption study. Among the remaining 25 halogen-substituted VOCs, 8 were identified as alkenes and 17 as alkanes. The recovery rates for alkenes ranged from 81.0 to 91.5%, while those for alkanes ranged from 79.8 to 91.5%.

Compared to the study by Li et al. [22], where their synthesized adsorbent achieved approximately 76% adsorption of toluene (2300 mg/L), the hazelnut shell–derived adsorbent in this study demonstrated a higher adsorption rate of 84.5% for toluene. In addition, it was observed that the recovery values for some chlorinated compounds (1,1,2,2-tetrachloroethane, 1,3-dichloropropane, 1,2,4-trichlorobenzene, 1,2-dichlorobenzene, 1,3-dichlorobenzene, and 2-chlorotoluene) were higher when compared to some researchers’ obtained results by using commercial AC, which SKC brand coconut shell originated [46].

In this study, to compare the synthesized AC with the CAC, studies were conducted at concentrations of 150 and 2400 µg/L. The results obtained in both studies yielded quite similar values; however, it was observed that positive recovery values (over 100%) were less frequent in the CAC studies (S8).

### Verification of desorption of VOC from AC

#### Comparison of method and instrumental detection limits

In this section, the LOD values for all 48 VOCs were determined and are comprehensively presented in Table 4. The method’s success in achieving exceptionally low LOD values underscores its high effectiveness and sensitivity. Notably, these values are sufficiently low to meet and often exceed the VOC regulatory limits [56] established in numerous countries, thereby confirming the method’s broad applicability and practical utility.

**Table 4:** Comparison of LOD_Met_ values with LOD_GC-MS_ in the desorption of VOCs

No.	VOCs	For LOD_Met_ (µg/L)				LOD_GC/MS_ (µg/L)	LOD_Met_/LOD_GC-MS_
		Spike	*Rec*_Aver_	*Std*	LOD_Met_		
1	Fluorotrichloromethane	50.0	36.46	1.62	41.31	33.31	1.2
2	1,1-Dichloroethene	60.0	58.19	3.02	67.23	50.00	1.1
3	*Trans*-1,2-dichloroethene	50.0	43.43	2.02	49.49	32.32	1.5
4	1,2-Dichloroethane	50.0	43.17	1.51	47.69	39.51	1.0
5	*Cis*-1,2-dichloroethene	90.0	86.33	3.42	96.58	85.65	1.1
6	Bromochloromethane	50.0	48.25	2.75	56.51	46.20	1.2
7	Trichlromethane	30.0	28.94	1.41	33.17	28.12	1.2
8	1,1,1-Trichloroethane	50.0	47.77	2.53	55.35	42.08	1.3
9	1,1-Dichloro-1-propene	50.0	49.40	1.51	53.91	42.00	1.1
10	Benzene	50.0	46.73	4.42	60.00	45.44	1.2
11	Tetrachloromethane	60.0	55.08	2.65	63.04	54.05	1.1
12	Trichloroethene	50.0	49.49	2.22	56.16	38.21	1.5
13	1,2-Dichloropropane	90.0	86.83	3.42	97.08	83.34	1.2
14	Dibromomethane	30.0	28.08	2.42	35.35	28.03	1.3
15	Bromodichloromethane	50.0	45.48	4.72	59.64	41.48	1.4
16	1,3-Dichloropropene (*cis*+*trans*)	90.0	87.03	7.24	108.74	69.80	1.6
17	Toluen	30.0	31.42	1.73	36.62	24.83	1.4
18	1,1,2-Trichloroethane	120.0	117.28	10.45	148.64	100.06	1.5
19	Tetrachloroethene	30.0	28.68	1.82	34.14	28.82	1.2
20	1,3-Dichloropropane	90.0	84.44	8.84	110.94	69.81	1.6
21	Dibromochloromethane	50.0	43.22	1.31	47.13	38.59	1.2
22	1,2-Dibromoethane	60.0	58.75	5.51	75.28	50.09	1.5
23	Chlorobenzene	15.0	13.94	1.21	17.57	13.95	1.3
24	1,1,1,2-Tetrachloroethane	90.0	80.70	6.73	100.90	73.22	1.4
25	Ethylbenzene	25.0	22.02	1.52	26.56	19.05	1.4
26	*p,m*-Xylene	50.0	46.38	3.31	56.32	39.61	1.4
27	*o*-Xylene	30.0	26.43	2.41	33.67	23.76	1.4
28	Styren	50.0	43.04	2.24	49.78	37.03	1.3
29	Tribromomethane	50.0	46.73	3.32	56.68	40.38	1.4
30	Isopropylbenzene	25.0	21.51	1.72	26.66	19.18	1.4
31	1,1,2,2-Tetrachloroethane	90.0	86.75	5.72	103.91	70.65	1.5
32	Bromobenzene	60.0	57.59	4.72	71.76	55.62	1.3
33	*n*-Propylbenzene	30.0	27.54	2.35	34.58	27.78	1.1
34	2-Chlorotoluene	60.0	60.60	5.35	76.66	59.43	1.3
35	1,3,5-Trimethylbenzene	60.0	51.66	4.82	66.13	51.32	1.3
36	4-Chlorotoluene	50.0	52.12	4.65	66.05	32.87	2.0
37	*Tert*-butylbenzene	25.0	26.91	1.71	32.03	16.81	1.9
38	1,2,4-Trimethylbenzene	50.0	49.04	3.22	58.69	45.13	1.3
39	*Sec*-butylbenzene	50.0	45.70	3.47	56.10	33.53	1.6
40	1,3-Dichlorobenzene	25.0	23.62	2.21	30.25	16.02	1.9
41	4-Isopropyltolune	50.0	43.63	1.62	48.48	35.90	1.3
42	1,4-Dichlorobenzene	25.0	17.27	0.70	19.38	14.33	1.3
43	*n*-butylbenzene	50.0	48.44	2.21	55.07	36.46	1.5
44	1,2-Dichlorobenzene	25.0	26.72	1.33	30.70	18.28	1.7
45	1,2-Dibromo-3-chloropropane	120.0	110.60	8.99	137.56	95.21	1.4
46	1,2,4-Trichlorobenzene	25.0	23.32	1.31	27.24	22.26	1.2
47	Hexachloro-1,3-buthadiene	60.0	51.71	2.73	59.89	51.78	1.1
48	1,2,3-Trichlorobenzene	50.0	40.96	1.41	45.18	32.69	1.4

**Fig. 6:**
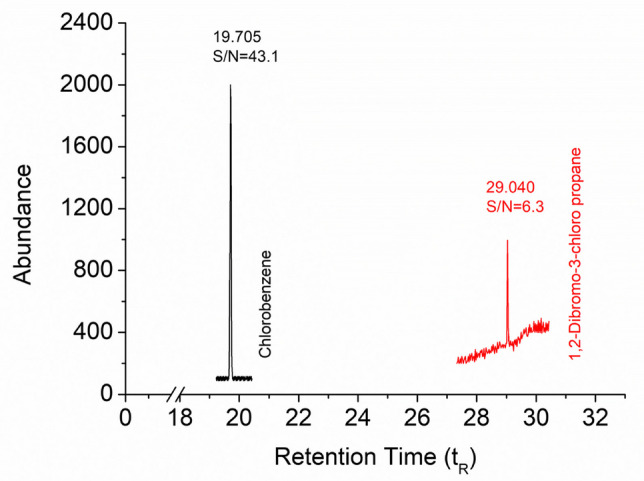
Comparison of chromatograms at 100 µg/dm^3^, highlighting chlorobenzene and 1,2-dibromo-3-chloropropane with the highest and lowest S/N ratios, respectively

In determining the LOD for the applied method, the objective is to achieve the lowest possible values. These low values are fundamentally constrained by the detection capability of the instrumental device and the recovery efficiency of the method. Consequently, the method’s LOD should ideally approach the minimum measurable value of the instrument. In this section, a comparison was also made between the successful method applied and the instrument. Therefore, the LOD values of the applied method (LOD_Met_) were compared to those of the GC-MS instrument (LOD_GC-MS_) for each VOC. The results of these LOD studies are given in Table 4. The purpose of this is to first determine the instrument’s LOD for a given VOC, in order to prevent the observation of values lower than the instrument’s LOD during method applications. In other words, it helps to avoid “ghost recovery.” The chromatograms of 1,2-dibromo-3-chloropropane (lowest S/N ratio) and chlorobenzene (highest S/N ratio) at a 100 µg/dm^3^ concentration are illustrated in Fig. 6. As presented in Table 4, a proportional comparison between LOD_Met_ and LOD_GC-MS_ showed significant differences, with LOD_Met_/LOD_GC-MS_ ratios ranging from 1.0 to 2.0. While the LOD_Met_/LOD_GC-MS_ ratios were calculated to be 1.1 for 6 of the VOCs, these ratios varied 1.2 for 8 of the VOCs. For these specific 14 VOCs, the experimentally obtained LOD_Met_ values demonstrated satisfactory sensitivity, as the method-specific LOD incurred a maximum of only 20% loss of sensitivity relative to the LOD_GC-MS_. On the other hand, while the ratios for 20 VOCs ranged from 1.3 to 1.4, these ratios for 9 VOCs varied between 1.5 and 1.6. The remaining 5 VOCs, on the other hand, showed ratios ranging from 1.7 to 2.0. As a result, the lowest LOD value of 13.95 µg/L stated in Table 4 belongs to chlorobenzene, and its concentration in 15 L of air was found to be 3.72 µg/m^3^ when dilution factors are also taken into account. Fabrizi et al. [46] have stated that the lowest LOD value of 6 µgm^−3^ belongs to trichloroethylene. However, they determined the S/N ratio as 3. When compared with the results of this study, this value increases to the 12 µg/m^3^ level when S/N is 6. The LOD value for this compound is 49.49 (Table 4), and when calculated for 15 L of air, it is found to be 13.20 µg/m^3^, which means that almost the same value is reached in two different studies. Although many researchers prefer an S/N of 3, an S/N of 6 was selected to ensure greater reliability in this study.

#### Linearity of desorption methods

The study also examined the linearity of the desorption method’s recovery values for each of the 48 VOCs at different spike levels. When recovery values are plotted against their respective spike concentrations across six levels for each VOC, the slope of the resulting line represents the average recovery of linearity (*R*_Mean-L_). This *R*_Mean-L_ value can then be interpreted as the percentage recovery for these measurements (S.7). The lowest and highest *R*_Mean-L_ values obtained using the CS_2_ desorption method were 77.8% for n-butylbenzene and 91.5% for 1,1,1-trichloroethane and cis-1,2-dichloroethene, respectively. The *R*_Mean-L_ values for 48 VOCs can be categorized into three levels: high (90–91.5%), moderate (85–89%), and low (78–84%). Three of the VOCs exhibited high *R*_Mean-L_ values, while 21 showed moderate *R*_Mean-L_ values. The remaining 24 VOCs had low *R*_Mean-L_ values. No aromatic compounds were observed within the high *R*_Mean-L_ group. Separately, it was observed that three highly chlorinated VOCs (composed of ethane, ethene, and methane derivatives), such as 1,1,1-trichloroethane, showed the highest overall recovery ratios.

#### The effect of AC on method performance: repeatability, reproducibility, and accuracy

Repeatability studies were conducted by three analysts, each performing five replicates at two concentrations: 100.0 µg/L and 1000.0 µg/L. For all VOCs, the percent relative standard deviations (*RSDr*) was consistently below 20%. The *RSDr* values for Analyst I, Analyst II, and Analyst III at the 100 µg/L VOC concentration were found to be 4–4%, 4–14%, and 4–15%, respectively. At 1000 µg/L, the corresponding values were 4–13%, 3–14%, and 4–13%, respectively. Since all *RSDr* values were less than 20%, there was no significant difference among the results (S.9). Similarly, reproducibility studies were conducted over 5 days by three analysts at the same two concentrations. The percent relative standard deviation of reproducibility (*RSD*_*WR*_) values was observed to be below 20%. The *RSD*_*WR*_ values obtained from the analysts ranged from 1.51 to 14.32% for 100.0 µg/L and 1.11 to 15.25% for 1000.0 µg/L. Concurrently, the intra-day *RSD*_*WR*_ values were found to be 3.52 to 14.99% for 100.0 µg/L and 2.56 to 14.72% for 1000.0 µg/L. As all *RSD*_*WR*_ values met the criterion of being less than 20%, there was no significant difference among the results (S10). In conclusion, the AC produced from hazelnut shells and used in VOC determination has a high degree of homogeneity. Due to this high homogeneity, the repeatability and reproducibility values remained below 20%.

The accuracy of the applied method was tested using a Certified Reference Material (CRM) containing 32 VOCs of known concentrations. The measured values for these concentrations were in good agreement with the certified values. For these VOCs, the *Z*-score ranged from − 0.63 to + 0.61. This range indicates the absence of a systematic error and shows no significant difference between the known concentrations of the CRM and the results obtained by our method (S.11). These results demonstrated that the synthesized AC can be reliably utilized in VOC analysis methods.

## Conclusion

The findings of this study demonstrate that activated carbon (AC) synthesized from hazelnut shells, a sustainable waste material, serves as a highly effective adsorbent for complex analytical applications. It was established that the surface properties can be precisely controlled by optimizing critical parameters such as particle size and steam ratio. The steam-assisted activation process was shown to significantly enhance the surface area and micropore structure compared to conventional carbonization, providing an ideal framework for VOC capture.

The methodology developed in this study meets the high sensitivity requirements for the quantitative analysis of 48 different VOCs. Even with a more stringent signal-to-noise (S/N) criterion compared to existing literature, the method proved to be exceptionally reliable and reproducible. A comparison with commercial activated carbon (CAC) indicated that the synthesized AC provides competitive performance, particularly in terms of recovery efficiency and practical applicability in environmental monitoring.

Furthermore, the analytical limits achieved in this work align with international regulatory standards for VOC emissions, confirming the potential of hazelnut-shell-derived AC as a cost-effective and sustainable alternative to commercial sorbents. Future research may expand the versatility of this bio-based adsorbent by investigating its performance across a broader range of pollutants and diverse environmental matrices.

## Supplementary Information

Below is the link to the electronic supplementary material.

Supplementary file1 (DOCX 23.7 MB)

Supplementary file2 (DOCX 20.5 MB)

## Data Availability

The data that support the findings of this study are available from the corresponding author upon reasonable request.
