# Supplementary material for: Using activated carbon produced from hazelnut shells as an adsorbent for the quantitative analysis of volatile organic compounds by GC-MS
Source: Anal Bioanal Chem. 2026 Feb 18;418(9):2669–84. doi: 10.1007/s00216-026-06394-5 (PMC13079479; doi:10.1007/s00216-026-06394-5)
Supplement: Supplementary file 1 — Supplementary file1 (DOCX 23.7 MB) [file 216_2026_6394_MOESM1_ESM.docx]

| 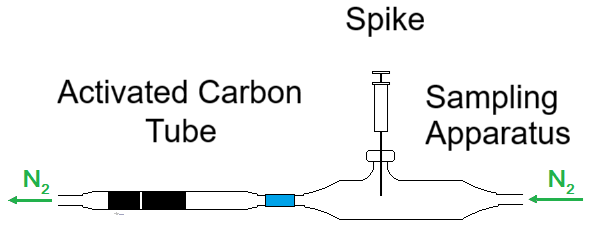 |
| --- |
| **S.1**.Sampling apparatus and spiking to AC |





|  |
| --- |
| **S.2.** Monitoring of the surface area change with temperature of the 0.420-0.240 mm particle size fraction of AC with *MB*-Langmuir. |

|  |
| --- |
| **S.3.** Monitoring of the surface area change with temperature of the 0.240-0.125 mm particle size fraction of AC with *MB*-Langmuir. |

|  |
| --- |
| ****  **S.4.** Monitoring of the surface area change with temperature of the 0.125-0.075 mm particle size fraction of AC with *MB*-Langmuir. |

|  |
| --- |
| **S.5.** Monitoring the effect of particle size fractions on the surface area of AC produced at 900°C using the *MB*-Langmuir method. |

| **S.6.** Spike levels, Averages of Recoveries (**R_Mean_**), Percents of Recoveries (**R_Mean_** %), Percent of Standard Deviations (STD %) in CS_2_ desorption process. | | | | | | | | | | |
| --- | --- | --- | --- | --- | --- | --- | --- | --- | --- | --- |
| **No** | **VOCs** | **Spike (µg/L)** | **R_Mean_ (µg/L)** | **R_Mean_**  **(%)** | **STD (%)** | **No** | **VOCs** | **R_Mean_**  **( µg/L )** | **R_Mean_ (%)** | **STD (%)** |
| 1 | Fluorotricholoro methane | 75.0  150.0  300.0  600.0  1200.0  2400.0 | 62.7 | 83.6 | 11.8 | 11 | Tetrachloro methane | 64.6 | 86.1 | 10.2 |
|  |  |  | 125.3 | 83.5 | 10.5 |  |  | 143.2 | 95.5 | 7.2 |
|  |  |  | 255.8 | 85.3 | 7.1 |  |  | 259.8 | 86.6 | 7.8 |
|  |  |  | 552.2 | 92.0 | 8.0 |  |  | 607.1 | 101.2 | 6.6 |
|  |  |  | 1162.7 | 96.9 | 6.8 |  |  | 1105.6 | 92.1 | 8.7 |
|  |  |  | 2006.8 | 83.6 | 9 |  |  | 2185.8 | 91.1 | 6.5 |
| 2 | 1,1-dichloro  ethene |  | 61.6 | 82.1 | 9.2 | 12 | Trichloro ethene | 70.3 | 93.7 | 7.5 |
|  |  |  | 126.1 | 84.1 | 11.9 |  |  | 143.5 | 95.7 | 6.2 |
|  |  |  | 257.6 | 85.9 | 7.3 |  |  | 272.9 | 91.0 | 8.3 |
|  |  |  | 527.2 | 87.9 | 5.0 |  |  | 636.3 | 106.1 | 8.6 |
|  |  |  | 1138.7 | 94.9 | 3.6 |  |  | 1129.5 | 94.1 | 10.7 |
|  |  |  | 2058.2 | 85.8 | 4.8 |  |  | 1992.7 | 83.0 | 6.6 |
| 3 | *Trans*-1,2-dichloro  ethene |  | 60.8 | 81.1 | 6.3 | 13 | 1,2-dichloro propane | ND | ND | ND |
|  |  |  | 121.4 | 80.9 | 8.6 |  |  | 153.6 | 102.4 | 3.3 |
|  |  |  | 252.0 | 84.0 | 6.6 |  |  | 271.7 | 90.6 | 4.4 |
|  |  |  | 519.2 | 86.5 | 3.9 |  |  | 611.4 | 101.9 | 5.8 |
|  |  |  | 1081.7 | 90.1 | 5.1 |  |  | 1044.8 | 87.1 | 6.8 |
|  |  |  | 2033.9 | 84.7 | 8.1 |  |  | 2084.8 | 86.9 | 7.4 |
| 4 | 1,2-dichloro ethane |  | 68.2 | 90.9 | 5.6 | 14 | Dibromo methane | 72.1 | 96.1 | 3.8 |
|  |  |  | 141.3 | 94.2 | 3.4 |  |  | 146.8 | 97.9 | 8.4 |
|  |  |  | 252.6 | 84.2 | 9.4 |  |  | 267.5 | 89.2 | 8.4 |
|  |  |  | 576.9 | 96.2 | 8.1 |  |  | 604.1 | 100.7 | 7.0 |
|  |  |  | 1098.3 | 91.5 | 6.7 |  |  | 1021.8 | 85.2 | 5.9 |
|  |  |  | 2118.1 | 88.3 | 9.2 |  |  | 2056.4 | 85.7 | 8.0 |
| 5 | *Cis*-1,2-dichloro ethene |  | 68.4 | 91.2 | 9.4 | 15 | Bromodichloro  methane | 64.9 | 86.5 | 8.1 |
|  |  |  | 155.7 | 103.8 | 7.8 |  |  | 149.4 | 99.6 | 10.5 |
|  |  |  | 276.2 | 92.1 | 6.4 |  |  | 273.4 | 91.1 | 6.7 |
|  |  |  | 639.8 | 106.6 | 8.9 |  |  | 593.2 | 98.9 | 4.4 |
|  |  |  | 1145.9 | 95.5 | 9.2 |  |  | 1013.1 | 84.4 | 3.1 |
|  |  |  | 2241.2 | 93.4 | 8.1 |  |  | 2130.1 | 88.8 | 4.2 |
| 6 | Bromochloro methane |  | 67.8 | 90.4 | 7.0 | 16 | 1,3-dichloro propene (cis+trans) | 78.0 | 104.0 | 9.4 |
|  |  |  | 148.4 | 98.9 | 4.9 |  |  | 141.1 | 94.1 | 10.2 |
|  |  |  | 269.3 | 89.8 | 3.5 |  |  | 279.3 | 93.1 | 6.8 |
|  |  |  | 603 | 100.5 | 4.7 |  |  | 552.3 | 92.1 | 3.8 |
|  |  |  | 1078.2 | 89.9 | 6.1 |  |  | 1100.1 | 91.7 | 5.3 |
|  |  |  | 1923.9 | 80.2 | 8.2 |  |  | 2052.5 | 85.5 | 4.4 |
| 7 | Trichlromethane |  | 68.9 | 91.9 | 7.6 | 17 | Toluen | 69.2 | 92.3 | 6.6 |
|  |  |  | 153.0 | 102.0 | 7.0 |  |  | 161.1 | 107.4 | 7.5 |
|  |  |  | 277.7 | 92.6 | 7.7 |  |  | 300.3 | 100.1 | 3.3 |
|  |  |  | 605.9 | 101.0 | 6.5 |  |  | 645.5 | 107.6 | 4.4 |
|  |  |  | 1070.6 | 89.2 | 9.0 |  |  | 1119.6 | 93.3 | 5.9 |
|  |  |  | 2122.2 | 88.4 | 8.9 |  |  | 2077.6 | 86.6 | 8.1 |
| 8 | 1,1,1-trichloro ethane |  | 67.8 | 90.4 | 7.3 | 18 | 1,1,2-trichloro ethane | ND | ND | ND |
|  |  |  | 150.6 | 100.4 | 6.2 |  |  | 141.9 | 94.6 | 3.5 |
|  |  |  | 271.1 | 90.4 | 8.5 |  |  | 267.4 | 89.1 | 9.0 |
|  |  |  | 611.4 | 101.9 | 8.5 |  |  | 570.6 | 95.1 | 7.8 |
|  |  |  | 1127.3 | 93.9 | 10.8 |  |  | 969.4 | 80.8 | 6.4 |
|  |  |  | 2246.3 | 93.6 | 7.1 |  |  | 1957.1 | 81.5 | 8.8 |
| 9 | 1,1-dichloro-1-propene |  | 70.5 | 94.0 | 4.6 | 19 | Tetrachloro ethene | 62.9 | 83.9 | 3.2 |
|  |  |  | 133.7 | 89.1 | 3.3 |  |  | 137.5 | 91.7 | 4.6 |
|  |  |  | 253.3 | 84.4 | 6.4 |  |  | 255.3 | 85.1 | 5.5 |
|  |  |  | 557.6 | 92.9 | 6.9 |  |  | 579.3 | 96.6 | 8.2 |
|  |  |  | 1112.8 | 92.7 | 8.3 |  |  | 997.3 | 83.1 | 6.1 |
|  |  |  | 2008.2 | 83.7 | 6.1 |  |  | 1958.7 | 81.6 | 3.5 |
| 10 | Benzene |  | 66.7 | 88.9 | 6.4 | 20 | 1,3-dichloro propane | 64.6 | 86.1 | 6.8 |
|  |  |  | 147.6 | 98.4 | 10.9 |  |  | 143.3 | 95.5 | 7.8 |
|  |  |  | 259.9 | 86.6 | 7.3 |  |  | 259.8 | 86.6 | 4.4 |
|  |  |  | 635.7 | 106.0 | 8.4 |  |  | 607.7 | 101.3 | 7.3 |
|  |  |  | 1097.1 | 91.4 | 6.7 |  |  | 1105.6 | 92.1 | 3.2 |
|  |  |  | 2034.2 | 84.8 | 10.6 |  |  | 2085.8 | 86.9 | 6.2 |

| **S.6.** Continuous... | | | | | | | | | | |
| --- | --- | --- | --- | --- | --- | --- | --- | --- | --- | --- |
| **No** | **VOCs** | **Spike (µg/L)** | **R_Mean_ (µg/L)** | **R_Mean_**  **(%)** | **STD (%)** | **No** | **VOCs** | **R_Mean_**  **(µg/L)** | **R_Mean_ (%)** | **STD (%)** |
| 21 | Dibromochloro  methane | 75.0  150.0  300.0  600.0  1200.0  2400.0 | 74.1 | 98.8 | 9.9 | 31 | 1,1,2,2-tetrachloro ethane | ND | ND | ND |
|  |  |  | 170.9 | 113.9 | 6.8 |  |  | 143.7 | 95.8 | 3.3 |
|  |  |  | 309.2 | 103.1 | 7.5 |  |  | 288.1 | 96.0 | 4.6 |
|  |  |  | 607.4 | 101.2 | 6.4 |  |  | 572.3 | 95.4 | 6.1 |
|  |  |  | 1052.4 | 87.7 | 8.8 |  |  | 967.5 | 80.6 | 8.1 |
|  |  |  | 2080.0 | 86.7 | 6.4 |  |  | 2075.9 | 86.5 | 6.3 |
| 22 | 1,2-dibromo ethane ne |  | 78.7 | 104.9 | 7.8 | 32 | Bromo  benzene | 78.2 | 104.3 | 9.7 |
|  |  |  | 170.6 | 113.7 | 6.1 |  |  | 155.9 | 103.9 | 6.5 |
|  |  |  | 300.2 | 100.1 | 8.3 |  |  | 279.1 | 93.0 | 7.1 |
|  |  |  | 621.6 | 103.6 | 8.4 |  |  | 602.6 | 100.4 | 6.0 |
|  |  |  | 1065.1 | 88.8 | 10.6 |  |  | 978.0 | 81.5 | 8.2 |
|  |  |  | 2058.8 | 85.8 | 6.5 |  |  | 1993.4 | 83.1 | 6.8 |
| 23 | Chloro  benzene |  | 61.3 | 81.7 | 4.5 | 33 | *n*-propylbenzene | 64.6 | 86.1 | 9.0 |
|  |  |  | 136.1 | 90.7 | 3.2 |  |  | 146.4 | 97.6 | 11.4 |
|  |  |  | 275.3 | 91.8 | 4.3 |  |  | 274.5 | 91.5 | 7.0 |
|  |  |  | 591.1 | 98.5 | 5.8 |  |  | 579.3 | 96.6 | 4.9 |
|  |  |  | 965.9 | 80.5 | 7.7 |  |  | 963.6 | 80.3 | 3.5 |
|  |  |  | 2047.5 | 85.3 | 7.9 |  |  | 1993.4 | 83.1 | 4.7 |
| 24 | 1,1,1,2-tetrachloro  ethane |  | 72.2 | 96.3 | 6.4 | 34 | 2-chloro toluene | 72.7 | 96.9 | 10.8 |
|  |  |  | 170.6 | 113.7 | 9.3 |  |  | 145.6 | 97.1 | 3.8 |
|  |  |  | 301.4 | 100.5 | 9.3 |  |  | 265.3 | 88.4 | 4.1 |
|  |  |  | 610.2 | 101.7 | 7.5 |  |  | 547.9 | 91.3 | 5.5 |
|  |  |  | 956.1 | 79.7 | 6.3 |  |  | 1069.4 | 89.1 | 7.7 |
|  |  |  | 2042 | 85.1 | 8.6 |  |  | 2096.1 | 87.3 | 8.3 |
| 25 | Ethylbenzene |  | 64.2 | 85.6 | 8.8 | 35 | 1,3,5-trimethyl benzene | 78.4 | 104.5 | 6.9 |
|  |  |  | 140.0 | 93.3 | 11.1 |  |  | 147.5 | 98.3 | 9.8 |
|  |  |  | 255.9 | 85.3 | 7.1 |  |  | 253.3 | 84.4 | 9.8 |
|  |  |  | 592.7 | 98.8 | 4.8 |  |  | 563.9 | 94.0 | 7.8 |
|  |  |  | 1057.2 | 88.1 | 3.5 |  |  | 1050.2 | 87.5 | 6.6 |
|  |  |  | 2041.4 | 85.1 | 4.7 |  |  | 2099.6 | 87.5 | 9.0 |
| 26 | *m,p-*xylene |  | 62.7 | 83.6 | 9.2 | 36 | 4-chloro toluene | 74.0 | 98.7 | 9.3 |
|  |  |  | 119.4 | 79.6 | 11.6 |  |  | 142.2 | 94.8 | 11.8 |
|  |  |  | 280.1 | 93.4 | 7.6 |  |  | 257.9 | 86.0 | 7.7 |
|  |  |  | 589.1 | 98.2 | 4.3 |  |  | 540.5 | 90.1 | 5.3 |
|  |  |  | 948.7 | 79.1 | 6.0 |  |  | 952.9 | 79.4 | 3.8 |
|  |  |  | 1988.5 | 82.9 | 4.7 |  |  | 1972.0 | 82.2 | 5.4 |
| 27 | *o*-xylene |  | 60.7 | 80.9 | 6.3 | 37 | *Tert*-butyl benzene | 77.1 | 102.8 | 10.3 |
|  |  |  | 128.9 | 85.9 | 8.6 |  |  | 161.3 | 107.5 | 12.4 |
|  |  |  | 260.1 | 86.7 | 3.6 |  |  | 273.7 | 91.2 | 8.3 |
|  |  |  | 562.3 | 93.7 | 5.2 |  |  | 587.7 | 98.0 | 4.7 |
|  |  |  | 972.3 | 81.0 | 6.8 |  |  | 1016.4 | 84.7 | 6.7 |
|  |  |  | 1924.1 | 80.2 | 6.9 |  |  | 2115.3 | 88.1 | 5.4 |
| 28 | Styren |  | 60.7 | 80.9 | 6.9 | 38 | 1,2,4-trimethyl benzene | 67.1 | 89.5 | 6.9 |
|  |  |  | 119.8 | 79.9 | 4.3 |  |  | 137.6 | 91.7 | 9.3 |
|  |  |  | 266.9 | 89.0 | 10.1 |  |  | 322.2 | 107.4 | 4.4 |
|  |  |  | 494.8 | 82.5 | 8.7 |  |  | 568.7 | 94.8 | 5.6 |
|  |  |  | 1087.4 | 90.6 | 7.3 |  |  | 975.4 | 81.3 | 7.2 |
|  |  |  | 2037.1 | 84.9 | 9.9 |  |  | 2156.8 | 89.9 | 6.9 |
| 29 | Tribromo methane |  | 63.0 | 84.0 | 3.6 | 39 | *Sec*-butyl benzene | 78.6 | 104.8 | 7.6 |
|  |  |  | 146.5 | 97.7 | 5.3 |  |  | 134.8 | 89.9 | 4.4 |
|  |  |  | 279.4 | 93.1 | 6.6 |  |  | 276.1 | 92.0 | 11.1 |
|  |  |  | 569.9 | 95.0 | 9.1 |  |  | 590.0 | 98.3 | 9.8 |
|  |  |  | 1016.1 | 84.7 | 10.2 |  |  | 1064.3 | 88.7 | 8.0 |
|  |  |  | 2150.6 | 89.6 | 4.3 |  |  | 2154.2 | 89.8 | 10.9 |
| 30 | Isopropyl benzene |  | 81.1 | 108.1 | 4.7 | 40 | 1,3-dichloro benzene | 67.6 | 90.1 | 4.3 |
|  |  |  | 142.7 | 95.1 | 3.4 |  |  | 141.5 | 94.3 | 5.6 |
|  |  |  | 270.7 | 90.2 | 4.6 |  |  | 266.5 | 88.8 | 7.2 |
|  |  |  | 574.5 | 95.8 | 6.3 |  |  | 529.7 | 88.3 | 9.9 |
|  |  |  | 956.7 | 79.7 | 8.6 |  |  | 906.6 | 75.6 | 9.9 |
|  |  |  | 2170.2 | 90.4 | 6.3 |  |  | 2129.2 | 88.7 | 6.7 |

| **S.6.** Continuous. | | | | | | | | | | |
| --- | --- | --- | --- | --- | --- | --- | --- | --- | --- | --- |
| **No** | **VOCs** | **Spike (µg/L)** | **R_Mean_ (µg/L)** | **R_Mean_**  **(%)** | **STD (%)** | **No** | **VOCs** | **R_Mean_**  **(µg/L)** | **R_Mean_ (%)** | **STD (%)** |
| 41 | 4-isopropyl tolune | 75.0  150.0  300.0  600.0  1200.0  2400.0 | 62.1 | 82.8 | 7.9 | 45 | 1,2-dibromo-3-chloropropane | ND | ND | - |
|  |  |  | 156.3 | 104.2 | 6.6 |  |  | 141.3 | 94.2 | 3.4 |
|  |  |  | 288.9 | 96.3 | 9.0 |  |  | 265.3 | 88.4 | 9.7 |
|  |  |  | 594.1 | 99.0 | 9.4 |  |  | 567.7 | 94.6 | 8.5 |
|  |  |  | 1004.1 | 83.7 | 12.2 |  |  | 1044.2 | 87.0 | 6.9 |
|  |  |  | 2130.8 | 88.8 | 7.9 |  |  | 2104.8 | 87.7 | 9.5 |
| 42 | 1,4-dichloro benzene |  | 74.9 | 99.9 | 11.6 | 46 | 1,2,4-trichloro benzene | 65.7 | 87.6 | 11.2 |
|  |  |  | 133.1 | 88.7 | 10.5 |  |  | 121.5 | 81.0 | 7.6 |
|  |  |  | 261.2 | 87.1 | 7.0 |  |  | 302.2 | 100.7 | 8.3 |
|  |  |  | 499.1 | 83.2 | 7.7 |  |  | 485.8 | 81.0 | 6.9 |
|  |  |  | 988.6 | 82.4 | 6.5 |  |  | 1198.3 | 99.8 | 9.4 |
|  |  |  | 2048.9 | 85.4 | 8.8 |  |  | 2057.9 | 85.7 | 7.0 |
| 43 | *n*-butylbenzene |  | 69.2 | 92.3 | 6.9 | 47 | Hexachloro-1,3-buthadiene | 65.3 | 87.1 | 8.0 |
|  |  |  | 149.5 | 99.7 | 5.8 |  |  | 151.1 | 100.7 | 6.8 |
|  |  |  | 269.6 | 89.9 | 7.8 |  |  | 266.6 | 88.9 | 9.0 |
|  |  |  | 583.7 | 97.3 | 8.0 |  |  | 567.1 | 94.5 | 9.2 |
|  |  |  | 943.2 | 78.6 | 10.2 |  |  | 999.2 | 83.3 | 12.5 |
|  |  |  | 1942.1 | 80.9 | 6.2 |  |  | 2000.4 | 83.4 | 7.6 |
| 44 | 1,2-dichloro benzene |  | 75.1 | 100.1 | 6.2 | 48 | 1,2,3-trichloro benzene | 62.3 | 83.1 | 5.3 |
|  |  |  | 142.6 | 95.1 | 8.6 |  |  | 144.5 | 96.3 | 3.7 |
|  |  |  | 252.2 | 84.0 | 6.6 |  |  | 323.6 | 107.9 | 5.2 |
|  |  |  | 510.8 | 85.1 | 3.7 |  |  | 604.9 | 100.8 | 6.7 |
|  |  |  | 1132.9 | 94.4 | 5.5 |  |  | 937.3 | 78.1 | 7.7 |
|  |  |  | 2106.5 | 87.8 | 8.8 |  |  | 2053.9 | 85.6 | 8.2 |

| **S.7.**  The graphical demonstration of the graph obtained by plotting the mean recovery (R_Mean_) values against spike levels, and the slope represents the mean recovery of the method (R_Mean-L_). | |
| --- | --- |
|  |  |
|  |  |
|  |  |
|  |  |
|  |  |

| **S.7.**  Continuous.. | |
| --- | --- |
|  |  |
|  |  |
|  |  |
|  |  |
|  |  |

| **S.8.** To compare AC and CAC for VOC spike concentrations of 150 and 2400 µg/L. Averages of Recoveries (**R_Mean_**), Percents of Recoveries (**R_Mean_** %), Percent of Standard Deviations (STD %) in CS_2_ desorption process | | | | | | | | | | | | | |
| --- | --- | --- | --- | --- | --- | --- | --- | --- | --- | --- | --- | --- | --- |
| **No** | **VOCs** |  | **AC** | | | | |  | **CAC** | | | | |
|  |  |  | **R_Mean_**  **( µg/L )** |  | **R_Mean_ (%)** |  | **STD (%)** |  | **R_Mean_**  **( µg/L )** |  | **R_Mean_  (%)** |  | **STD (%)** |
| 1 | Fluorotrichloro methane |  | 129.9 |  | 86.6 |  | 10.5 |  | 125.3 |  | 83.5 |  | 10.8 |
|  |  |  | 2061.6 |  | 85.9 |  | 9.0 |  | 2006.8 |  | 83.6 |  | 8.2 |
| 2 | 1,1-dichloroethene |  | 133.2 |  | 88.8 |  | 11.9 |  | 121.4 |  | 80.9 |  | 8.7 |
|  |  |  | 2112.0 |  | 88.0 |  | 4.8 |  | 2033.9 |  | 84.7 |  | 8.3 |
| 3 | Trans-1,2-ichloroethene |  | 126.2 |  | 84.1 |  | 8.6 |  | 121.4 |  | 80.9 |  | 8.7 |
|  |  |  | 2088.0 |  | 87.0 |  | 8.1 |  | 2033.9 |  | 84.7 |  | 8.8 |
| 4 | 1,2-dichloroethane |  | 148.7 |  | 99.1 |  | 3.4 |  | 141.3 |  | 94.2 |  | 4.4 |
|  |  |  | 2172.0 |  | 90.5 |  | 9.2 |  | 2118.1 |  | 88.3 |  | 9.4 |
| 5 | Cis-1,2-dichloro ethene |  | 160.1 |  | 106.7 |  | 7.8 |  | 155.7 |  | 103.8 |  | 6.8 |
|  |  |  | 2292.0 |  | 95.5 |  | 8.1 |  | 2241.2 |  | 93.4 |  | 8.2 |
| 6 | Bromochloro methane |  | 155.7 |  | 103.8 |  | 4.9 |  | 148.4 |  | 98.9 |  | 6.9 |
|  |  |  | 1980.0 |  | 82.5 |  | 8.2 |  | 1923.9 |  | 80.2 |  | 8.4 |
| 7 | Trichlro methane |  | 157.4 |  | 104.9 |  | 7.0 |  | 153.0 |  | 102.0 |  | 7.6 |
|  |  |  | 2174.4 |  | 90.6 |  | 8.9 |  | 2122.2 |  | 88.4 |  | 9.1 |
| 8 | 1,1,1-trichloro ethane |  | 158.0 |  | 105.3 |  | 6.2 |  | 150.6 |  | 100.4 |  | 7.2 |
|  |  |  | 2296.8 |  | 95.7 |  | 7.1 |  | 2246.3 |  | 93.6 |  | 7.5 |
| 9 | 1,1-dichloro-1-propene |  | 138.3 |  | 92.2 |  | 3.3 |  | 133.7 |  | 89.1 |  | 5.3 |
|  |  |  | 2064.0 |  | 86.0 |  | 6.1 |  | 2008.2 |  | 83.7 |  | 6.0 |
| 10 | Benzene |  | 155.0 |  | 103.3 |  | 10.9 |  | 147.6 |  | 98.4 |  | 10.3 |
|  |  |  | 2088.0 |  | 87.0 |  | 10.6 |  | 2034.2 |  | 84.8 |  | 10.2 |
| 11 | Tetrachloro methane |  | 146.3 |  | 97.5 |  | 7.2 |  | 143.2 |  | 95.5 |  | 7.6 |
|  |  |  | 2236.8 |  | 93.2 |  | 6.5 |  | 2185.8 |  | 91.1 |  | 6.8 |
| 12 | Trichloro ethene |  | 149.4 |  | 99.6 |  | 6.2 |  | 143.5 |  | 95.7 |  | 6.2 |
|  |  |  | 2047.2 |  | 85.3 |  | 6.6 |  | 1992,7 |  | 83,0 |  | 6.8 |
| 13 | 1,2-dichloro propane |  | 161.0 |  | 107.3 |  | 3.3 |  | 153.6 |  | 102.4 |  | 4.3 |
|  |  |  | 2179.2 |  | 90.8 |  | 7.4 |  | 2084.8 |  | 86.9 |  | 7.1 |
| 14 | Dibromo methane |  | 151.2 |  | 100.8 |  | 8.4 |  | 146.8 |  | 97.9 |  | 8.7 |
|  |  |  | 2109.6 |  | 87.9 |  | 8.0 |  | 2056.4 |  | 85.7 |  | 7.1 |
| 15 | Bromodichloro methane |  | 156.8 |  | 104.5 |  | 10.5 |  | 149.4 |  | 99.6 |  | 10.1 |
|  |  |  | 2181.6 |  | 90.9 |  | 4.2 |  | 2130 |  | 88.8 |  | 4.7 |
| 16 | 1,3-dichloro propene (cis+trans) |  | 147.0 |  | 98.0 |  | 10.2 |  | 141.1 |  | 94.1 |  | 10.6 |
|  |  |  | 2167.2 |  | 90.3 |  | 4.4 |  | 2052.5 |  | 85.5 |  | 4.6 |
| 17 | Toluen |  | 163.8 |  | 109.2 |  | 7.5 |  | 161.1 |  | 107.4 |  | 7.0 |
|  |  |  | 2191.2 |  | 91.3 |  | 8.1 |  | 2077.6 |  | 86.6 |  | 8.6 |
| 18 | 1,1,2-trichloro ethane |  | 145.1 |  | 96.7 |  | 3.5 |  | 141.9 |  | 94.6 |  | 3.6 |
|  |  |  | 2032.8 |  | 84.7 |  | 8.8 |  | 1957.1 |  | 81.5 |  | 7.9 |
| 19 | Tetrachloro ethene |  | 140.7 |  | 93.8 |  | 4.6 |  | 137.5 |  | 91.7 |  | 4.9 |
|  |  |  | 2016.0 |  | 84.0 |  | 3.5 |  | 1958.7 |  | 81.6 |  | 3.2 |
| 20 | 1,3-dichloro propane |  | 149.1 |  | 99.4 |  | 7.8 |  | 143.3 |  | 95.5 |  | 7.5 |
|  |  |  | 2179.2 |  | 90.8 |  | 6.2 |  | 2085.8 |  | 86.9 |  | 6.6 |
| 21 | Dibromochloromethane |  | 175.1 |  | 116.7 |  | 6.8 |  | 170.9 |  | 113.9 |  | 6.7 |
|  |  |  | 2133.6 |  | 88.9 |  | 6.4 |  | 2080.0 |  | 86.7 |  | 5.3 |
| 22 | 1,2-dibromoethane |  | 178.1 |  | 118.7 |  | 6.1 |  | 170.6 |  | 113.7 |  | 9.9 |
|  |  |  | 2112.0 |  | 88.0 |  | 6.5 |  | 2058.8 |  | 85.8 |  | 7.4 |
| 23 | Chlorobenzene |  | 140.7 |  | 93.8 |  | 3.2 |  | 136.1 |  | 90.7 |  | 3.6 |
|  |  |  | 2102.4 |  | 87.6 |  | 7.9 |  | 2047.5 |  | 85.3 |  | 7.3 |
| 24 | 1,1,1,2-tetrachloroethane |  | 176.1 |  | 117.4 |  | 9.3 |  | 170.6 |  | 113.7 |  | 8.2 |
|  |  |  | 2076.0 |  | 86.5 |  | 8.6 |  | 2042.0 |  | 85.1 |  | 10.5 |
| 25 | Ethylbenzene |  | 144.5 |  | 96.3 |  | 11.1 |  | 140.0 |  | 93.3 |  | 10.4 |
|  |  |  | 2095.2 |  | 87.3 |  | 4.7 |  | 2041.4 |  | 85.1 |  | 4.2 |
| 26 | p,m-xylene |  | 126.5 |  | 84.3 |  | 11.6 |  | 119.4 |  | 79.6 |  | 11.0 |
|  |  |  | 2044.8 |  | 85.2 |  | 4.7 |  | 1988.5 |  | 82.9 |  | 4.9 |

| **S.8.** Continuous.. | | | | | | | | | | | | | |
| --- | --- | --- | --- | --- | --- | --- | --- | --- | --- | --- | --- | --- | --- |
| **No** | **VOCs** |  | **AC** | | | | |  | **CAC** | | | | |
|  |  |  | **R_Mean_**  **( µg/L )** |  | **R_Mean_ (%)** |  | **STD (%)** |  | **R_Mean_**  **( µg/L )** |  | **R_Mean_  (%)** |  | **STD (%)** |
| 27 | o-xylene |  | 133.5 |  | 89.0 |  | 8.6 |  | 128.9 |  | 85.9 |  | 8.4 |
|  |  |  | 1980.0 |  | 82.5 |  | 6.9 |  | 1924.1 |  | 80.2 |  | 6.8 |
| 28 | Styren |  | 126.9 |  | 84.6 |  | 4.3 |  | 119.8 |  | 89.9 |  | 4.1 |
|  |  |  | 2092.8 |  | 87.2 |  | 9.9 |  | 2037.1 |  | 84.9 |  | 9.8 |
| 29 | Tribromo methane |  | 150.9 |  | 100.6 |  | 5.3 |  | 146.5 |  | 97.7 |  | 5.2 |
|  |  |  | 2203.2 |  | 91.8 |  | 4.3 |  | 2150.6 |  | 89.6 |  | 4.3 |
| 30 | Isopropyl benzene |  | 150.0 |  | 100.0 |  | 3.4 |  | 142.7 |  | 95.1 |  | 3.3 |
|  |  |  | 2200.8 |  | 91.7 |  | 6.3 |  | 2149.2 |  | 89.6 |  | 6.2 |
| 31 | 1,1,2,2-tetrachloro ethane |  | 146.7 |  | 97.8 |  | 3.3 |  | 143.7 |  | 95.8 |  | 3.3 |
|  |  |  | 2222.4 |  | 92.6 |  | 6.3 |  | 2170.9 |  | 90.5 |  | 6.2 |
| 32 | Bromobenzene |  | 161.9 |  | 107.9 |  | 6.5 |  | 155.9 |  | 103.9 |  | 6.3 |
|  |  |  | 2128.8 |  | 88.7 |  | 6.8 |  | 2075.4 |  | 86.5 |  | 6.7 |
| 33 | n-propyl benzene |  | 153.9 |  | 102.6 |  | 11.4 |  | 146.4 |  | 97.6 |  | 5.6 |
|  |  |  | 1999.2 |  | 83.3 |  | 4.7 |  | 1993.4 |  | 83.1 |  | 6.0 |
| 34 | 2-chloro toluene |  | 150.0 |  | 100.0 |  | 3.8 |  | 145.6 |  | 97.1 |  | 3.7 |
|  |  |  | 2150.4 |  | 89.6 |  | 8.3 |  | 2096.1 |  | 87.3 |  | 8.2 |
| 35 | 1,3,5-trimethyl benzene |  | 154.8 |  | 103.2 |  | 9.8 |  | 147.5 |  | 98.3 |  | 9.4 |
|  |  |  | 2152.8 |  | 89.7 |  | 9.0 |  | 2099.6 |  | 93.5 |  | 8.9 |
| 36 | 4-chloro toluene |  | 148.1 |  | 98.7 |  | 11.8 |  | 142.2 |  | 94.8 |  | 11.5 |
|  |  |  | 2085.6 |  | 86.9 |  | 5.4 |  | 1972.0 |  | 82.2 |  | 5.2 |
| 37 | tert-butyl benzene |  | 164.0 |  | 109.3 |  | 12.4 |  | 161.3 |  | 107.5 |  | 12.3 |
|  |  |  | 2157.6 |  | 89.9 |  | 5.4 |  | 2115.3 |  | 88.1 |  | 5.2 |
| 38 | 1,2,4-trimethyl benzene |  | 140.9 |  | 93.9 |  | 9.3 |  | 137.6 |  | 91.7 |  | 9.2 |
|  |  |  | 2229.6 |  | 92.9 |  | 6.9 |  | 2156.8 |  | 87.9 |  | 6.8 |
| 39 | sec-butyl benzene |  | 138.0 |  | 92.0 |  | 4.4 |  | 134.8 |  | 89.0 |  | 4.4 |
|  |  |  | 2205.6 |  | 91.9 |  | 10.9 |  | 2154.2 |  | 89.8 |  | 10.8 |
| 40 | 1,3-dichloro benzene |  | 147.3 |  | 98.2 |  | 5.6 |  | 141.5 |  | 94.3 |  | 5.4 |
|  |  |  | 2222.4 |  | 92.6 |  | 6.7 |  | 2129.2 |  | 88.7 |  | 6.5 |
| 41 | 4-isopropyl tolune |  | 160.7 |  | 107.1 |  | 6.6 |  | 156.3 |  | 104.2 |  | 6.5 |
|  |  |  | 2184.0 |  | 91.0 |  | 7.9 |  | 2130.8 |  | 88.8 |  | 7.8 |
| 42 | 1,4-dichloro benzene |  | 140.3 |  | 93.5 |  | 10.5 |  | 133.1 |  | 88.7 |  | 10.1 |
|  |  |  | 2102.4 |  | 87.6 |  | 8.8 |  | 2048.9 |  | 85.4 |  | 8.7 |
| 43 | n-butylbenzene |  | 153.8 |  | 102.5 |  | 5.8 |  | 149.5 |  | 99.7 |  | 11.2 |
|  |  |  | 2088.0 |  | 87.0 |  | 6.2 |  | 1942.1 |  | 80.9 |  | 4.7 |
| 44 | 1,2-dichloro benzene |  | 149.9 |  | 99.9 |  | 8.6 |  | 142.6 |  | 95.1 |  | 8.3 |
|  |  |  | 2160.0 |  | 90.0 |  | 8.8 |  | 2106.5 |  | 87.8 |  | 8.7 |
| 45 | 1,2-dibromo-3-chloro propane |  | 144.5 |  | 96.3 |  | 3.4 |  | 141.3 |  | 94.2 |  | 3.4 |
|  |  |  | 2157.6 |  | 89.9 |  | 9.5 |  | 2104.8 |  | 88.7 |  | 9.4 |
| 46 | 1,2,4-trichloro benzene |  | 127.4 |  | 84.9 |  | 7.6 |  | 121.5 |  | 81.0 |  | 7.4 |
|  |  |  | 2112.0 |  | 88.0 |  | 7.0 |  | 2057.9 |  | 85.7 |  | 6.9 |
| 47 | Hexachloro-1,3-butha diene |  | 158.4 |  | 105.6 |  | 6.8 |  | 151.1 |  | 100.7 |  | 6.5 |
|  |  |  | 2095.2 |  | 87.3 |  | 7.6 |  | 2000.4 |  | 83.4 |  | 7.4 |
| 48 | 1,2,3-trichloro benzene |  | 149.0 |  | 99.3 |  | 3.7 |  | 144.5 |  | 96.3 |  | 3.6 |
|  |  |  | 2107.2 |  | 87.8 |  | 8.2 |  | 2053.9 |  | 85.6 |  | 8.1 |

| **S.9.** Repeatability results, spike concentrations, recovery values ​, Analyst numbers, Relative standard deviation RSDr, combined relative standard deviation (RSD_WR(pool)_) and VOCs | | | | | | | | | | | | |  |
| --- | --- | --- | --- | --- | --- | --- | --- | --- | --- | --- | --- | --- | --- |
| **No** |  | **VOCs** |  | **Recovery number** |  | ***Spike Concentrations (100 µg/L and 1000 µg/L)*** | | | | | | | |
|  |  |  |  |  |  | ***Analyst I*** | |  | ***Analyst II*** | |  | ***Analyst III*** | |
| 1 |  | Fluorotrichloromethane |  | Rec1 |  | 86.12 | 870.24 |  | 85.26 | 905.05 |  | 89.56 | 861.54 |
|  |  |  |  | Rec2 |  | 87.02 | 925.10 |  | 85.28 | 934.35 |  | 87.89 | 906.60 |
|  |  |  |  | Rec3 |  | 85.13 | 996.40 |  | 86.84 | 976.47 |  | 83.43 | 1016.33 |
|  |  |  |  | Rec4 |  | 80.05 | 902.60 |  | 80.85 | 893.57 |  | 79.25 | 911.63 |
|  |  |  |  | Rec5 |  | 81.09 | 1040.00 |  | 79.47 | 1050.40 |  | 81.90 | 1019.20 |
|  |  |  |  | **Mean** |  | **83.88** | **946.87** |  | **83.54** | **951.97** |  | **84.41** | **943.06** |
|  |  |  |  | **SD** |  | **3.12** | **69.70** |  | **3.19** | **63.66** |  | **4.26** | **70.94** |
|  |  |  |  | **RSDr (%)** |  | **0.04** | **0.07** |  | **0.04** | **0.07** |  | **0.05** | **0.08** |
|  |  |  |  | **RSD_WR(pool)_** | | **0.059** | | | | | | | |
| 2 |  | 1,1-dichloroethene |  | Rec1 |  | 79.14 | 850.24 |  | 78.35 | 875.75 |  | 81.51 | 841.74 |
|  |  |  |  | Rec2 |  | 80.07 | 885.10 |  | 80.87 | 902.80 |  | 81.67 | 893.95 |
|  |  |  |  | Rec3 |  | 87.99 | 956.40 |  | 89.75 | 937.27 |  | 86.23 | 975.53 |
|  |  |  |  | Rec4 |  | 81.65 | 932.60 |  | 80.01 | 913.95 |  | 81.01 | 923.85 |
|  |  |  |  | Rec5 |  | 90.01 | 940.00 |  | 88.21 | 930.60 |  | 89.11 | 921.20 |
|  |  |  |  | **Mean** |  | **83.77** | **912.87** |  | **83.44** | **912.07** |  | **83.91** | **911.25** |
|  |  |  |  | **SD** |  | **4.91** | **43.91** |  | **5.17** | **24.43** |  | **3.59** | **48.79** |
|  |  |  |  | **RSDr (%)** |  | **0.06** | **0.05** |  | **0.06** | **0.03** |  | **0.04** | **0.050** |
|  |  |  |  | **RSD_WR(pool)_** | | **0.050** | | | | | | | |
| 3 |  | *Trans*-1,2-dichloroethene |  | Rec1 |  | 77.16 | 820.24 |  | 76.39 | 812.04 |  | 78.69 | 822.38 |
|  |  |  |  | Rec2 |  | 78.06 | 865.10 |  | 80.41 | 847.80 |  | 76.50 | 891.05 |
|  |  |  |  | Rec3 |  | 85.79 | 916.40 |  | 88.36 | 934.73 |  | 87.50 | 943.89 |
|  |  |  |  | Rec4 |  | 79.60 | 902.60 |  | 82.79 | 911.63 |  | 80.40 | 938.70 |
|  |  |  |  | Rec5 |  | 87.76 | 910.00 |  | 91.27 | 891.80 |  | 86.00 | 946.40 |
|  |  |  |  | **Mean** |  | **81.68** | **882.87** |  | **83.84** | **879.60** |  | **81.82** | **908.49** |
|  |  |  |  | **SD** |  | **4.79** | **40.27** |  | **6.00** | **49.47** |  | **4.74** | **53.20** |
|  |  |  |  | **RSDr (%)** |  | **0.06** | **0.05** |  | **0.07** | **0.06** |  | **0.06** | **0.06** |
|  |  |  |  | **RSD_WR(pool)_** | | **0.059** | | | | | | | |
| 4 |  | 1,2-dichloroethane |  | Rec1 |  | 86.74 | 886.40 |  | 85.00 | 895.26 |  | 87.60 | 868.67 |
|  |  |  |  | Rec2 |  | 87.79 | 942.60 |  | 88.67 | 923.75 |  | 86.04 | 952.03 |
|  |  |  |  | Rec3 |  | 96.48 | 980.10 |  | 95.51 | 970.30 |  | 94.13 | 950.99 |
|  |  |  |  | Rec4 |  | 89.52 | 952.87 |  | 91.31 | 962.40 |  | 90.42 | 971.93 |
|  |  |  |  | Rec5 |  | 98.70 | 930.00 |  | 101.66 | 948.60 |  | 100.67 | 957.90 |
|  |  |  |  | **Mean** |  | **91.85** | **938.39** |  | **92.43** | **940.06** |  | **91.77** | **940.30** |
|  |  |  |  | **SD** |  | **5.39** | **34.43** |  | **6.43** | **30.66** |  | **5.85** | **40.90** |
|  |  |  |  | **RSDr (%)** |  | **0.06** | **0.04** |  | **0.07** | **0.03** |  | **0.06** | **0.04** |
|  |  |  |  | **RSD_WR(pool)_** | | **0.053** | | | | | | | |
| 5 |  | *Cis*-1,2-dichloroethene |  | Rec1 |  | 92.72 | 946.40 |  | 96.43 | 984.26 |  | 94.94 | 974.56 |
|  |  |  |  | Rec2 |  | 93.85 | 1092.60 |  | 94.79 | 1070.75 |  | 91.97 | 1103.53 |
|  |  |  |  | Rec3 |  | 103.13 | 985.10 |  | 101.07 | 994.95 |  | 104.17 | 965.40 |
|  |  |  |  | Rec4 |  | 95.70 | 1017.87 |  | 94.74 | 1007.69 |  | 95.44 | 1017.89 |
|  |  |  |  | Rec5 |  | 105.51 | 1000.20 |  | 106.56 | 1020.20 |  | 107.62 | 1010.20 |
|  |  |  |  | **Mean** |  | **98.18** | **1008.43** |  | **98.72** | **1015.57** |  | **98.83** | **1014.32** |
|  |  |  |  | **SD** |  | **5.76** | **53.93** |  | **5.09** | **33.67** |  | **6.70** | **54.68** |
|  |  |  |  | **RSDr (%)** |  | **0.06** | **0.05** |  | **0.05** | **0.03** |  | **0.07** | **0.05** |
|  |  |  |  | **RSD_WR(pool)_** | | **0.054** | | | | | | | |

**S.9.** continuous..

| **No** |  | **VOCs** | |  | **Recovery number** |  | ***Spike Concentrations (100 µg/L and 1000 µg/L)*** | | | | | | | |
| --- | --- | --- | --- | --- | --- | --- | --- | --- | --- | --- | --- | --- | --- | --- |
|  |  |  |  |  |  |  | ***Analyst I*** | |  | ***Analyst II*** | |  | ***Analyst III*** | |
| 6 |  | | Bromochloromethane |  | Rec1 |  | 88.70 | 950.24 |  | 90.48 | 931.24 |  | 86.93 | 969.24 |
|  |  | |  |  | Rec2 |  | 98.16 | 985.10 |  | 96.20 | 994.95 |  | 99.14 | 965.40 |
|  |  | |  |  | Rec3 |  | 96.03 | 856.40 |  | 94.11 | 847.84 |  | 95.07 | 839.27 |
|  |  | |  |  | Rec4 |  | 89.66 | 922.60 |  | 88.76 | 941.05 |  | 91.45 | 913.37 |
|  |  | |  |  | Rec5 |  | 105.41 | 1040.00 |  | 108.58 | 1071.20 |  | 105.75 | 1053.20 |
|  |  | |  |  | **Mean** |  | **95.59** | **950.87** |  | **95.62** | **957.25** |  | **95.67** | **948.10** |
|  |  | |  |  | **SD** |  | **6.82** | **68.63** |  | **7.81** | **82.64** |  | **7.22** | **78.81** |
|  |  | |  |  | **RSDr (%)** |  | **0.07** | **0.07** |  | **0.08** | **0.09** |  | **0.08** | **0.08** |
|  |  | |  |  | **RSD_WR(pool)_** | | **0.079** | | | | | | | |
| 7 |  | | Trichlromethane |  | Rec1 |  | 86.93 | 876.40 |  | 89.54 | 885.16 |  | 87.80 | 902.69 |
|  |  | |  |  | Rec2 |  | 97.18 | 1022.60 |  | 99.13 | 1002.15 |  | 95.24 | 1043.05 |
|  |  | |  |  | Rec3 |  | 95.07 | 975.10 |  | 93.17 | 965.35 |  | 94.12 | 955.60 |
|  |  | |  |  | Rec4 |  | 105.79 | 947.87 |  | 103.68 | 957.35 |  | 106.85 | 928.91 |
|  |  | |  |  | Rec5 |  | 104.36 | 930.20 |  | 103.32 | 948.80 |  | 106.45 | 920.90 |
|  |  | |  |  | **Mean** |  | **97.87** | **950.43** |  | **97.77** | **951.76** |  | **98.09** | **950.23** |
|  |  | |  |  | **SD** |  | **7.63** | **54.11** |  | **6.25** | **42.42** |  | **8.31** | **55.27** |
|  |  | |  |  | **RSDr (%)** |  | **0.08** | **0.06** |  | **0.06** | **0.04** |  | **0.08** | **0.06** |
|  |  | |  |  | **RSD_WR(pool)_** | | **0.066** | | | | | | | |
| 8 |  | | 1,1,1-trichloroethane |  | Rec1 |  | 91.62 | 950.24 |  | 94.37 | 931.24 |  | 89.79 | 978.75 |
|  |  | |  |  | Rec2 |  | 104.02 | 965.10 |  | 107.14 | 974.75 |  | 105.06 | 994.05 |
|  |  | |  |  | Rec3 |  | 95.13 | 1066.40 |  | 98.94 | 1055.74 |  | 94.18 | 1109.06 |
|  |  | |  |  | Rec4 |  | 95.05 | 1042.60 |  | 98.85 | 1063.45 |  | 96.95 | 1084.30 |
|  |  | |  |  | Rec5 |  | 91.09 | 890.00 |  | 89.27 | 916.70 |  | 93.82 | 872.20 |
|  |  | |  |  | **Mean** |  | **95.38** | **982.87** |  | **97.71** | **988.37** |  | **95.96** | **1007.67** |
|  |  | |  |  | **SD** |  | **5.18** | **71.68** |  | **6.60** | **68.49** |  | **5.69** | **94.24** |
|  |  | |  |  | **RSDr (%)** |  | **0.05** | **0.07** |  | **0.07** | **0.07** |  | **0.06** | **0.09** |
|  |  | |  |  | **RSD_WR(pool)_** | | **0.071** | | | | | | | |
| 9 |  | | 1,1-dichloro-1-propene |  | Rec1 |  | 78.70 | 890.24 |  | 79.49 | 872.44 |  | 77.13 | 899.14 |
|  |  | |  |  | Rec2 |  | 91.96 | 905.10 |  | 91.04 | 914.15 |  | 92.88 | 896.05 |
|  |  | |  |  | Rec3 |  | 95.03 | 1056.40 |  | 96.93 | 1045.84 |  | 94.08 | 1077.53 |
|  |  | |  |  | Rec4 |  | 96.66 | 882.60 |  | 99.56 | 900.25 |  | 98.59 | 909.08 |
|  |  | |  |  | Rec5 |  | 100.41 | 949.90 |  | 104.43 | 978.40 |  | 103.43 | 987.90 |
|  |  | |  |  | **Mean** |  | **92.55** | **936.85** |  | **94.29** | **942.21** |  | **93.22** | **953.94** |
|  |  | |  |  | **SD** |  | **8.32** | **71.74** |  | **9.58** | **69.76** |  | **9.91** | **78.73** |
|  |  | |  |  | **RSDr (%)** |  | **0.09** | **0.08** |  | **0.10** | **0.07** |  | **0.11** | **0.08** |
|  |  | |  |  | **RSD_WR(pool)_** | | **0.089** | | | | | | | |
| 10 |  | | Benzene |  | Rec1 |  | 83.70 | 1046.40 |  | 82.03 | 1088.26 |  | 87.05 | 1025.47 |
|  |  | |  |  | Rec2 |  | 86.96 | 982.60 |  | 85.22 | 962.95 |  | 86.21 | 971.48 |
|  |  | |  |  | Rec3 |  | 105.03 | 1085.12 |  | 103.98 | 1095.97 |  | 106.08 | 1074.27 |
|  |  | |  |  | Rec4 |  | 86.66 | 922.87 |  | 89.26 | 913.64 |  | 85.79 | 950.55 |
|  |  | |  |  | Rec5 |  | 110.91 | 867.87 |  | 114.24 | 885.23 |  | 113.13 | 893.90 |
|  |  | |  |  | **Mean** |  | **94.65** | **980.97** |  | **94.95** | **989.21** |  | **95.65** | **983.14** |
|  |  | |  |  | **SD** |  | **12.40** | **88.46** |  | **13.67** | **98.01** |  | **12.99** | **69.39** |
|  |  | |  |  | **RSDr (%)** |  | **0.13** | **0.09** |  | **0.14** | **0.10** |  | **0.14** | **0.07** |
|  |  | |  |  | **RSD_WR(pool)_** | | **0.115** | | | | | | | |

| **S.9.** continuous.. | | | | | | | | | | | | | |
| --- | --- | --- | --- | --- | --- | --- | --- | --- | --- | --- | --- | --- | --- |
| **No** |  | **VOCs** |  | **Recovery number** |  | ***Spike Concentrations (100 µg/L and 1000 µg/L)*** | | | | | | | |
|  |  |  |  |  |  | ***Analyst I*** | |  | ***Analyst II*** | |  | ***Analyst III*** | |
| 11 |  | Tetrachloro methane |  | Rec1 |  | 77.72 | 906.40 |  | 78.50 | 888.27 |  | 76.17 | 915.46 |
|  |  |  |  | Rec2 |  | 83.85 | 1122.60 |  | 82.17 | 1133.83 |  | 84.69 | 1100.15 |
|  |  |  |  | Rec3 |  | 93.02 | 989.79 |  | 92.09 | 979.89 |  | 91.94 | 959.14 |
|  |  |  |  | Rec4 |  | 97.50 | 932.87 |  | 98.48 | 951.53 |  | 99.45 | 942.20 |
|  |  |  |  | Rec5 |  | 99.71 | 877.87 |  | 101.70 | 904.20 |  | 102.70 | 895.43 |
|  |  |  |  | **Mean** |  | **90.36** | **965.90** |  | **90.59** | **971.54** |  | **90.99** | **962.47** |
|  |  |  |  | **SD** |  | **9.32** | **96.83** |  | **10.06** | **97.82** |  | **10.83** | **80.75** |
|  |  |  |  | **RSDr (%)** |  | **10.32** | **10.03** |  | **11.11** | **10.07** |  | **11.90** | **8.39** |
|  |  |  |  | **RSD_WR(pool)_** |  | **0.104** | | | | | | | |
| 12 |  | Trichloro ethene |  | Rec1 |  | 88.70 | 926.40 |  | 87.82 | 907.87 |  | 86.93 | 917.14 |
|  |  |  |  | Rec2 |  | 91.96 | 1142.60 |  | 90.12 | 1154.03 |  | 92.88 | 1119.75 |
|  |  |  |  | Rec3 |  | 110.03 | 1009.79 |  | 112.23 | 999.69 |  | 108.93 | 1029.98 |
|  |  |  |  | Rec4 |  | 91.66 | 952.87 |  | 92.57 | 971.93 |  | 93.49 | 962.40 |
|  |  |  |  | Rec5 |  | 114.91 | 947.87 |  | 112.61 | 976.30 |  | 118.36 | 928.91 |
|  |  |  |  | **Mean** |  | **99.45** | **995.90** |  | **99.07** | **1001.96** |  | **100.12** | **991.63** |
|  |  |  |  | **SD** |  | **12.08** | **87.61** |  | **12.30** | **91.57** |  | **13.04** | **84.00** |
|  |  |  |  | **RSDr (%)** |  | **12.14** | **8.80** |  | **12.42** | **9.14** |  | **13.03** | **8.47** |
|  |  |  |  | **RSD_WR(pool)_** |  | **0.108** | | | | | | | |
| 13 |  | 1.2-dichloro propane |  | Rec1 |  | 108.70 | 876.40 |  | 107.61 | 885.16 |  | 109.78 | 867.64 |
|  |  |  |  | Rec2 |  | 97.55 | 1092.60 |  | 98.52 | 1070.75 |  | 95.59 | 1103.53 |
|  |  |  |  | Rec3 |  | 94.14 | 959.79 |  | 96.02 | 950.19 |  | 93.20 | 978.98 |
|  |  |  |  | Rec4 |  | 97.07 | 902.87 |  | 95.12 | 911.90 |  | 98.04 | 884.81 |
|  |  |  |  | Rec5 |  | 104.99 | 897.87 |  | 102.89 | 915.83 |  | 107.09 | 879.91 |
|  |  |  |  | **Mean** |  | **100.49** | **945.90** |  | **100.03** | **946.76** |  | **100.74** | **942.97** |
|  |  |  |  | **SD** |  | **6.09** | **87.61** |  | **5.20** | **73.06** |  | **7.29** | **100.14** |
|  |  |  |  | **RSDr (%)** |  | **6.06** | **9.26** |  | **5.19** | **7.72** |  | **7.24** | **10.62** |
|  |  |  |  | **RSD_WR(pool)_** |  | **0.079** | | | | | | | |
| 14 |  | Dibromo methane |  | Rec1 |  | 100.70 | 942.60 |  | 104.72 | 952.03 |  | 101.70 | 980.30 |
|  |  |  |  | Rec2 |  | 94.05 | 909.79 |  | 92.16 | 891.59 |  | 90.92 | 864.90 |
|  |  |  |  | Rec3 |  | 92.14 | 952.87 |  | 93.06 | 943.34 |  | 91.22 | 962.40 |
|  |  |  |  | Rec4 |  | 95.07 | 997.87 |  | 94.11 | 1007.85 |  | 96.02 | 987.89 |
|  |  |  |  | Rec5 |  | 102.99 | 845.90 |  | 105.05 | 862.82 |  | 103.05 | 869.22 |
|  |  |  |  | **Mean** |  | **96.99** | **929.81** |  | **97.82** | **931.52** |  | **96.58** | **938.94** |
|  |  |  |  | **SD** |  | **4.63** | **56.50** |  | **6.49** | **56.35** |  | **5.68** | **53.52** |
|  |  |  |  | **RSDr (%)** |  | **4.77** | **6.08** |  | **6.63** | **6.05** |  | **5.88** | **5.70** |
|  |  |  |  | **RSD_WR(pool)_** |  | **0.059** | | | | | | | |
| 15 |  | Bromodichloromethane |  | Rec1 |  | 82.16 | 956.40 |  | 84.63 | 965.96 |  | 82.98 | 985.09 |
|  |  |  |  | Rec2 |  | 90.06 | 902.60 |  | 93.67 | 920.65 |  | 91.86 | 938.70 |
|  |  |  |  | Rec3 |  | 100.79 | 885.10 |  | 101.80 | 867.40 |  | 98.77 | 893.95 |
|  |  |  |  | Rec4 |  | 98.60 | 897.87 |  | 96.63 | 879.91 |  | 97.06 | 867.11 |
|  |  |  |  | Rec5 |  | 97.76 | 980.20 |  | 96.78 | 970.40 |  | 94.82 | 981.98 |
|  |  |  |  | **Mean** |  | **93.88** | **924.43** |  | **94.70** | **920.86** |  | **93.10** | **933.37** |
|  |  |  |  | **SD** |  | **7.70** | **41.42** |  | **6.34** | **47.50** |  | **6.22** | **52.47** |
|  |  |  |  | **RSDr (%)** |  | **8.20** | **4.48** |  | **6.70** | **5.16** |  | **6.68** | **5.62** |
|  |  |  |  | **RSD_WR(pool)_** |  | **0.063** | | | | | | | |

| **S.9.** continuous.. | | | | | | | | | | | | | |
| --- | --- | --- | --- | --- | --- | --- | --- | --- | --- | --- | --- | --- | --- |
| **No** |  | **VOCs** |  | **Recovery number** |  | ***Spike Concentrations (100 µg/L and 1000 µg/L)*** | | | | | | | |
|  |  |  |  |  |  | ***Analyst I*** | |  | ***Analyst II*** | |  | ***Analyst III*** | |
| 16 |  | 1.3-dichloro propene (cis+trans) |  | Rec1 |  | 87.14 | 926.40 |  | 85.40 | 935.66 |  | 88.01 | 907.87 |
|  |  |  |  | Rec2 |  | 87.72 | 872.60 |  | 89.48 | 855.15 |  | 85.97 | 890.05 |
|  |  |  |  | Rec3 |  | 91.85 | 975.10 |  | 92.77 | 965.35 |  | 90.93 | 984.85 |
|  |  |  |  | Rec4 |  | 97.02 | 987.87 |  | 95.08 | 997.75 |  | 97.99 | 968.11 |
|  |  |  |  | Rec5 |  | 93.50 | 880.20 |  | 92.57 | 897.80 |  | 95.37 | 871.40 |
|  |  |  |  | **Mean** |  | **91.45** | **928.43** |  | **91.06** | **930.34** |  | **91.65** | **924.46** |
|  |  |  |  | **SD** |  | **4.12** | **52.82** |  | **3.74** | **55.92** |  | **5.00** | **49.57** |
|  |  |  |  | **RSDr (%)** |  | **4.51** | **5.69** |  | **4.11** | **6.01** |  | **5.46** | **5.36** |
|  |  |  |  | **RSD_WR(pool)_** |  | **0.052** | | | | | | | |
| 17 |  | Toluen |  | Rec1 |  | 92.14 | 1102.60 |  | 93.06 | 1146.70 |  | 95.83 | 1113.63 |
|  |  |  |  | Rec2 |  | 93.87 | 969.79 |  | 95.74 | 979.48 |  | 94.80 | 989.18 |
|  |  |  |  | Rec3 |  | 105.99 | 1112.87 |  | 103.87 | 1090.61 |  | 104.67 | 1062.11 |
|  |  |  |  | Rec4 |  | 98.65 | 936.40 |  | 96.67 | 927.04 |  | 97.66 | 917.67 |
|  |  |  |  | Rec5 |  | 109.01 | 922.60 |  | 107.92 | 931.83 |  | 110.10 | 913.37 |
|  |  |  |  | **Mean** |  | **99.93** | **1008.85** |  | **99.45** | **1015.13** |  | **100.61** | **999.19** |
|  |  |  |  | **SD** |  | **7.39** | **91.95** |  | **6.20** | **98.72** |  | **6.56** | **88.27** |
|  |  |  |  | **RSDr (%)** |  | **7.39** | **9.11** |  | **6.23** | **9.72** |  | **6.52** | **8.83** |
|  |  |  |  | **RSD_WR(pool)_** |  | **0.081** | | | | | | | |
| 18 |  | 1.1.2-trichloro ethane |  | Rec1 |  | 83.22 | 860.24 |  | 85.72 | 886.05 |  | 86.19 | 888.47 |
|  |  |  |  | Rec2 |  | 88.85 | 845.10 |  | 91.51 | 862.00 |  | 90.63 | 870.45 |
|  |  |  |  | Rec3 |  | 99.02 | 866.40 |  | 102.98 | 849.07 |  | 97.04 | 901.06 |
|  |  |  |  | Rec4 |  | 105.50 | 1022.60 |  | 109.72 | 1002.15 |  | 103.39 | 1063.50 |
|  |  |  |  | Rec5 |  | 107.71 | 809.90 |  | 105.55 | 801.80 |  | 106.63 | 793.70 |
|  |  |  |  | **Mean** |  | **96.86** | **880.85** |  | **99.10** | **880.21** |  | **96.78** | **903.44** |
|  |  |  |  | **SD** |  | **10.57** | **82.22** |  | **10.08** | **74.75** |  | **8.53** | **98.72** |
|  |  |  |  | **RSDr (%)** |  | **10.92** | **9.33** |  | **10.17** | **8.49** |  | **8.81** | **10.93** |
|  |  |  |  | **RSD_WR(pool)_** |  | **0.098** | | | | | | | |
| 19 |  | Tetrachloro ethene |  | Rec1 |  | 73.22 | 870.24 |  | 73.95 | 861.54 |  | 72.49 | 878.94 |
|  |  |  |  | Rec2 |  | 83.85 | 855.10 |  | 83.01 | 838.00 |  | 82.17 | 846.55 |
|  |  |  |  | Rec3 |  | 89.02 | 876.40 |  | 90.80 | 893.93 |  | 91.78 | 890.28 |
|  |  |  |  | Rec4 |  | 90.50 | 1032.60 |  | 93.22 | 1042.93 |  | 91.41 | 1063.58 |
|  |  |  |  | Rec5 |  | 99.91 | 819.90 |  | 103.90 | 803.50 |  | 97.91 | 852.70 |
|  |  |  |  | **Mean** |  | **87.30** | **890.85** |  | **88.98** | **887.98** |  | **87.15** | **906.41** |
|  |  |  |  | **SD** |  | **9.78** | **82.22** |  | **11.24** | **92.71** |  | **9.94** | **89.70** |
|  |  |  |  | **RSDr (%)** |  | **11.20** | **9.23** |  | **12.63** | **10.44** |  | **11.40** | **9.90** |
|  |  |  |  | **RSD_WR(pool)_** |  | **0.109** | | | | | | | |
| 20 |  | 1.3-dichloro propane |  | Rec1 |  | 87.02 | 920.24 |  | 87.89 | 929.44 |  | 88.19 | 932.42 |
|  |  |  |  | Rec2 |  | 88.50 | 905.10 |  | 86.73 | 887.00 |  | 85.00 | 869.26 |
|  |  |  |  | Rec3 |  | 102.71 | 926.40 |  | 101.68 | 917.14 |  | 100.66 | 935.48 |
|  |  |  |  | Rec4 |  | 85.70 | 1082.60 |  | 86.56 | 1093.43 |  | 87.43 | 1104.36 |
|  |  |  |  | Rec5 |  | 90.76 | 969.90 |  | 92.58 | 989.30 |  | 94.43 | 969.51 |
|  |  |  |  | **Mean** |  | **90.94** | **960.85** |  | **91.09** | **963.26** |  | **91.14** | **962.21** |
|  |  |  |  | **SD** |  | **6.84** | **72.20** |  | **6.40** | **81.71** |  | **6.36** | **87.32** |
|  |  |  |  | **RSDr (%)** |  | **7.52** | **7.51** |  | **7.03** | **8.48** |  | **6.98** | **9.08** |
|  |  |  |  | **RSD_WR(pool)_** |  | **0.078** | | | | | | | |

| **S.9.** continuous.. | | | | | | | | | | | | | |
| --- | --- | --- | --- | --- | --- | --- | --- | --- | --- | --- | --- | --- | --- |
| **No** |  | **VOCs** |  | **Recovery number** |  | ***Spike Concentrations (100 µg/L and 1000 µg/L)*** | | | | | | | |
|  |  |  |  |  |  | ***Analyst I*** | |  | ***Analyst II*** | |  | ***Analyst III*** | |
| 21 |  | Dibromochloromethane |  | Rec1 |  | 92.16 | 916.40 |  | 90.32 | 953.06 |  | 95.85 | 898.07 |
|  |  |  |  | Rec2 |  | 100.06 | 892.60 |  | 98.06 | 874.75 |  | 98.06 | 874.75 |
|  |  |  |  | Rec3 |  | 110.79 | 999.79 |  | 109.68 | 1009.78 |  | 111.90 | 989.79 |
|  |  |  |  | Rec4 |  | 108.60 | 972.87 |  | 111.86 | 963.14 |  | 107.52 | 1002.05 |
|  |  |  |  | Rec5 |  | 107.76 | 937.87 |  | 110.99 | 956.63 |  | 109.91 | 966.00 |
|  |  |  |  | **Mean** |  | **103.88** | **943.90** |  | **104.18** | **951.47** |  | **104.65** | **946.13** |
|  |  |  |  | **SD** |  | **7.70** | **42.97** |  | **9.56** | **48.61** |  | **7.23** | **56.64** |
|  |  |  |  | **RSDr (%)** |  | **7.41** | **4.55** |  | **9.17** | **5.11** |  | **6.91** | **5.99** |
|  |  |  |  | **RSD_WR(pool)_** |  | **0.067** | | | | | | | |
| 22 |  | *1.2-dibromoethane* |  | Rec1 |  | 91.22 | 992.60 |  | 93.05 | 972.75 |  | 94.91 | 1011.16 |
|  |  |  |  | Rec2 |  | 98.85 | 909.79 |  | 96.87 | 918.88 |  | 94.93 | 900.51 |
|  |  |  |  | Rec3 |  | 110.02 | 1102.87 |  | 107.82 | 1091.84 |  | 105.67 | 1102.67 |
|  |  |  |  | Rec4 |  | 118.50 | 897.87 |  | 117.32 | 915.83 |  | 116.14 | 906.67 |
|  |  |  |  | Rec5 |  | 125.71 | 945.90 |  | 129.48 | 974.28 |  | 133.36 | 993.77 |
|  |  |  |  | **Mean** |  | **108.86** | **969.81** |  | **108.91** | **974.72** |  | **109.00** | **983.07** |
|  |  |  |  | **SD** |  | **14.05** | **83.03** |  | **14.93** | **71.25** |  | **16.22** | **83.53** |
|  |  |  |  | **RSDr (%)** |  | **12.90** | **8.56** |  | **13.71** | **7.31** |  | **14.88** | **8.50** |
|  |  |  |  | **RSD_WR(pool)_** |  | **0.114** | | | | | | | |
| 23 |  | *Chlorobenzene* |  | Rec1 |  | 77.16 | 900.24 |  | 79.48 | 909.24 |  | 77.93 | 927.25 |
|  |  |  |  | Rec2 |  | 83.06 | 935.10 |  | 86.39 | 916.40 |  | 81.40 | 972.50 |
|  |  |  |  | Rec3 |  | 93.79 | 806.40 |  | 97.54 | 798.34 |  | 92.85 | 838.66 |
|  |  |  |  | Rec4 |  | 88.60 | 872.60 |  | 86.83 | 881.33 |  | 89.49 | 855.15 |
|  |  |  |  | Rec5 |  | 93.76 | 989.90 |  | 94.70 | 1009.70 |  | 95.63 | 999.80 |
|  |  |  |  | **Mean** |  | **87.28** | **900.85** |  | **88.99** | **903.00** |  | **87.46** | **918.67** |
|  |  |  |  | **SD** |  | **7.18** | **68.60** |  | **7.20** | **75.87** |  | **7.54** | **70.69** |
|  |  |  |  | **RSDr (%)** |  | **8.23** | **7.61** |  | **8.10** | **8.40** |  | **8.62** | **7.70** |
|  |  |  |  | **RSD_WR(pool)_** |  | **0.081** | | | | | | | |
| 24 |  | 1.1.1.2-tetrachloroethane |  | Rec1 |  | 113.70 | 946.40 |  | 112.56 | 927.47 |  | 111.42 | 936.94 |
|  |  |  |  | Rec2 |  | 113.45 | 882.60 |  | 115.71 | 891.43 |  | 114.58 | 900.25 |
|  |  |  |  | Rec3 |  | 107.34 | 985.12 |  | 110.56 | 975.27 |  | 106.27 | 1014.68 |
|  |  |  |  | Rec4 |  | 109.07 | 822.87 |  | 113.43 | 839.33 |  | 111.25 | 855.78 |
|  |  |  |  | Rec5 |  | 118.99 | 857.87 |  | 120.18 | 883.60 |  | 122.56 | 866.45 |
|  |  |  |  | **Mean** |  | **112.51** | **898.97** |  | **114.49** | **903.42** |  | **113.21** | **914.82** |
|  |  |  |  | **SD** |  | **4.55** | **65.99** |  | **3.68** | **50.96** |  | **6.01** | **64.22** |
|  |  |  |  | **RSDr (%)** |  | **4.04** | **7.34** |  | **3.21** | **5.64** |  | **5.31** | **7.02** |
|  |  |  |  | **RSD_WR(pool)_** |  | **0.056** | | | | | | | |
| 25 |  | Ethylbenzene |  | Rec1 |  | 81.16 | 902.60 |  | 79.54 | 884.55 |  | 77.95 | 851.01 |
|  |  |  |  | Rec2 |  | 86.16 | 899.79 |  | 85.30 | 908.78 |  | 87.03 | 890.79 |
|  |  |  |  | Rec3 |  | 95.49 | 912.87 |  | 96.44 | 903.74 |  | 94.53 | 922.00 |
|  |  |  |  | Rec4 |  | 89.80 | 866.40 |  | 91.60 | 883.73 |  | 93.43 | 927.91 |
|  |  |  |  | Rec5 |  | 97.76 | 1092.60 |  | 95.80 | 1125.38 |  | 100.69 | 1070.75 |
|  |  |  |  | **Mean** |  | **90.08** | **934.85** |  | **89.74** | **941.24** |  | **90.73** | **933.99** |
|  |  |  |  | **SD** |  | **6.77** | **89.89** |  | **7.23** | **103.55** |  | **8.63** | **81.43** |
|  |  |  |  | **RSDr (%)** |  | **7.51** | **9.62** |  | **8.05** | **11.00** |  | **9.51** | **8.72** |
|  |  |  |  | **RSD_WR(pool)_** |  | **0.091** | | | | | | | |

| **S.9.** continuous.. | | | | | | | | | | | | | |
| --- | --- | --- | --- | --- | --- | --- | --- | --- | --- | --- | --- | --- | --- |
| **No** |  | **VOCs** |  | **Recovery number** |  | ***Spike Concentrations (100 µg/L and 1000 µg/L)*** | | | | | | | |
|  |  |  |  |  |  | ***Analyst I*** | |  | ***Analyst II*** | |  | ***Analyst III*** | |
| 26 |  | m.p-xylene |  | Rec1 |  | 74.16 | 892.60 |  | 72.68 | 928.30 |  | 77.13 | 874.75 |
|  |  |  |  | Rec2 |  | 78.16 | 859.79 |  | 77.38 | 842.59 |  | 76.60 | 851.19 |
|  |  |  |  | Rec3 |  | 88.49 | 902.87 |  | 91.14 | 911.90 |  | 89.37 | 929.95 |
|  |  |  |  | Rec4 |  | 81.80 | 947.87 |  | 84.26 | 938.39 |  | 80.99 | 976.30 |
|  |  |  |  | Rec5 |  | 89.76 | 800.90 |  | 88.86 | 816.92 |  | 91.55 | 792.90 |
|  |  |  |  | **Mean** |  | **82.48** | **880.81** |  | **82.86** | **887.62** |  | **83.13** | **885.02** |
|  |  |  |  | **SD** |  | **6.66** | **54.66** |  | **7.75** | **54.42** |  | **6.95** | **70.87** |
|  |  |  |  | **RSDr (%)** |  | **8.07** | **6.21** |  | **9.35** | **6.13** |  | **8.36** | **8.01** |
|  |  |  |  | **RSD_WR(pool)_** |  | **0.078** | | | | | | | |
| 27 |  | o-xylene |  | Rec1 |  | 75.06 | 882.60 |  | 75.81 | 864.95 |  | 73.56 | 891.43 |
|  |  |  |  | Rec2 |  | 79.16 | 849.79 |  | 77.58 | 858.28 |  | 79.96 | 832.79 |
|  |  |  |  | Rec3 |  | 89.59 | 892.87 |  | 88.69 | 883.94 |  | 87.80 | 857.42 |
|  |  |  |  | Rec4 |  | 83.60 | 937.87 |  | 84.44 | 956.63 |  | 85.28 | 947.25 |
|  |  |  |  | Rec5 |  | 93.76 | 795.90 |  | 95.63 | 819.78 |  | 96.57 | 811.82 |
|  |  |  |  | **Mean** |  | **84.24** | **871.81** |  | **84.43** | **876.72** |  | **84.63** | **868.14** |
|  |  |  |  | **SD** |  | **7.58** | **52.84** |  | **8.14** | **50.39** |  | **8.62** | **53.24** |
|  |  |  |  | **RSDr (%)** |  | **9.00** | **6.06** |  | **9.64** | **5.75** |  | **10.19** | **6.13** |
|  |  |  |  | **RSD_WR(pool)_** |  | **0.080** | | | | | | | |
| 28 |  | Styren |  | Rec1 |  | 73.64 | 922.60 |  | 72.17 | 904.15 |  | 72.17 | 904.15 |
|  |  |  |  | Rec2 |  | 75.17 | 809.79 |  | 76.67 | 817.88 |  | 75.92 | 825.98 |
|  |  |  |  | Rec3 |  | 85.99 | 882.87 |  | 86.85 | 874.04 |  | 85.13 | 891.70 |
|  |  |  |  | Rec4 |  | 81.65 | 947.87 |  | 80.01 | 966.83 |  | 83.28 | 928.91 |
|  |  |  |  | Rec5 |  | 89.01 | 805.90 |  | 88.12 | 830.08 |  | 91.68 | 797.85 |
|  |  |  |  | **Mean** |  | **81.09** | **873.81** |  | **80.76** | **878.60** |  | **81.63** | **869.72** |
|  |  |  |  | **SD** |  | **6.66** | **64.53** |  | **6.75** | **60.20** |  | **7.72** | **55.34** |
|  |  |  |  | **RSDr (%)** |  | **8.22** | **7.39** |  | **8.36** | **6.85** |  | **9.45** | **6.36** |
|  |  |  |  | **RSD_WR(pool)_** |  | **0.078** | | | | | | | |
| 29 |  | *Tribromomethane* |  | Rec1 |  | 79.16 | 882.60 |  | 79.95 | 891.43 |  | 80.75 | 900.34 |
|  |  |  |  | Rec2 |  | 88.16 | 909.79 |  | 89.93 | 891.59 |  | 86.40 | 927.98 |
|  |  |  |  | Rec3 |  | 98.49 | 812.87 |  | 96.52 | 804.74 |  | 97.50 | 796.61 |
|  |  |  |  | Rec4 |  | 90.80 | 836.40 |  | 88.99 | 844.76 |  | 91.71 | 819.67 |
|  |  |  |  | Rec5 |  | 99.76 | 1102.60 |  | 98.76 | 1124.65 |  | 101.75 | 1091.57 |
|  |  |  |  | **Mean** |  | **91.28** | **908.85** |  | **90.83** | **911.43** |  | **91.62** | **907.24** |
|  |  |  |  | **SD** |  | **8.38** | **114.77** |  | **7.38** | **124.58** |  | **8.41** | **116.88** |
|  |  |  |  | **RSDr (%)** |  | **9.18** | **12.63** |  | **8.12** | **13.67** |  | **9.17** | **12.85** |
|  |  |  |  | **RSD_WR(pool)_** |  | **0.111** | | | | | | | |
| 30 |  | Isopropylbenzene |  | Rec1 |  | 106.70 | 892.60 |  | 109.90 | 928.30 |  | 110.96 | 919.38 |
|  |  |  |  | Rec2 |  | 106.45 | 829.79 |  | 110.70 | 813.19 |  | 104.32 | 862.98 |
|  |  |  |  | Rec3 |  | 96.34 | 902.87 |  | 100.19 | 911.90 |  | 97.30 | 938.98 |
|  |  |  |  | Rec4 |  | 98.07 | 947.87 |  | 96.10 | 938.39 |  | 97.08 | 928.91 |
|  |  |  |  | Rec5 |  | 106.99 | 805.90 |  | 108.06 | 822.02 |  | 109.13 | 813.96 |
|  |  |  |  | **Mean** |  | **102.91** | **875.81** |  | **104.99** | **882.76** |  | **103.76** | **892.84** |
|  |  |  |  | **SD** |  | **5.25** | **57.47** |  | **6.48** | **60.31** |  | **6.47** | **53.03** |
|  |  |  |  | **RSDr (%)** |  | **5.10** | **6.56** |  | **6.17** | **6.83** |  | **6.23** | **5.94** |
|  |  |  |  | **RSD_WR(pool)_** |  | **0.062** | | | | | | | |

| **S.9.** continuous.. | | | | | | | | | | | | | |
| --- | --- | --- | --- | --- | --- | --- | --- | --- | --- | --- | --- | --- | --- |
| **No** |  | **VOCs** |  | **Recovery number** |  | ***Spike Concentrations (100 µg/L and 1000 µg/L)*** | | | | | | | |
|  |  |  |  |  |  | ***Analyst I*** | |  | ***Analyst II*** | |  | ***Analyst III*** | |
| 31 |  | 1.1.2.2-tetrachloroethane |  | Rec1 |  | 83.64 | 937.87 |  | 81.97 | 919.11 |  | 80.33 | 900.73 |
|  |  |  |  | Rec2 |  | 91.17 | 795.90 |  | 90.25 | 803.86 |  | 92.08 | 787.95 |
|  |  |  |  | Rec3 |  | 99.99 | 865.81 |  | 100.99 | 857.15 |  | 98.99 | 874.46 |
|  |  |  |  | Rec4 |  | 97.65 | 892.60 |  | 99.60 | 910.45 |  | 101.59 | 928.66 |
|  |  |  |  | Rec5 |  | 104.01 | 972.60 |  | 101.93 | 1001.78 |  | 107.13 | 953.15 |
|  |  |  |  | **Mean** |  | **95.29** | **892.96** |  | **94.95** | **898.47** |  | **96.02** | **888.98** |
|  |  |  |  | **SD** |  | **8.01** | **68.04** |  | **8.62** | **73.99** |  | **10.31** | **63.73** |
|  |  |  |  | **RSDr (%)** |  | **8.40** | **7.62** |  | **9.08** | **8.24** |  | **10.74** | **7.17** |
|  |  |  |  | **RSD_WR(pool)_** |  | **0.086** | | | | | | | |
| 32 |  | Bromobenzene |  | Rec1 |  | 93.16 | 1082.60 |  | 91.30 | 1060.95 |  | 89.47 | 1039.73 |
|  |  |  |  | Rec2 |  | 97.16 | 879.79 |  | 96.19 | 888.58 |  | 98.14 | 870.99 |
|  |  |  |  | Rec3 |  | 112.49 | 912.87 |  | 115.86 | 903.74 |  | 111.36 | 940.25 |
|  |  |  |  | Rec4 |  | 105.80 | 866.40 |  | 108.98 | 883.73 |  | 107.92 | 892.39 |
|  |  |  |  | Rec5 |  | 114.76 | 832.60 |  | 117.05 | 857.58 |  | 118.20 | 849.25 |
|  |  |  |  | **Mean** |  | **104.68** | **914.85** |  | **105.88** | **918.92** |  | **105.02** | **918.52** |
|  |  |  |  | **SD** |  | **9.39** | **98.09** |  | **11.63** | **81.12** |  | **11.31** | **75.67** |
|  |  |  |  | **RSDr (%)** |  | **8.97** | **10.72** |  | **10.98** | **8.83** |  | **10.77** | **8.24** |
|  |  |  |  | **RSD_WR(pool)_** |  | **0.098** | | | | | | | |
| 33 |  | *n-propylbenzene* |  | Rec1 |  | 81.16 | 927.87 |  | 80.35 | 937.15 |  | 81.97 | 918.59 |
|  |  |  |  | Rec2 |  | 87.16 | 785.90 |  | 88.91 | 770.19 |  | 85.42 | 801.62 |
|  |  |  |  | Rec3 |  | 99.49 | 855.81 |  | 102.47 | 847.25 |  | 98.49 | 881.48 |
|  |  |  |  | Rec4 |  | 92.80 | 882.60 |  | 96.52 | 891.43 |  | 93.73 | 917.90 |
|  |  |  |  | Rec5 |  | 101.76 | 962.60 |  | 102.78 | 981.85 |  | 103.79 | 972.23 |
|  |  |  |  | **Mean** |  | **92.48** | **882.96** |  | **94.20** | **885.57** |  | **92.68** | **898.36** |
|  |  |  |  | **SD** |  | **8.54** | **68.04** |  | **9.58** | **81.77** |  | **9.02** | **63.03** |
|  |  |  |  | **RSDr (%)** |  | **9.23** | **7.71** |  | **10.17** | **9.23** |  | **9.74** | **7.02** |
|  |  |  |  | **RSD_WR(pool)_** |  | **0.089** | | | | | | | |
| 34 |  | 2-chlorotoluene |  | Rec1 |  | 83.16 | 896.40 |  | 81.50 | 914.33 |  | 84.82 | 878.47 |
|  |  |  |  | Rec2 |  | 87.16 | 832.60 |  | 85.42 | 815.95 |  | 83.71 | 799.63 |
|  |  |  |  | Rec3 |  | 100.49 | 1035.12 |  | 99.48 | 1014.42 |  | 98.48 | 1024.77 |
|  |  |  |  | Rec4 |  | 93.80 | 912.87 |  | 96.62 | 903.74 |  | 92.87 | 940.25 |
|  |  |  |  | Rec5 |  | 104.76 | 807.87 |  | 107.90 | 832.10 |  | 111.14 | 857.07 |
|  |  |  |  | **Mean** |  | **93.88** | **896.97** |  | **94.18** | **896.11** |  | **94.20** | **900.04** |
|  |  |  |  | **SD** |  | **8.97** | **88.63** |  | **10.72** | **78.91** |  | **11.24** | **85.98** |
|  |  |  |  | **RSDr (%)** |  | **9.56** | **9.88** |  | **11.39** | **8.81** |  | **11.93** | **9.55** |
|  |  |  |  | **RSD_WR(pool)_** |  | **0.102** | | | | | | | |
| 35 |  | *1.3.5-trimethylbenzene* |  | Rec1 |  | 110.80 | 891.40 |  | 115.24 | 918.14 |  | 114.13 | 927.06 |
|  |  |  |  | Rec2 |  | 117.76 | 827.60 |  | 122.47 | 860.70 |  | 127.37 | 895.13 |
|  |  |  |  | Rec3 |  | 109.68 | 1030.12 |  | 107.48 | 1071.33 |  | 114.06 | 1009.52 |
|  |  |  |  | Rec4 |  | 88.64 | 907.87 |  | 89.53 | 889.71 |  | 86.87 | 916.95 |
|  |  |  |  | Rec5 |  | 94.17 | 802.87 |  | 93.22 | 810.90 |  | 95.11 | 794.84 |
|  |  |  |  | **Mean** |  | **104.21** | **891.97** |  | **105.59** | **910.16** |  | **107.51** | **908.70** |
|  |  |  |  | **SD** |  | **12.25** | **88.63** |  | **14.08** | **98.43** |  | **16.29** | **77.00** |
|  |  |  |  | **RSDr (%)** |  | **11.76** | **9.94** |  | **13.33** | **10.81** |  | **15.15** | **8.47** |
|  |  |  |  | **RSD_WR(pool)_** |  | **0.118** | | | | | | | |

| **S.9.** continuous.. | | | | | | | | | | | | | |
| --- | --- | --- | --- | --- | --- | --- | --- | --- | --- | --- | --- | --- | --- |
| **No** |  | **VOCs** |  | **Recovery number** |  | ***Spike Concentrations (100 µg/L and 1000 µg/L)*** | | | | | | | |
|  |  |  |  |  |  | ***Analyst I*** | |  | ***Analyst II*** | |  | ***Analyst III*** | |
| 36 |  | 4-chlorotoluene |  | Rec1 |  | 82.06 | 842.60 |  | 83.70 | 834.17 |  | 81.24 | 859.45 |
|  |  |  |  | Rec2 |  | 91.16 | 809.79 |  | 93.90 | 825.98 |  | 92.99 | 834.08 |
|  |  |  |  | Rec3 |  | 104.59 | 792.87 |  | 108.77 | 816.65 |  | 107.72 | 824.58 |
|  |  |  |  | Rec4 |  | 103.60 | 801.40 |  | 104.64 | 833.46 |  | 107.75 | 809.41 |
|  |  |  |  | Rec5 |  | 103.76 | 1052.60 |  | 101.68 | 1063.13 |  | 104.80 | 1031.55 |
|  |  |  |  | **Mean** |  | **97.04** | **859.85** |  | **98.54** | **874.68** |  | **98.90** | **871.82** |
|  |  |  |  | **SD** |  | **10.05** | **109.38** |  | **9.92** | **105.58** |  | **11.59** | **91.13** |
|  |  |  |  | **RSDr (%)** |  | **10.36** | **12.72** |  | **10.06** | **12.07** |  | **11.72** | **10.45** |
|  |  |  |  | **RSD_WR(pool)_** |  | **0.113** | | | | | | | |
| 37 |  | Tert-butylbenzene |  | Rec1 |  | 95.16 | 902.60 |  | 94.21 | 884.55 |  | 93.26 | 893.57 |
|  |  |  |  | Rec2 |  | 98.16 | 999.79 |  | 99.15 | 989.79 |  | 97.18 | 1009.78 |
|  |  |  |  | Rec3 |  | 113.49 | 812.87 |  | 115.76 | 821.00 |  | 114.62 | 829.13 |
|  |  |  |  | Rec4 |  | 106.80 | 1016.40 |  | 104.67 | 1036.73 |  | 108.94 | 996.07 |
|  |  |  |  | Rec5 |  | 115.76 | 883.60 |  | 113.44 | 865.93 |  | 111.18 | 848.61 |
|  |  |  |  | **Mean** |  | **105.88** | **923.05** |  | **105.44** | **919.60** |  | **105.09** | **915.43** |
|  |  |  |  | **SD** |  | **9.09** | **84.73** |  | **9.18** | **90.11** |  | **9.24** | **83.36** |
|  |  |  |  | **RSDr (%)** |  | **8.59** | **9.18** |  | **8.70** | **9.80** |  | **8.84** | **9.11** |
|  |  |  |  | **RSD_WR(pool)_** |  | **0.090** | | | | | | | |
| 38 |  | 1.2.4-trimethylbenzene |  | Rec1 |  | 80.16 | 881.40 |  | 79.36 | 863.77 |  | 78.56 | 872.59 |
|  |  |  |  | Rec2 |  | 85.16 | 817.60 |  | 87.72 | 809.42 |  | 84.31 | 842.13 |
|  |  |  |  | Rec3 |  | 98.49 | 1020.12 |  | 101.44 | 1050.73 |  | 104.49 | 1082.25 |
|  |  |  |  | Rec4 |  | 96.80 | 897.87 |  | 97.77 | 924.80 |  | 99.71 | 906.85 |
|  |  |  |  | Rec5 |  | 95.76 | 792.87 |  | 97.67 | 784.94 |  | 94.80 | 808.73 |
|  |  |  |  | **Mean** |  | **91.28** | **881.97** |  | **92.79** | **886.73** |  | **92.37** | **902.51** |
|  |  |  |  | **SD** |  | **8.12** | **88.63** |  | **9.08** | **106.31** |  | **10.75** | **106.84** |
|  |  |  |  | **RSDr (%)** |  | **8.89** | **10.05** |  | **9.78** | **11.99** |  | **11.64** | **11.87** |
|  |  |  |  | **RSD_WR(pool)_** |  | **0.108** | | | | | | | |
| 39 |  | *Sec-butylbenzene* |  | Rec1 |  | 84.64 | 892.60 |  | 82.95 | 901.53 |  | 85.49 | 874.75 |
|  |  |  |  | Rec2 |  | 91.17 | 889.79 |  | 89.34 | 871.99 |  | 87.55 | 854.55 |
|  |  |  |  | Rec3 |  | 104.99 | 902.87 |  | 103.94 | 893.84 |  | 103.94 | 893.84 |
|  |  |  |  | Rec4 |  | 102.65 | 856.40 |  | 105.72 | 864.96 |  | 103.67 | 882.09 |
|  |  |  |  | Rec5 |  | 107.01 | 1082.60 |  | 110.22 | 1104.25 |  | 109.15 | 1115.08 |
|  |  |  |  | **Mean** |  | **98.09** | **924.85** |  | **98.43** | **927.31** |  | **97.96** | **924.06** |
|  |  |  |  | **SD** |  | **9.70** | **89.89** |  | **11.67** | **100.05** |  | **10.69** | **107.73** |
|  |  |  |  | **RSDr (%)** |  | **9.89** | **9.72** |  | **11.86** | **10.79** |  | **10.92** | **11.66** |
|  |  |  |  | **RSD_WR(pool)_** |  | **0.108** | | | | | | | |
| 40 |  | 1.3-dichlorobenzene |  | Rec1 |  | 83.16 | 812.60 |  | 84.82 | 796.35 |  | 81.50 | 828.85 |
|  |  |  |  | Rec2 |  | 88.16 | 799.79 |  | 90.81 | 815.78 |  | 89.93 | 823.78 |
|  |  |  |  | Rec3 |  | 98.49 | 802.87 |  | 102.43 | 810.90 |  | 99.47 | 834.98 |
|  |  |  |  | Rec4 |  | 94.80 | 811.40 |  | 95.75 | 795.17 |  | 92.91 | 819.51 |
|  |  |  |  | Rec5 |  | 99.76 | 952.60 |  | 97.76 | 943.07 |  | 98.76 | 933.55 |
|  |  |  |  | **Mean** |  | **92.88** | **835.85** |  | **94.32** | **832.25** |  | **92.51** | **848.14** |
|  |  |  |  | **SD** |  | **7.06** | **65.49** |  | **6.75** | **62.60** |  | **7.34** | **48.10** |
|  |  |  |  | **RSDr (%)** |  | **7.60** | **7.84** |  | **7.15** | **7.52** |  | **7.94** | **5.67** |
|  |  |  |  | **RSD_WR(pool)_** |  | **0.073** | | | | | | | |

| **S.9.** continuous.. | | | | | | | | | | | | | |
| --- | --- | --- | --- | --- | --- | --- | --- | --- | --- | --- | --- | --- | --- |
| **No** |  | **VOCs** |  | **Recovery number** |  | ***Spike Concentrations (100 µg/L and 1000 µg/L)*** | | | | | | | |
|  |  |  |  |  |  | ***Analyst I*** | |  | ***Analyst II*** | |  | ***Analyst III*** | |
| 41 |  | 4-isopropyltolune |  | Rec1 |  | 88.16 | 892.60 |  | 87.28 | 910.45 |  | 89.92 | 883.67 |
|  |  |  |  | Rec2 |  | 87.16 | 989.79 |  | 88.04 | 969.99 |  | 85.42 | 999.68 |
|  |  |  |  | Rec3 |  | 105.49 | 802.87 |  | 107.60 | 786.81 |  | 103.38 | 818.93 |
|  |  |  |  | Rec4 |  | 91.80 | 1006.40 |  | 89.97 | 996.34 |  | 90.89 | 986.27 |
|  |  |  |  | Rec5 |  | 97.76 | 873.60 |  | 95.80 | 899.81 |  | 100.69 | 856.13 |
|  |  |  |  | **Mean** |  | **94.08** | **913.05** |  | **93.74** | **912.68** |  | **94.06** | **908.94** |
|  |  |  |  | **SD** |  | **7.61** | **84.73** |  | **8.44** | **81.08** |  | **7.63** | **80.23** |
|  |  |  |  | **RSDr (%)** |  | **8.09** | **9.28** |  | **9.00** | **8.88** |  | **8.11** | **8.83** |
|  |  |  |  | **RSD_WR(pool)_** |  | **0.087** | | | | | | | |
| 42 |  | 1.4-dichlorobenzene |  | Rec1 |  | 84.16 | 801.60 |  | 83.32 | 825.65 |  | 86.69 | 793.58 |
|  |  |  |  | Rec2 |  | 88.16 | 810.79 |  | 90.81 | 843.22 |  | 91.69 | 835.11 |
|  |  |  |  | Rec3 |  | 101.49 | 791.87 |  | 104.53 | 823.54 |  | 105.55 | 815.62 |
|  |  |  |  | Rec4 |  | 92.80 | 801.40 |  | 94.66 | 785.37 |  | 90.95 | 817.43 |
|  |  |  |  | Rec5 |  | 107.76 | 953.60 |  | 105.60 | 963.14 |  | 108.84 | 934.53 |
|  |  |  |  | **Mean** |  | **94.88** | **831.85** |  | **95.78** | **848.18** |  | **96.74** | **839.25** |
|  |  |  |  | **SD** |  | **9.67** | **68.39** |  | **9.41** | **67.64** |  | **9.80** | **55.26** |
|  |  |  |  | **RSDr (%)** |  | **10.19** | **8.22** |  | **9.83** | **7.97** |  | **10.13** | **6.58** |
|  |  |  |  | **RSD_WR(pool)_** |  | **0.089** | | | | | | | |
| 43 |  | n-butylbenzene |  | Rec1 |  | 100.80 | 871.40 |  | 98.79 | 862.69 |  | 99.80 | 853.97 |
|  |  |  |  | Rec2 |  | 102.76 | 807.60 |  | 101.73 | 823.75 |  | 104.81 | 799.52 |
|  |  |  |  | Rec3 |  | 94.68 | 1010.12 |  | 97.52 | 1040.43 |  | 100.44 | 1071.64 |
|  |  |  |  | Rec4 |  | 89.64 | 887.87 |  | 92.33 | 923.38 |  | 93.23 | 914.50 |
|  |  |  |  | Rec5 |  | 93.17 | 782.87 |  | 96.89 | 790.70 |  | 94.10 | 814.18 |
|  |  |  |  | **Mean** |  | **96.21** | **871.97** |  | **97.45** | **888.19** |  | **98.47** | **890.76** |
|  |  |  |  | **SD** |  | **5.45** | **88.63** |  | **3.42** | **98.39** |  | **4.81** | **110,48** |
|  |  |  |  | **RSDr (%)** |  | **5.66** | **10.16** |  | **3.50** | **11.08** |  | **4.88** | **12.40** |
|  |  |  |  | **RSD_WR(pool)_** |  | **0.086** | | | | | | | |
| 44 |  | 1.2-dichlorobenzene |  | Rec1 |  | 86.16 | 881.60 |  | 89.61 | 916.86 |  | 93.19 | 953.54 |
|  |  |  |  | Rec2 |  | 92.16 | 978.79 |  | 90.32 | 959.21 |  | 88.51 | 940.03 |
|  |  |  |  | Rec3 |  | 107.49 | 791.87 |  | 108.56 | 799.79 |  | 109.65 | 807.78 |
|  |  |  |  | Rec4 |  | 97.40 | 995.40 |  | 96.43 | 985.45 |  | 95.47 | 975.59 |
|  |  |  |  | Rec5 |  | 106.76 | 862.60 |  | 108.89 | 879.85 |  | 111.07 | 897.45 |
|  |  |  |  | **Mean** |  | **98.00** | **902.05** |  | **98.76** | **908.23** |  | **99.58** | **914.88** |
|  |  |  |  | **SD** |  | **9.24** | **84.73** |  | **9.48** | **72.80** |  | **10.17** | **66.31** |
|  |  |  |  | **RSDr (%)** |  | **9.43** | **9.39** |  | **9.60** | **8.02** |  | **10.21** | **7.25** |
|  |  |  |  | **RSD_WR(pool)_** |  | **0.090** | | | | | | | |
| 45 |  | 1.2-dibromo-3-chloropropane |  | Rec1 |  | 86.16 | 892.60 |  | 88.75 | 874.75 |  | 84.44 | 919.38 |
|  |  |  |  | Rec2 |  | 92.16 | 989.79 |  | 95.85 | 999.68 |  | 93.09 | 1029.38 |
|  |  |  |  | Rec3 |  | 104.49 | 802.87 |  | 105.53 | 794.84 |  | 103.44 | 810.90 |
|  |  |  |  | Rec4 |  | 97.80 | 1006.40 |  | 95.85 | 1026.53 |  | 99.76 | 986.27 |
|  |  |  |  | Rec5 |  | 103.76 | 873.60 |  | 102.72 | 899.81 |  | 106.87 | 864.86 |
|  |  |  |  | **Mean** |  | **96.88** | **913.05** |  | **97.74** | **919.12** |  | **97.52** | **922.16** |
|  |  |  |  | **SD** |  | **7.80** | **84.73** |  | **6.59** | **94.62** |  | **8.92** | **88.43** |
|  |  |  |  | **RSDr (%)** |  | **8.05** | **9.28** |  | **6.74** | **10.30** |  | **9.15** | **9.59** |
|  |  |  |  | **RSD_WR(pool)_** |  | **0.089** | | | | | | | |

| **S.9.** continuous.. | | | | | | | | | | | | | |
| --- | --- | --- | --- | --- | --- | --- | --- | --- | --- | --- | --- | --- | --- |
| **No** |  | **VOCs** |  | **Recovery number** |  | ***Spike Concentrations (100 µg/L and 1000 µg/L)*** | | | | | | | |
|  |  |  |  |  |  | ***Analyst I*** | |  | ***Analyst II*** | |  | ***Analyst III*** | |
| 46 |  | 1.2.4-trichlorobenzene |  | Rec1 |  | 75.16 | 917.87 |  | 74.41 | 899.51 |  | 73.66 | 908.69 |
|  |  |  |  | Rec2 |  | 78.16 | 805.90 |  | 80.51 | 813.96 |  | 78.95 | 830.08 |
|  |  |  |  | Rec3 |  | 93.49 | 845.81 |  | 96.29 | 837.35 |  | 92.55 | 871.18 |
|  |  |  |  | Rec4 |  | 86.80 | 872.60 |  | 88.54 | 890.05 |  | 90.31 | 907.85 |
|  |  |  |  | Rec5 |  | 91.76 | 1012.60 |  | 94.51 | 1042.98 |  | 97.35 | 1074.27 |
|  |  |  |  | **Mean** |  | **85.08** | **890.96** |  | **86.85** | **896.77** |  | **86.56** | **918.41** |
|  |  |  |  | **SD** |  | **8.13** | **79.26** |  | **9.30** | **89.19** |  | **9.88** | **92.91** |
|  |  |  |  | **RSDr (%)** |  | **9.56** | **8.90** |  | **10.70** | **9.95** |  | **11.42** | **10.12** |
|  |  |  |  | **RSD_WR(pool)_** |  | **0.101** | | | | | | | |
| 47 |  | Hexachloro-1.3-buthadiene |  | Rec1 |  | 85.80 | 906.87 |  | 89.24 | 915.94 |  | 86.66 | 943.14 |
|  |  |  |  | Rec2 |  | 112.76 | 816.90 |  | 113.89 | 800.57 |  | 110.50 | 825.07 |
|  |  |  |  | Rec3 |  | 99.68 | 834.81 |  | 97.68 | 826.46 |  | 98.68 | 818.11 |
|  |  |  |  | Rec4 |  | 84.64 | 861.60 |  | 83.79 | 870.22 |  | 85.49 | 852.98 |
|  |  |  |  | Rec5 |  | 84.17 | 1001.60 |  | 85.01 | 1021.63 |  | 85.85 | 1011.62 |
|  |  |  |  | **Mean** |  | **93.41** | **884.36** |  | **93.92** | **886.96** |  | **93.44** | **890.19** |
|  |  |  |  | **SD** |  | **12.59** | **73.78** |  | **12.42** | **87.15** |  | **11.02** | **84.21** |
|  |  |  |  | **RSDr (%)** |  | **13.48** | **8.34** |  | **13.22** | **9.83** |  | **11.79** | **9.46** |
|  |  |  |  | **RSD_WR(pool)_** |  | **0.112** | | | | | | | |
| 48 |  | 1.2.3-trichlorobenzene |  | Rec1 |  | 79.16 | 918.87 |  | 77.58 | 955.62 |  | 82.33 | 900.49 |
|  |  |  |  | Rec2 |  | 85.16 | 828.90 |  | 83.46 | 812.33 |  | 81.79 | 796.08 |
|  |  |  |  | Rec3 |  | 98.49 | 846.81 |  | 97.50 | 855.27 |  | 99.47 | 838.34 |
|  |  |  |  | Rec4 |  | 89.80 | 873.60 |  | 92.50 | 864.86 |  | 88.91 | 899.81 |
|  |  |  |  | Rec5 |  | 98.76 | 1013.60 |  | 100.73 | 1033.87 |  | 100.73 | 1033.87 |
|  |  |  |  | **Mean** |  | **90.28** | **896.36** |  | **90.35** | **904.39** |  | **90.65** | **893.72** |
|  |  |  |  | **SD** |  | **8.50** | **73.78** |  | **9.67** | **89.22** |  | **9.09** | **89.90** |
|  |  |  |  | **RSDr (%)** |  | **9.42** | **8.23** |  | **10.70** | **9.87** |  | **10.03** | **10.06** |
|  |  |  |  | **RSD_WR(pool)_** |  | **0.097** | | | | | | | |

| **S.10.** Reproducibility results, spike concentrations, recovery values ​, Analyst numbers, number of days, mean variance ( Sd^2^), variance of repeatability ( Sr^2^), variance of inter days ( S_L_^2^), relative standard deviation of reproducibility (RSD_WR_), combined relative standard deviation (RSD_WRpool_) and VOCs | | | | | | | | | | | | | | | | | |
| --- | --- | --- | --- | --- | --- | --- | --- | --- | --- | --- | --- | --- | --- | --- | --- | --- | --- |
| **No** | **VOCs** | ***Spike Conc. µg/L*** | **Analysts** | **Recoveries, µg/L** | | | | | **Intra Days Control** | | | **Sd^2^** | **Sr^2^** | **S_L_^2^** | **S_WR_** | **RSD_WR_** | **RSD_WRpool_** |
|  |  |  |  | **1st Day** | **2nd Day** | **3rd Day** | **4th Day** | **5th Day** | **Mean** | **SD** | **RSD %** |  |  |  |  |  |  |
| 1 | Fluorotrichloromethane | 100 | Analyst I | 86.12 | 91.23 | 89.78 | 81.24 | 83.79 | 86.43 | 4.13 | **4.78** | 32.79 | 13.33 | 6.49 | 4.45 | 0.0515 | **0.052** |
|  |  |  | Analyst II | 85.26 | 81.64 | 92.45 | 86.34 | 79.56 | 85.05 | 4.96 | **5.83** |  |  |  |  |  |  |
|  |  |  | Analyst III | 89.56 | 82.45 | 93.65 | 84.48 | 89.05 | 87.84 | 4.43 | **5.04** |  |  |  |  |  |  |
|  |  |  | Mean of Analysts | 86.98 | 85.11 | 91.96 | 84.02 | 84.13 | **Mean (general)** | | |  |  |  |  |  |  |
|  |  |  | SD of Analysts | 2.28 | 5.32 | 1.98 | 2.58 | 4.75 | **86.44** | | |  |  |  |  |  |  |
|  |  |  | **RSD of Analysts (%)** | **2.62** | **6.25** | **2.15** | **3.07** | **5.65** |  |  |  |  |  |  |  |  |  |
|  |  | 1000 | Analyst I | 870.24 | 910.37 | 845.97 | 958.67 | 824.15 | 881.88 | 53.54 | **6.07** | 1336.21 | 2761.49 | -475.09 | 47.82 | 0.0535 |  |
|  |  |  | Analyst II | 905.05 | 891.26 | 941.24 | 986.08 | 870.35 | 918.80 | 45.62 | **4.97** |  |  |  |  |  |  |
|  |  |  | Analyst III | 861.54 | 848.94 | 922.43 | 838.64 | 943.06 | 882.92 | 46.77 | **5.30** |  |  |  |  |  |  |
|  |  |  | Mean of Analysts | 878.94 | 883.52 | 903.21 | 927.80 | 879.19 | **Mean (general)** | | |  |  |  |  |  |  |
|  |  |  | SD of Analysts | 23.02 | 31.44 | 50.46 | 78.42 | 59.95 | **894.53** | | |  |  |  |  |  |  |
|  |  |  | **RSD of Analysts (%)** | **2.62** | **3.56** | **5.59** | **8.45** | **6.82** |  |  |  |  |  |  |  |  |  |
| 2 | 1.1-dichloroethene | 100 | Analyst I | 79.14 | 83.09 | 87.63 | 90.18 | 83.24 | 84.66 | 4.31 | **5.09** | 23.11 | 16.14 | 2.32 | 4.30 | 0.0510 | **0.049** |
|  |  |  | Analyst II | 78.35 | 91.31 | 84.16 | 82.91 | 81.2 | 83.59 | 4.84 | **5.79** |  |  |  |  |  |  |
|  |  |  | Analyst III | 81.51 | 80.37 | 89.97 | 81.39 | 88.67 | 84.38 | 4.55 | **5.39** |  |  |  |  |  |  |
|  |  |  | Mean of Analysts | 79.67 | 84.92 | 87.25 | 84.83 | 84.37 | **Mean (general)** | | |  |  |  |  |  |  |
|  |  |  | SD of Analysts | 1.65 | 5.70 | 2.92 | 4.70 | 3.86 | **84.21** | | |  |  |  |  |  |  |
|  |  |  | **RSD of Analysts (%)** | **2.07** | **6.71** | **3.35** | **5.54** | **4.58** |  |  |  |  |  |  |  |  |  |
|  |  | 1000 | Analyst I | 850.24 | 876.5 | 946.84 | 810.55 | 824.66 | 861.76 | 53.82 | **6.25** | 567.11 | 2279.13 | -570.67 | 41.33 | 0.0477 |  |
|  |  |  | Analyst II | 875.75 | 847.6 | 912.77 | 867.08 | 831.11 | 866.86 | 30.97 | **3.57** |  |  |  |  |  |  |
|  |  |  | Analyst III | 841.74 | 863.33 | 805.26 | 926.14 | 906.52 | 868.60 | 48.76 | **5.61** |  |  |  |  |  |  |
|  |  |  | Mean of Analysts | 855.91 | 862.48 | 888.29 | 867.92 | 854.10 | **Mean (general)** | | |  |  |  |  |  |  |
|  |  |  | SD of Analysts | 17.70 | 14.47 | 73.90 | 57.80 | 45.51 | **865.74** | | |  |  |  |  |  |  |
|  |  |  | **RSD of Analysts (%)** | **2.07** | **1.68** | **8.32** | **6.66** | **5.33** |  |  |  |  |  |  |  |  |  |
| 3 | Trans-1.2-dichloroethene | 100 | Analyst I | 77.16 | 86.75 | 85.9 | 95.05 | 90.12 | 87.00 | 6.57 | **7.55** | 58.39 | 20.25 | 12.71 | 5.74 | 0.0674 | **0.056** |
|  |  |  | Analyst II | 76.39 | 94.18 | 89.55 | 82.35 | 84.31 | 85.35 | 6.82 | **7.99** |  |  |  |  |  |  |
|  |  |  | Analyst III | 78.69 | 81.34 | 86.47 | 86.14 | 83.94 | 83.32 | 3.30 | **3.96** |  |  |  |  |  |  |
|  |  |  | Mean of Analysts | 77.41 | 87.42 | 87.31 | 87.85 | 86.12 | **Mean (general)** | | |  |  |  |  |  |  |
|  |  |  | SD of Analysts | 1.17 | 6.44 | 1.96 | 6.52 | 3.47 | **85.22** | | |  |  |  |  |  |  |
|  |  |  | **RSD of Analysts (%)** | **1.51** | **7.37** | **2.25** | **7.42** | **4.02** |  |  |  |  |  |  |  |  |  |
|  |  | 1000 | Analyst I | 820.24 | 924.08 | 871.32 | 856.87 | 843.17 | 863.14 | 38.92 | **4.51** | 443.02 | 1820.81 | -459.26 | 36.90 | 0.0428 |  |
|  |  |  | Analyst II | 847.94 | 812.04 | 912.56 | 931.2 | 876.22 | 875.99 | 48.12 | **5.49** |  |  |  |  |  |  |
|  |  |  | Analyst III | 870.12 | 822.38 | 822.44 | 841.31 | 869.1 | 845.07 | 23.70 | **2.80** |  |  |  |  |  |  |
|  |  |  | Mean of Analysts | 846.10 | 852.83 | 868.77 | 876.46 | 862.83 | **Mean (general)** | | |  |  |  |  |  |  |
|  |  |  | SD of Analysts | 24.99 | 61.92 | 45.11 | 48.04 | 17.39 | **861.40** | | |  |  |  |  |  |  |
|  |  |  | **RSD of Analysts (%)** | **2.95** | **7.26** | **5.19** | **5.48** | **2.02** |  |  |  |  |  |  |  |  |  |

| **S.10.** continuous… | | | | | | | | | | | | | | | | | |
| --- | --- | --- | --- | --- | --- | --- | --- | --- | --- | --- | --- | --- | --- | --- | --- | --- | --- |
| **No** | **VOCs** | ***Spike Conc. µg/L*** | **Analysts** | **Recoveries, µg/L** | | | | | **Intra Days Control** | | | **Sd^2^** | **Sr^2^** | **S_L_^2^** | **S_WR_** | **RSD_WR_** | **RSD_WRpool_** |
|  |  |  |  | **1st Day** | **2nd Day** | **3rd Day** | **4th Day** | **5th Day** | **Mean** | **SD** | **RSD %** |  |  |  |  |  |  |

| 4 | 1.2-dichloroethane | 100 | Analyst I | 86.74 | 91.26 | 81.08 | 93.14 | 82.43 | 86.93 | 5.29 | **6.08** | 35.65 | 12.01 | 7.88 | 4.46 | 0.0513 | **0.054** |
| --- | --- | --- | --- | --- | --- | --- | --- | --- | --- | --- | --- | --- | --- | --- | --- | --- | --- |
|  |  |  | Analyst II | 81.36 | 84.56 | 85.01 | 94.87 | 87.56 | 86.67 | 5.09 | **5.87** |  |  |  |  |  |  |
|  |  |  | Analyst III | 91.78 | 87.6 | 83.21 | 88.45 | 84.28 | 87.06 | 3.43 | **3.94** |  |  |  |  |  |  |
|  |  |  | Mean of Analysts | 86.63 | 87.81 | 83.10 | 92.15 | 84.76 | **Mean (general)** | | |  |  |  |  |  |  |
|  |  |  | SD of Analysts | 5.21 | 3.35 | 1.97 | 3.32 | 2.60 | **86.89** | | |  |  |  |  |  |  |
|  |  |  | **RSD of Analysts (%)** | **6.02** | **3.82** | **2.37** | **3.60** | **3.07** |  |  |  |  |  |  |  |  |  |
|  |  | 1000 | Analyst I | 886.40 | 956.41 | 824.89 | 916.09 | 920.74 | 900.91 | 49.23 | **5.46** | 504.70 | 3341.52 | -945.61 | 48.95 | 0.0559 |  |
|  |  |  | Analyst II | 815.61 | 831.7 | 895.26 | 812.46 | 854.07 | 841.82 | 34.12 | **4.05** |  |  |  |  |  |  |
|  |  |  | Analyst III | 926.44 | 868.67 | 954.77 | 853.64 | 822.73 | 885.25 | 54.11 | **6.11** |  |  |  |  |  |  |
|  |  |  | Mean of Analysts | 876.15 | 885.59 | 891.64 | 860.73 | 865.85 | **Mean (general)** | | |  |  |  |  |  |  |
|  |  |  | SD of Analysts | 56.12 | 64.05 | 65.02 | 52.18 | 50.06 | **875.99** | | |  |  |  |  |  |  |
|  |  |  | **RSD of Analysts (%)** | **6.41** | **7.23** | **7.29** | **6.06** | **5.78** |  |  |  |  |  |  |  |  |  |
| 5 | Cis-1.2-dichloroethene | 100 | Analyst I | 87.84 | 88.49 | 93.37 | 92.72 | 82.95 | 89.08 | 4.22 | **4.73** | 17.78 | 35.88 | -6.03 | 5.46 | 0.0620 | **0.062** |
|  |  |  | Analyst II | 80.72 | 80.60 | 96.43 | 85.67 | 82.41 | 85.17 | 6.62 | **7.77** |  |  |  |  |  |  |
|  |  |  | Analyst III | 87.76 | 92.23 | 82.12 | 94.09 | 94.94 | 90.23 | 5.32 | **5.89** |  |  |  |  |  |  |
|  |  |  | Mean of Analysts | 85.44 | 87.11 | 90.64 | 90.83 | 86.77 | **Mean (general)** | | |  |  |  |  |  |  |
|  |  |  | SD of Analysts | 4.09 | 5.94 | 7.54 | 4.52 | 7.09 | **88.16** | | |  |  |  |  |  |  |
|  |  |  | **RSD of Analysts (%)** | **4.78** | **6.82** | **8.31** | **4.97** | **8.17** |  |  |  |  |  |  |  |  |  |
|  |  | 1000 | Analyst I | 946.40 | 888.42 | 906.84 | 834.15 | 1010.23 | 917.21 | 65.84 | **7.18** | 2129.25 | 3702.30 | -524.35 | 56.37 | 0.0625 |  |
|  |  |  | Analyst II | 847.25 | 984.26 | 863.21 | 920.45 | 874.89 | 898.01 | 55.37 | **6.17** |  |  |  |  |  |  |
|  |  |  | Analyst III | 835.28 | 845.34 | 974.56 | 866.47 | 924.68 | 889.27 | 58.93 | **6.63** |  |  |  |  |  |  |
|  |  |  | Mean of Analysts | 876.31 | 906.01 | 914.87 | 873.69 | 936.60 | **Mean (general)** | | |  |  |  |  |  |  |
|  |  |  | SD of Analysts | 60.99 | 71.11 | 56.11 | 43.60 | 68.45 | **901.50** | | |  |  |  |  |  |  |
|  |  |  | **RSD of Analysts (%)** | **6.96** | **7.85** | **6.13** | **4.99** | **7.31** |  |  |  |  |  |  |  |  |  |
| 6 | Bromochloromethane | 100 | Analyst I | 88.70 | 84.24 | 96.29 | 87.55 | 90.12 | 89.38 | 4.43 | **4.96** | 1.86 | 31.62 | -9.92 | 4.66 | 0.0518 | **0.058** |
|  |  |  | Analyst II | 97.45 | 90.48 | 85.64 | 87.84 | 93.74 | 91.03 | 4.69 | **5.16** |  |  |  |  |  |  |
|  |  |  | Analyst III | 83.56 | 92.54 | 86.93 | 98.15 | 84.87 | 89.21 | 6.06 | **6.79** |  |  |  |  |  |  |
|  |  |  | Mean of Analysts | 89.90 | 89.09 | 89.62 | 91.18 | 89.58 | **Mean (general)** | | |  |  |  |  |  |  |
|  |  |  | SD of Analysts | 7.02 | 4.32 | 5.81 | 6.04 | 4.46 | **89.87** | | |  |  |  |  |  |  |
|  |  |  | **RSD of Analysts (%)** | **7.81** | **4.85** | **6.49** | **6.62** | **4.98** |  |  |  |  |  |  |  |  |  |
|  |  | 1000 | Analyst I | 950.24 | 871.07 | 915.32 | 998.42 | 868.24 | 920.66 | 55.13 | **5.99** | 741.14 | 4993.42 | -1417.43 | 59.80 | 0.0641 |  |
|  |  |  | Analyst II | 877.88 | 931.24 | 867.59 | 954.58 | 1014.12 | 929.08 | 59.77 | **6.43** |  |  |  |  |  |  |
|  |  |  | Analyst III | 987.54 | 1050.23 | 969.24 | 877.44 | 865.94 | 950.08 | 77.71 | **8.18** |  |  |  |  |  |  |
|  |  |  | Mean of Analysts | 938.55 | 950.85 | 917.38 | 943.48 | 916.10 | **Mean (general)** | | |  |  |  |  |  |  |
|  |  |  | SD of Analysts | 55.76 | 91.18 | 50.86 | 61.25 | 84.90 | **933.27** | | |  |  |  |  |  |  |
|  |  |  | **RSD of Analysts (%)** | **5.94** | **9.59** | **5.54** | **6.49** | **9.27** |  |  |  |  |  |  |  |  |  |

| 7 | Trichlromethane | 100 | Analyst I | 86.93 | 82.22 | 81.64 | 88.67 | 91.24 | 86.14 | 4.14 | **4.81** | 3.94 | 28.16 | -8.08 | 4.48 | 0.0511 | **0.050** |
| --- | --- | --- | --- | --- | --- | --- | --- | --- | --- | --- | --- | --- | --- | --- | --- | --- | --- |
|  |  |  | Analyst II | 83.46 | 89.54 | 93.47 | 82.97 | 86.65 | 87.22 | 4.39 | **5.03** |  |  |  |  |  |  |
|  |  |  | Analyst III | 96.27 | 86.65 | 87.80 | 94.87 | 84.16 | 89.95 | 5.32 | **5.91** |  |  |  |  |  |  |
|  |  |  | Mean of Analysts | 88.89 | 86.14 | 87.64 | 88.84 | 87.35 | **Mean (general)** | | |  |  |  |  |  |  |
|  |  |  | SD of Analysts | 6.63 | 3.69 | 5.92 | 5.95 | 3.59 | **87.77** | | |  |  |  |  |  |  |
|  |  |  | **RSD of Analysts (%)** | **7.45** | **4.28** | **6.75** | **6.70** | **4.11** |  |  |  |  |  |  |  |  |  |
|  |  | 1000 | Analyst I | 876.40 | 906.19 | 879.63 | 867.52 | 959.72 | 897.89 | 37.45 | **4.17** | 506.17 | 2867.16 | -787.00 | 45.61 | 0.0496 |  |
|  |  |  | Analyst II | 969.24 | 885.16 | 951.93 | 948.50 | 859.56 | 922.88 | 47.65 | **5.16** |  |  |  |  |  |  |
|  |  |  | Analyst III | 864.51 | 974.42 | 902.69 | 992.76 | 963.41 | 939.56 | 53.87 | **5.73** |  |  |  |  |  |  |
|  |  |  | Mean of Analysts | 903.38 | 921.92 | 911.42 | 936.26 | 927.56 | **Mean (general)** | | |  |  |  |  |  |  |
|  |  |  | SD of Analysts | 57.35 | 46.66 | 36.93 | 63.51 | 58.92 | **920.11** | | |  |  |  |  |  |  |
|  |  |  | **RSD of Analysts (%)** | **6.35** | **5.06** | **4.05** | **6.78** | **6.35** |  |  |  |  |  |  |  |  |  |
| 8 | 1,1,1-trichloroethane | 100 | Analyst I | 91.62 | 88.52 | 84.32 | 88.48 | 81.61 | 86.91 | 3.94 | **4.53** | 14.79 | 15.82 | -0.34 | 3.93 | 0.0449 | **0.052** |
|  |  |  | Analyst II | 82.99 | 94.37 | 88.41 | 90.13 | 86.68 | 88.52 | 4.21 | **4.75** |  |  |  |  |  |  |
|  |  |  | Analyst III | 93.62 | 84.97 | 89.79 | 84.03 | 83.44 | 87.17 | 4.39 | **5.03** |  |  |  |  |  |  |
|  |  |  | Mean of Analysts | 89.41 | 89.29 | 87.51 | 87.55 | 83.91 | **Mean (general)** | | |  |  |  |  |  |  |
|  |  |  | SD of Analysts | 5.65 | 4.74 | 2.84 | 3.16 | 2.57 | **87.53** | | |  |  |  |  |  |  |
|  |  |  | **RSD of Analysts (%)** | **6.32** | **5.31** | **3.25** | **3.60** | **3.07** |  |  |  |  |  |  |  |  |  |
|  |  | 1000 | Analyst I | 950.24 | 888.42 | 906.84 | 834.15 | 1010.23 | 917.98 | 66.29 | **7.22** | 2029.94 | 3280.57 | -416.88 | 53.51 | 0.0589 |  |
|  |  |  | Analyst II | 847.25 | 931.24 | 863.21 | 920.45 | 874.89 | 887.41 | 36.63 | **4.13** |  |  |  |  |  |  |
|  |  |  | Analyst III | 974.56 | 845.34 | 978.75 | 866.47 | 924.68 | 917.96 | 60.97 | **6.64** |  |  |  |  |  |  |
|  |  |  | Mean of Analysts | 924.02 | 888.33 | 916.27 | 873.69 | 936.60 | **Mean (general)** | | |  |  |  |  |  |  |
|  |  |  | SD of Analysts | 67.58 | 42.95 | 58.34 | 43.60 | 68.45 | **907.78** | | |  |  |  |  |  |  |
|  |  |  | **RSD of Analysts (%)** | **7.31** | **4.83** | **6.37** | **4.99** | **7.31** |  |  |  |  |  |  |  |  |  |
| 9 | 1,1-dichloro-1-propene | 100 | Analyst I | 78.70 | 82.02 | 88.41 | 90.13 | 86.68 | 85.19 | 4.72 | **5.54** | 14.56 | 24.29 | -3.24 | 4.59 | 0.0539 | **0.060** |
|  |  |  | Analyst II | 93.62 | 79.49 | 86.54 | 84.03 | 83.44 | 85.42 | 5.23 | **6.12** |  |  |  |  |  |  |
|  |  |  | Analyst III | 89.60 | 85.84 | 77.13 | 88.09 | 82.12 | 84.56 | 5.02 | **5.93** |  |  |  |  |  |  |
|  |  |  | Mean of Analysts | 87.31 | 82.45 | 84.03 | 87.41 | 84.08 | **Mean (general)** | | |  |  |  |  |  |  |
|  |  |  | SD of Analysts | 7.72 | 3.20 | 6.05 | 3.10 | 2.35 | **85.06** | | |  |  |  |  |  |  |
|  |  |  | **RSD of Analysts (%)** | **8.84** | **3.88** | **7.19** | **3.55** | **2.79** |  |  |  |  |  |  |  |  |  |
|  |  | 1000 | Analyst I | 890.24 | 850.21 | 984.71 | 770.02 | 816.41 | 862.32 | 81.44 | **9.44** | 6441.29 | 1515.41 | 1641.96 | 56.19 | 0.0646 |  |
|  |  |  | Analyst II | 893.26 | 872.44 | 949.28 | 823.73 | 822.80 | 872.30 | 52.86 | **6.06** |  |  |  |  |  |  |
|  |  |  | Analyst III | 858.57 | 837.43 | 899.14 | 879.83 | 897.45 | 874.49 | 26.41 | **3.02** |  |  |  |  |  |  |
|  |  |  | Mean of Analysts | 880.69 | 853.36 | 944.38 | 824.53 | 845.56 | **Mean (general)** | | |  |  |  |  |  |  |
|  |  |  | SD of Analysts | 19.22 | 17.71 | 43.00 | 54.91 | 45.06 | **869.70** | | |  |  |  |  |  |  |
|  |  |  | **RSD of Analysts (%)** | **2.18** | **2.08** | **4.55** | **6.66** | **5.33** |  |  |  |  |  |  |  |  |  |

| **S.10.** continuous… | | | | | | | | | | | | | | | | | |
| --- | --- | --- | --- | --- | --- | --- | --- | --- | --- | --- | --- | --- | --- | --- | --- | --- | --- |
| **No** | **VOCs** | ***Spike Conc. µg/L*** | **Analysts** | **Recoveries, µg/L** | | | | | **Intra Days Control** | | | **Sd^2^** | **Sr^2^** | **S_L_^2^** | **S_WR_** | **RSD_WR_** | **RSD_WRpool_** |
|  |  |  |  | **1st Day** | **2nd Day** | **3rd Day** | **4th Day** | **5th Day** | **Mean** | **SD** | **RSD %** |  |  |  |  |  |  |

| **S.10.** continuous… | | | | | | | | | | | | | | | | | |
| --- | --- | --- | --- | --- | --- | --- | --- | --- | --- | --- | --- | --- | --- | --- | --- | --- | --- |
| **No** | **VOCs** | ***Spike Conc. µg/L*** | **Analysts** | **Recoveries, µg/L** | | | | | **Intra Days Control** | | | **Sd^2^** | **Sr^2^** | **S_L_^2^** | **S_WR_** | **RSD_WR_** | **RSD_WRpool_** |
|  |  |  |  | **1st Day** | **2nd Day** | **3rd Day** | **4th Day** | **5th Day** | **Mean** | **SD** | **RSD %** |  |  |  |  |  |  |

| 10 | Benzene | 100 | Analyst I | 83.70 | 80.60 | 91.14 | 85.67 | 82.41 | 84.70 | 4.04 | **4.78** | 28.43 | 21.53 | 2.30 | 4.88 | | 0.0579 | | **0.084** |
| --- | --- | --- | --- | --- | --- | --- | --- | --- | --- | --- | --- | --- | --- | --- | --- | --- | --- | --- | --- |
|  |  |  | Analyst II | 79.92 | 82.03 | 87.53 | 88.57 | 80.39 | 83.69 | 4.08 | **4.87** |  |  |  |  |  |  |  |  |
|  |  |  | Analyst III | 93.57 | 77.96 | 87.05 | 77.32 | 87.78 | 84.74 | 6.96 | **8.21** |  |  |  |  |  |  |  |  |
|  |  |  | Mean of Analysts | 85.73 | 80.20 | 88.57 | 83.85 | 83.53 | **Mean (general)** | | |  |  |  |  |  |  |  |  |
|  |  |  | SD of Analysts | 7.05 | 2.06 | 2.23 | 5.84 | 3.82 | **84.38** | | |  |  |  |  |  |  |  |  |
|  |  |  | **RSD of Analysts (%)** | **8.22** | **2.57** | **2.52** | **6.97** | **4.58** |  |  |  |  |  |  |  |  |  |  |  |
|  |  | 1000 | Analyst I | 1046.40 | 864.52 | 978.89 | 936.78 | 861.65 | 937.65 | 78.51 | **8.37** | 10750.93 | 8109.75 | 880.39 | 94.82 | | 0.1040 | |  |
|  |  |  | Analyst II | 878.77 | 1088.26 | 959.33 | 796.71 | 933.63 | 931.34 | 107.59 | **11.55** |  |  |  |  |  |  |  |  |
|  |  |  | Analyst III | 867.24 | 850.21 | 1025.47 | 770.02 | 816.41 | 865.87 | 96.63 | **11.16** |  |  |  |  |  |  |  |  |
|  |  |  | Mean of Analysts | 930.80 | 934.33 | 987.90 | 834.50 | 870.56 | **Mean (general)** | | |  |  |  |  |  |  |  |  |
|  |  |  | SD of Analysts | 100.27 | 133.50 | 33.98 | 89.57 | 59.11 | **911.62** | | |  |  |  |  |  |  |  |  |
|  |  |  | **RSD of Analysts (%)** | **10.77** | **14.29** | **3.44** | **10.73** | **6.79** |  |  |  |  |  |  |  |  |  |  |  |
| 11 | Tetrachloro methane | 100 | Analyst I | 77.72 | 82.02 | 88.41 | 90.13 | 86.68 | 84.99 | 5.06 | **5.96** | 15.34 | 28.08 | -4.25 | 4.88 | | 0.0575 | | **0.061** |
|  |  |  | Analyst II | 93.62 | 78.50 | 86.54 | 84.03 | 83.44 | 85.22 | 5.52 | **6.48** |  |  |  |  |  |  |  |  |
|  |  |  | Analyst III | 89.60 | 85.84 | 76.17 | 88.09 | 82.12 | 84.36 | 5.38 | **6.37** |  |  |  |  |  |  |  |  |
|  |  |  | Mean of Analysts | 86.98 | 82.12 | 83.71 | 87.41 | 84.08 | **Mean (general)** | | |  |  |  |  |  |  |  |  |
|  |  |  | SD of Analysts | 8.26 | 3.67 | 6.59 | 3.10 | 2.35 | **84.86** | | |  |  |  |  |  |  |  |  |
|  |  |  | **RSD of Analysts (%)** | **9.50** | **4.47** | **7.88** | **3.55** | **2.79** |  |  |  |  |  |  |  |  |  |  |  |
|  |  | 1000 | Analyst I | 906.40 | 831.70 | 895.26 | 812.46 | 854.07 | 859.98 | 40.29 | **4.68** | 4494.56 | 2579.42 | 638.38 | 56.73 | | 0.0638 | |  |
|  |  |  | Analyst II | 926.44 | 888.27 | 954.77 | 853.64 | 822.73 | 889.17 | 53.31 | **6.00** |  |  |  |  |  |  |  |  |
|  |  |  | Analyst III | 946.40 | 888.42 | 915.46 | 834.15 | 1010.23 | 918.93 | 65.61 | **7.14** |  |  |  |  |  |  |  |  |
|  |  |  | Mean of Analysts | 926.41 | 869.46 | 921.83 | 833.42 | 895.68 | **Mean (general)** | | |  |  |  |  |  |  |  |  |
|  |  |  | SD of Analysts | 20.00 | 32.70 | 30.26 | 20.60 | 100.44 | **889.36** | | |  |  |  |  |  |  |  |  |
|  |  |  | **RSD of Analysts (%)** | **2.16** | **3.76** | **3.28** | **2.47** | **11.21** |  |  |  |  |  |  |  |  |  |  |  |
| 12 | Trichloro ethene | 100 | Analyst I | 88.70 | 78.18 | 100.29 | 81.39 | 81.58 | 86.03 | 8.85 | **10.29** | 31.06 | 33.07 | -0.67 | | 5.69 | | 0.0656 | **0.058** |
|  |  |  | Analyst II | 82.46 | 87.82 | 85.40 | 89.39 | 93.99 | 87.81 | 4.33 | **4.94** |  |  |  |  |  |  |  |  |
|  |  |  | Analyst III | 90.48 | 81.71 | 86.93 | 83.17 | 89.22 | 86.30 | 3.78 | **4.38** |  |  |  |  |  |  |  |  |
|  |  |  | Mean of Analysts | 87.21 | 82.57 | 90.87 | 84.65 | 88.27 | **Mean (general)** | | |  |  |  |  |  |  |  |  |
|  |  |  | SD of Analysts | 4.21 | 4.88 | 8.19 | 4.20 | 6.26 | **86.71** | | |  |  |  |  |  |  |  |  |
|  |  |  | **RSD of Analysts (%)** | **4.83** | **5.90** | **9.01** | **4.96** | **7.09** |  |  |  |  |  |  |  |  |  |  |  |
|  |  | 1000 | Analyst I | 926.40 | 879.00 | 914.82 | 824.14 | 950.12 | 898.90 | 49.04 | **5.46** | 1189.19 | 2570.40 | -460.40 | | 45.93 | | 0.0500 |  |
|  |  |  | Analyst II | 988.63 | 907.87 | 990.01 | 901.07 | 850.96 | 927.71 | 60.39 | **6.51** |  |  |  |  |  |  |  |  |
|  |  |  | Analyst III | 881.80 | 945.19 | 917.14 | 943.13 | 953.78 | 928.21 | 29.33 | **3.16** |  |  |  |  |  |  |  |  |
|  |  |  | Mean of Analysts | 932.28 | 910.69 | 940.66 | 889.45 | 918.29 | **Mean (general)** | | |  |  |  |  |  |  |  |  |
|  |  |  | SD of Analysts | 53.66 | 33.18 | 42.76 | 60.34 | 58.33 | **918.27** | | |  |  |  |  |  |  |  |  |
|  |  |  | **RSD of Analysts (%)** | **5.76** | **3.64** | **4.55** | **6.78** | **6.35** |  |  |  |  |  |  |  |  |  |  |  |

| **S.10.** continuous… | | | | | | | | | | | | | | | | | |
| --- | --- | --- | --- | --- | --- | --- | --- | --- | --- | --- | --- | --- | --- | --- | --- | --- | --- |
| **No** | **VOCs** | ***Spike Conc. µg/L*** | **Analysts** | **Recoveries, µg/L** | | | | | **Intra Days Control** | | | **Sd^2^** | **Sr^2^** | **S_L_^2^** | **S_WR_** | **RSD_WR_** | **RSD_WRpool_** |
|  |  |  |  | **1st Day** | **2nd Day** | **3rd Day** | **4th Day** | **5th Day** | **Mean** | **SD** | **RSD %** |  |  |  |  |  |  |

| 13 | 1,2-dichloro propane | 100 | Analyst I | 85.23 | 89.76 | 90.41 | 93.24 | 84.02 | 88.53 | 3.82 | **4.32** | 15.07 | 26.44 | -3.79 | 4.76 | 0.0547 | **0.078** |
| --- | --- | --- | --- | --- | --- | --- | --- | --- | --- | --- | --- | --- | --- | --- | --- | --- | --- |
|  |  |  | Analyst II | 88.67 | 79.75 | 84.91 | 84.24 | 90.33 | 85.58 | 4.13 | **4.83** |  |  |  |  |  |  |
|  |  |  | Analyst III | 85.13 | 86.85 | 97.21 | 78.82 | 85.78 | 86.76 | 6.63 | **7.64** |  |  |  |  |  |  |
|  |  |  | Mean of Analysts | 86.34 | 85.46 | 90.84 | 85.43 | 86.71 | **Mean (general)** | | |  |  |  |  |  |  |
|  |  |  | SD of Analysts | 2.01 | 5.15 | 6.16 | 7.28 | 3.25 | **86.96** | | |  |  |  |  |  |  |
|  |  |  | **RSD of Analysts (%)** | **2.33** | **6.03** | **6.78** | **8.53** | **3.75** |  |  |  |  |  |  |  |  |  |
|  |  | 1000 | Analyst I | 876.40 | 864.52 | 978.89 | 936.78 | 861.65 | 903.65 | 51.95 | **5.75** | 6705.60 | 7666.12 | -320.17 | 85.71 | 0.0950 |  |
|  |  |  | Analyst II | 1088.12 | 885.16 | 959.33 | 796.71 | 933.63 | 932.59 | 106.79 | **11.45** |  |  |  |  |  |  |
|  |  |  | Analyst III | 867.24 | 1025.47 | 867.64 | 770.02 | 816.41 | 869.36 | 96.24 | **11.07** |  |  |  |  |  |  |
|  |  |  | Mean of Analysts | 943.92 | 925.05 | 935.28 | 834.50 | 870.56 | **Mean (general)** | | |  |  |  |  |  |  |
|  |  |  | SD of Analysts | 124.96 | 87.57 | 59.40 | 89.57 | 59.11 | **901.86** | | |  |  |  |  |  |  |
|  |  |  | **RSD of Analysts (%)** | **13.24** | **9.47** | **6.35** | **10.73** | **6.79** |  |  |  |  |  |  |  |  |  |
| 14 | Dibromo methane | 100 | Analyst I | 100.70 | 93.48 | 87.94 | 103.42 | 88.56 | 94.82 | 7.01 | **7.40** | 11.28 | 80.96 | -23.23 | 7.60 | 0.0805 | **0.087** |
|  |  |  | Analyst II | 89.65 | 104.72 | 94.78 | 91.89 | 97.24 | 95.66 | 5.83 | **6.09** |  |  |  |  |  |  |
|  |  |  | Analyst III | 88.46 | 79.57 | 101.70 | 86.87 | 106.57 | 92.63 | 11.16 | **12.04** |  |  |  |  |  |  |
|  |  |  | Mean of Analysts | 92.94 | 92.59 | 94.81 | 94.06 | 97.46 | **Mean (general)** | | |  |  |  |  |  |  |
|  |  |  | SD of Analysts | 6.75 | 12.60 | 6.88 | 8.49 | 9.01 | **94.37** | | |  |  |  |  |  |  |
|  |  |  | **RSD of Analysts (%)** | **7.26** | **13.61** | **7.26** | **9.02** | **9.24** |  |  |  |  |  |  |  |  |  |
|  |  | 1000 | Analyst I | 942.60 | 806.75 | 912.08 | 771.84 | 845.53 | 855.76 | 71.17 | **8.32** | 12405.45 | 3930.50 | 2824.98 | 82.19 | 0.0925 |  |
|  |  |  | Analyst II | 876.87 | 952.03 | 992.96 | 810.96 | 814.50 | 889.46 | 81.50 | **9.16** |  |  |  |  |  |  |
|  |  |  | Analyst III | 965.33 | 861.77 | 980.30 | 792.44 | 1000.13 | 919.99 | 89.13 | **9.69** |  |  |  |  |  |  |
|  |  |  | Mean of Analysts | 928.3 | 873.5 | 961.8 | 791.7 | 886.7 | **Mean (general)** | | |  |  |  |  |  |  |
|  |  |  | SD of Analysts | 45.9 | 73.3 | 43.5 | 19.6 | 99.4 | **888.41** | | |  |  |  |  |  |  |
|  |  |  | **RSD of Analysts (%)** | **4.9** | **8.4** | **4.5** | **2.5** | **11.2** |  |  |  |  |  |  |  |  |  |
| 15 | Bromodichloromethane | 100 | Analyst I | 82.16 | 85.84 | 77.13 | 88.09 | 89.60 | 84.56 | 5.01 | **5.93** | 13.47 | 20.27 | -2.27 | 4.24 | 0.0500 | **0.069** |
|  |  |  | Analyst II | 91.14 | 84.63 | 85.64 | 82.75 | 82.41 | 85.31 | 3.52 | **4.13** |  |  |  |  |  |  |
|  |  |  | Analyst III | 79.92 | 91.45 | 82.98 | 88.57 | 80.39 | 84.66 | 5.12 | **6.05** |  |  |  |  |  |  |
|  |  |  | Mean of Analysts | 84.41 | 87.30 | 81.92 | 86.47 | 84.13 | **Mean (general)** | | |  |  |  |  |  |  |
|  |  |  | SD of Analysts | 5.94 | 3.64 | 4.35 | 3.23 | 4.84 | **84.85** | | |  |  |  |  |  |  |
|  |  |  | **RSD of Analysts (%)** | **7.04** | **4.17** | **5.32** | **3.73** | **5.76** |  |  |  |  |  |  |  |  |  |
|  |  | 1000 | Analyst I | 956.40 | 837.43 | 899.14 | 879.83 | 897.45 | 894.05 | 42.82 | **4.79** | 5394.27 | 6264.87 | -290.20 | 77.30 | 0.0832 |  |
|  |  |  | Analyst II | 1046.40 | 965.96 | 978.89 | 936.78 | 861.65 | 957.94 | 67.17 | **7.01** |  |  |  |  |  |  |
|  |  |  | Analyst III | 878.77 | 1088.26 | 985.09 | 796.71 | 933.63 | 936.49 | 109.86 | **11.73** |  |  |  |  |  |  |
|  |  |  | Mean of Analysts | 960.52 | 963.88 | 954.37 | 871.11 | 897.58 | **Mean (general)** | | |  |  |  |  |  |  |
|  |  |  | SD of Analysts | 83.89 | 125.43 | 47.93 | 70.44 | 35.99 | **929.49** | | |  |  |  |  |  |  |
|  |  |  | **RSD of Analysts (%)** | **8.73** | **13.01** | **5.02** | **8.09** | **4.01** |  |  |  |  |  |  |  |  |  |

| **S.10.** continuous… | | | | | | | | | | | | | | | | | |
| --- | --- | --- | --- | --- | --- | --- | --- | --- | --- | --- | --- | --- | --- | --- | --- | --- | --- |
| **No** | **VOCs** | ***Spike Conc. µg/L*** | **Analysts** | **Recoveries, µg/L** | | | | | **Intra Days Control** | | | **Sd^2^** | **Sr^2^** | **S_L_^2^** | **S_WR_** | **RSD_WR_** | **RSD_WRpool_** |
|  |  |  |  | **1st Day** | **2nd Day** | **3rd Day** | **4th Day** | **5th Day** | **Mean** | **SD** | **RSD %** |  |  |  |  |  |  |

| 16 | 1,3-dichloro propene (cis+trans) | 100 | Analyst I | 90.48 | 75.83 | 104.30 | 77.32 | 80.77 | 85.74 | 11.84 | **13.81** | 111.58 | 33.53 | 26.02 | 7.72 | 0.0894 | **0.085** |
| --- | --- | --- | --- | --- | --- | --- | --- | --- | --- | --- | --- | --- | --- | --- | --- | --- | --- |
|  |  |  | Analyst II | 84.11 | 85.18 | 88.82 | 84.92 | 93.05 | 87.22 | 3.73 | **4.28** |  |  |  |  |  |  |
|  |  |  | Analyst III | 92.29 | 79.26 | 90.41 | 79.01 | 88.33 | 85.86 | 6.29 | **7.33** |  |  |  |  |  |  |
|  |  |  | Mean of Analysts | 88.96 | 80.09 | 94.51 | 80.42 | 87.38 | **Mean (general)** | | |  |  |  |  |  |  |
|  |  |  | SD of Analysts | 4.30 | 4.73 | 8.52 | 3.99 | 6.20 | **86.27** | | |  |  |  |  |  |  |
|  |  |  | **RSD of Analysts (%)** | **4.83** | **5.90** | **9.01** | **4.96** | **7.09** |  |  |  |  |  |  |  |  |  |
|  |  | 1000 | Analyst I | 926.40 | 861.62 | 992.96 | 810.96 | 814.50 | 881.29 | 77.94 | **8.84** | 1173.61 | 7148.09 | -1991.49 | 71.81 | 0.0813 |  |
|  |  |  | Analyst II | 792.45 | 935.66 | 792.44 | 950.87 | 1000.13 | 894.31 | 95.99 | **10.73** |  |  |  |  |  |  |
|  |  |  | Analyst III | 944.94 | 843.38 | 907.87 | 791.75 | 886.72 | 874.93 | 59.25 | **6.77** |  |  |  |  |  |  |
|  |  |  | Mean of Analysts | 887.93 | 880.22 | 897.76 | 851.19 | 900.45 | **Mean (general)** | | |  |  |  |  |  |  |
|  |  |  | SD of Analysts | 83.21 | 48.87 | 100.64 | 86.86 | 93.57 | **883.51** | | |  |  |  |  |  |  |
|  |  |  | **RSD of Analysts (%)** | **9.37** | **5.55** | **11.21** | **10.20** | **10.39** |  |  |  |  |  |  |  |  |  |
| 17 | Toluen | 100 | Analyst I | 92.14 | 94.88 | 87.98 | 77.18 | 85.47 | 87.53 | 6.84 | **7.81** | 68.12 | 18.24 | 16.63 | 5.90 | 0.0678 | **0.094** |
|  |  |  | Analyst II | 82.70 | 93.06 | 90.60 | 82.02 | 81.15 | 85.91 | 5.50 | **6.41** |  |  |  |  |  |  |
|  |  |  | Analyst III | 86.88 | 85.75 | 95.83 | 80.26 | 90.83 | 87.91 | 5.82 | **6.62** |  |  |  |  |  |  |
|  |  |  | Mean of Analysts | 87.24 | 91.23 | 91.47 | 79.82 | 85.82 | **Mean (general)** | | |  |  |  |  |  |  |
|  |  |  | SD of Analysts | 4.73 | 4.83 | 3.99 | 2.45 | 4.85 | **87.11** | | |  |  |  |  |  |  |
|  |  |  | RSD of Analysts (%) | **5.42** | **5.30** | **4.36** | **3.07** | **5.65** |  |  |  |  |  |  |  |  |  |
|  |  | 1000 | Analyst I | 1102.60 | 946.78 | 829.05 | 910.74 | 840.63 | 925.96 | 110.16 | **11.90** | 5991.49 | 13971.62 | -2660.04 | 106.36 | 0.1140 |  |
|  |  |  | Analyst II | 877.90 | 1146.70 | 922.42 | 936.78 | 887.76 | 954.31 | 110.24 | **11.55** |  |  |  |  |  |  |
|  |  |  | Analyst III | 835.69 | 882.90 | 1113.63 | 796.71 | 961.92 | 918.17 | 125.42 | **13.66** |  |  |  |  |  |  |
|  |  |  | Mean of Analysts | 938.73 | 992.13 | 955.03 | 881.41 | 896.77 | **Mean (general)** | | |  |  |  |  |  |  |
|  |  |  | SD of Analysts | 143.48 | 137.62 | 145.06 | 74.50 | 61.14 | **932.81** | | |  |  |  |  |  |  |
|  |  |  | **RSD of Analysts (%)** | **15.28** | **13.87** | **15.19** | **8.45** | **6.82** |  |  |  |  |  |  |  |  |  |
| 18 | 1,1,2-trichloro ethane | 100 | Analyst I | 85.21 | 92.03 | 91.50 | 88.09 | 84.61 | 88.29 | 3.44 | **3.90** | 26.35 | 35.81 | -3.15 | 5.71 | 0.0654 | **0.075** |
|  |  |  | Analyst II | 78.30 | 83.82 | 94.50 | 81.39 | 84.06 | 84.41 | 6.10 | **7.23** |  |  |  |  |  |  |
|  |  |  | Analyst III | 85.12 | 95.92 | 80.48 | 89.39 | 96.84 | 89.55 | 6.99 | **7.81** |  |  |  |  |  |  |
|  |  |  | Mean of Analysts | 82.88 | 90.59 | 88.83 | 86.29 | 88.50 | **Mean (general)** | | |  |  |  |  |  |  |
|  |  |  | SD of Analysts | 3.96 | 6.18 | 7.39 | 4.29 | 7.23 | **87.42** | | |  |  |  |  |  |  |
|  |  |  | **RSD of Analysts (%)** | **4.78** | **6.82** | **8.31** | **4.97** | **8.17** |  |  |  |  |  |  |  |  |  |
|  |  | 1000 | Analyst I | 918.01 | 923.96 | 888.70 | 792.44 | 1030.43 | 910.71 | 85.20 | **9.36** | 9091.39 | 3716.70 | 1791.56 | 74.22 | 0.0829 |  |
|  |  |  | Analyst II | 821.83 | 1023.63 | 845.95 | 874.43 | 892.39 | 891.64 | 78.55 | **8.81** |  |  |  |  |  |  |
|  |  |  | Analyst III | 810.22 | 879.15 | 955.07 | 823.15 | 943.17 | 882.15 | 66.53 | **7.54** |  |  |  |  |  |  |
|  |  |  | Mean of Analysts | 850.02 | 942.25 | 896.57 | 830.01 | 955.33 | **Mean (general)** | | |  |  |  |  |  |  |
|  |  |  | SD of Analysts | 59.16 | 73.95 | 54.99 | 41.42 | 69.82 | **894.84** | | |  |  |  |  |  |  |
|  |  |  | **RSD of Analysts (%)** | **6.96** | **7.85** | **6.13** | **4.99** | **7.31** |  |  |  |  |  |  |  |  |  |

| **S.10.** continuous… | | | | | | | | | | | | | | | | | |
| --- | --- | --- | --- | --- | --- | --- | --- | --- | --- | --- | --- | --- | --- | --- | --- | --- | --- |
| **No** | **VOCs** | ***Spike Conc. µg/L*** | **Analysts** | **Recoveries, µg/L** | | | | | **Intra Days Control** | | | **Sd^2^** | **Sr^2^** | **S_L_^2^** | **S_WR_** | **RSD_WR_** | **RSD_WRpool_** |
|  |  |  |  | **1st Day** | **2nd Day** | **3rd Day** | **4th Day** | **5th Day** | **Mean** | **SD** | **RSD %** |  |  |  |  |  |  |

| 19 | Tetrachloro ethene | 100 | Analyst I | 73.22 | 92.03 | 91.50 | 88.09 | 84.61 | 85.89 | 7.68 | **8.95** | 42.82 | 77.25 | -11.48 | 8.11 | 0.0949 | **0.085** |
| --- | --- | --- | --- | --- | --- | --- | --- | --- | --- | --- | --- | --- | --- | --- | --- | --- | --- |
|  |  |  | Analyst II | 78.30 | 73.95 | 94.50 | 81.39 | 84.06 | 82.44 | 7.72 | **9.36** |  |  |  |  |  |  |
|  |  |  | Analyst III | 85.12 | 95.92 | 72.49 | 89.39 | 96.84 | 87.95 | 9.89 | **11.25** |  |  |  |  |  |  |
|  |  |  | Mean of Analysts | 78.88 | 87.30 | 86.17 | 86.29 | 88.50 | **Mean (general)** | | |  |  |  |  |  |  |
|  |  |  | SD of Analysts | 5.97 | 11.72 | 11.94 | 4.29 | 7.23 | **85.43** | | |  |  |  |  |  |  |
|  |  |  | **RSD of Analysts (%)** | **7.57** | **13.43** | **13.85** | **4.97** | **8.17** |  |  |  |  |  |  |  |  |  |
|  |  | 1000 | Analyst I | 870.24 | 884.21 | 965.02 | 731.52 | 832.74 | 856.75 | 85.03 | **9.92** | 8414.57 | 2003.76 | 2136.94 | 64.35 | 0.0742 |  |
|  |  |  | Analyst II | 912.45 | 861.54 | 930.30 | 782.54 | 839.25 | 865.22 | 59.14 | **6.84** |  |  |  |  |  |  |
|  |  |  | Analyst III | 832.82 | 942.03 | 878.94 | 835.84 | 915.40 | 881.01 | 48.15 | **5.47** |  |  |  |  |  |  |
|  |  |  | Mean of Analysts | 871.84 | 895.93 | 924.75 | 783.30 | 862.47 | **Mean (general)** | | |  |  |  |  |  |  |
|  |  |  | SD of Analysts | 39.84 | 41.51 | 43.31 | 52.16 | 45.96 | **867.66** | | |  |  |  |  |  |  |
|  |  |  | **RSD of Analysts (%)** | **4.57** | **4.63** | **4.68** | **6.66** | **5.33** |  |  |  |  |  |  |  |  |  |
| 20 | 1,3-dichloro propane | 100 | Analyst I | 87.02 | 92.03 | 91.50 | 88.09 | 84.61 | 88.65 | 3.12 | **3.52** | 37.66 | 23.55 | 4.70 | 5.32 | 0.0602 | **0.069** |
|  |  |  | Analyst II | 78.30 | 87.89 | 94.50 | 81.39 | 84.06 | 85.23 | 6.27 | **7.36** |  |  |  |  |  |  |
|  |  |  | Analyst III | 85.12 | 95.92 | 88.19 | 89.39 | 96.84 | 91.09 | 5.08 | **5.58** |  |  |  |  |  |  |
|  |  |  | Mean of Analysts | 83.48 | 91.95 | 91.40 | 86.29 | 88.50 | **Mean (general)** | | |  |  |  |  |  |  |
|  |  |  | SD of Analysts | 4.59 | 4.01 | 3.16 | 4.29 | 7.23 | **88.32** | | |  |  |  |  |  |  |
|  |  |  | **RSD of Analysts (%)** | **5.49** | **4.36** | **3.46** | **4.97** | **8.17** |  |  |  |  |  |  |  |  |  |
|  |  | 1000 | Analyst I | 920.24 | 923.96 | 888.70 | 792.44 | 1030.43 | 911.16 | 85.25 | **9.36** | 6386.70 | 4110.39 | 758.77 | 69.78 | 0.0775 |  |
|  |  |  | Analyst II | 1023.07 | 929.44 | 845.95 | 874.43 | 892.39 | 913.05 | 68.55 | **7.51** |  |  |  |  |  |  |
|  |  |  | Analyst III | 810.22 | 879.15 | 932.42 | 823.15 | 943.17 | 877.62 | 60.85 | **6.93** |  |  |  |  |  |  |
|  |  |  | Mean of Analysts | 917.84 | 910.85 | 889.02 | 830.01 | 955.33 | **Mean (general)** | | |  |  |  |  |  |  |
|  |  |  | SD of Analysts | 106.44 | 27.59 | 43.24 | 41.42 | 69.82 | **900.61** | | |  |  |  |  |  |  |
|  |  |  | **RSD of Analysts (%)** | **11.60** | **3.03** | **4.86** | **4.99** | **7.31** |  |  |  |  |  |  |  |  |  |
| 21 | Dibromochloromethane | 100 | Analyst I | 92.16 | 87.61 | 94.36 | 83.17 | 91.92 | 89.85 | 4.46 | **4.97** | 13.27 | 33.41 | -6.71 | 5.17 | 0.0574 | **0.067** |
|  |  |  | Analyst II | 94.53 | 90.32 | 83.93 | 83.45 | 95.61 | 89.57 | 5.72 | **6.39** |  |  |  |  |  |  |
|  |  |  | Analyst III | 81.05 | 96.24 | 95.85 | 93.24 | 86.57 | 90.59 | 6.59 | **7.28** |  |  |  |  |  |  |
|  |  |  | Mean of Analysts | 89.25 | 91.39 | 91.38 | 86.62 | 91.37 | **Mean (general)** | | |  |  |  |  |  |  |
|  |  |  | SD of Analysts | 7.19 | 4.41 | 6.50 | 5.74 | 4.55 | **90.00** | | |  |  |  |  |  |  |
|  |  |  | **RSD of Analysts (%)** | **8.06** | **4.83** | **7.11** | **6.62** | **4.98** |  |  |  |  |  |  |  |  |  |
|  |  | 1000 | Analyst I | 916.40 | 905.91 | 897.01 | 948.50 | 885.60 | 910.69 | 23.99 | **2.63** | 4813.25 | 4777.94 | 11.77 | 69.21 | 0.0751 |  |
|  |  |  | Analyst II | 851.54 | 953.06 | 850.24 | 906.85 | 1034.40 | 919.22 | 77.30 | **8.41** |  |  |  |  |  |  |
|  |  |  | Analyst III | 957.91 | 1092.24 | 898.07 | 833.57 | 883.26 | 933.01 | 99.45 | **10.66** |  |  |  |  |  |  |
|  |  |  | Mean of Analysts | 908.62 | 983.74 | 881.77 | 896.31 | 934.42 | **Mean (general)** | | |  |  |  |  |  |  |
|  |  |  | SD of Analysts | 53.61 | 96.88 | 27.32 | 58.19 | 86.59 | **920.97** | | |  |  |  |  |  |  |
|  |  |  | **RSD of Analysts (%)** | **5.90** | **9.85** | **3.10** | **6.49** | **9.27** |  |  |  |  |  |  |  |  |  |

| **S.10.** continuous… | | | | | | | | | | | | | | | | | |
| --- | --- | --- | --- | --- | --- | --- | --- | --- | --- | --- | --- | --- | --- | --- | --- | --- | --- |
| **No** | **VOCs** | ***Spike Conc. µg/L*** | **Analysts** | **Recoveries, µg/L** | | | | | **Intra Days Control** | | | **Sd^2^** | **Sr^2^** | **S_L_^2^** | **S_WR_** | **RSD_WR_** | **RSD_WRpool_** |
|  |  |  |  | **1st Day** | **2nd Day** | **3rd Day** | **4th Day** | **5th Day** | **Mean** | **SD** | **RSD %** |  |  |  |  |  |  |

| 22 | 1,2-dibromoethane | 100 | Analyst I | 91.22 | 85.51 | 80.01 | 84.24 | 93.06 | 86.81 | 5.32 | **6.13** | 13.17 | 33.03 | -6.62 | 5.14 | 0.0583 | **0.062** |
| --- | --- | --- | --- | --- | --- | --- | --- | --- | --- | --- | --- | --- | --- | --- | --- | --- | --- |
|  |  |  | Analyst II | 80.96 | 93.05 | 91.60 | 78.82 | 88.38 | 86.56 | 6.37 | **7.35** |  |  |  |  |  |  |
|  |  |  | Analyst III | 93.38 | 90.12 | 94.91 | 90.13 | 85.84 | 90.88 | 3.50 | **3.85** |  |  |  |  |  |  |
|  |  |  | Mean of Analysts | 88.52 | 89.56 | 88.84 | 84.39 | 89.10 | **Mean (general)** | | |  |  |  |  |  |  |
|  |  |  | SD of Analysts | 6.64 | 3.80 | 7.82 | 5.65 | 3.66 | **88.08** | | |  |  |  |  |  |  |
|  |  |  | **RSD of Analysts (%)** | **7.50** | **4.24** | **8.81** | **6.70** | **4.11** |  |  |  |  |  |  |  |  |  |
|  |  | 1000 | Analyst I | 992.60 | 942.44 | 862.04 | 824.14 | 978.91 | 920.03 | 73.81 | **8.02** | 3013.98 | 4050.91 | -345.65 | 60.87 | 0.0652 |  |
|  |  |  | Analyst II | 940.17 | 972.75 | 932.89 | 901.07 | 876.75 | 924.73 | 36.99 | **4.00** |  |  |  |  |  |  |
|  |  |  | Analyst III | 838.57 | 1013.40 | 1011.66 | 943.13 | 982.68 | 957.89 | 72.53 | **7.57** |  |  |  |  |  |  |
|  |  |  | Mean of Analysts | 923.78 | 976.19 | 935.53 | 889.45 | 946.11 | **Mean (general)** | | |  |  |  |  |  |  |
|  |  |  | SD of Analysts | 78.31 | 35.61 | 74.84 | 60.34 | 60.10 | **934.21** | | |  |  |  |  |  |  |
|  |  |  | **RSD of Analysts (%)** | **8.48** | **3.65** | **8.00** | **6.78** | **6.35** |  |  |  |  |  |  |  |  |  |
| 23 | Chlorobenzene | 100 | Analyst I | 77.16 | 92.06 | 82.64 | 84.06 | 88.87 | 84.96 | 5.76 | **6.78** | 22.71 | 37.48 | -4.93 | 5.71 | 0.0666 | **0.062** |
|  |  |  | Analyst II | 98.14 | 79.48 | 86.64 | 85.62 | 88.42 | 87.66 | 6.76 | **7.71** |  |  |  |  |  |  |
|  |  |  | Analyst III | 90.81 | 88.37 | 77.93 | 79.83 | 85.11 | 84.41 | 5.48 | **6.49** |  |  |  |  |  |  |
|  |  |  | Mean of Analysts | 88.70 | 86.64 | 82.40 | 83.17 | 87.46 | **Mean (general)** | | |  |  |  |  |  |  |
|  |  |  | SD of Analysts | 10.65 | 6.47 | 4.36 | 3.00 | 2.06 | **85.68** | | |  |  |  |  |  |  |
|  |  |  | **RSD of Analysts (%)** | **12.00** | **7.47** | **5.29** | **3.60** | **2.35** |  |  |  |  |  |  |  |  |  |
|  |  | 1000 | Analyst I | 900.24 | 942.44 | 862.04 | 824.14 | 978.91 | 901.55 | 61.67 | **6.84** | 2785.63 | 2850.83 | -21.73 | 53.19 | 0.0579 |  |
|  |  |  | Analyst II | 940.17 | 909.24 | 932.89 | 901.07 | 876.75 | 912.03 | 25.49 | **2.80** |  |  |  |  |  |  |
|  |  |  | Analyst III | 838.57 | 1013.40 | 927.25 | 943.13 | 982.68 | 941.01 | 66.45 | **7.06** |  |  |  |  |  |  |
|  |  |  | Mean of Analysts | 892.99 | 955.03 | 907.39 | 889.45 | 946.11 | **Mean (general)** | | |  |  |  |  |  |  |
|  |  |  | SD of Analysts | 51.18 | 53.21 | 39.38 | 60.34 | 60.10 | **918.19** | | |  |  |  |  |  |  |
|  |  |  | **RSD of Analysts (%)** | **5.73** | **5.57** | **4.34** | **6.78** | **6.35** |  |  |  |  |  |  |  |  |  |
| 24 | 1,1,1,2-tetrachloroethane | 100 | Analyst I | 113.70 | 91.63 | 89.31 | 81.39 | 84.06 | 92.02 | 12.78 | **13.89** | 241.68 | 107.66 | 44.67 | 12.34 | 0.1344 | **0.110** |
|  |  |  | Analyst II | 98.21 | 112.56 | 87.63 | 84.14 | 82.00 | 92.91 | 12.63 | **13.59** |  |  |  |  |  |  |
|  |  |  | Analyst III | 90.76 | 87.64 | 111.42 | 73.45 | 89.54 | 90.56 | 13.58 | **14.99** |  |  |  |  |  |  |
|  |  |  | Mean of Analysts | 100.89 | 97.28 | 96.12 | 79.66 | 85.20 | **Mean (general)** | | |  |  |  |  |  |  |
|  |  |  | SD of Analysts | 11.70 | 13.38 | 13.28 | 5.55 | 3.90 | **91.83** | | |  |  |  |  |  |  |
|  |  |  | **RSD of Analysts (%)** | **11.60** | **13.76** | **13.81** | **6.97** | **4.58** |  |  |  |  |  |  |  |  |  |
|  |  | 1000 | Analyst I | 946.40 | 1015.01 | 959.31 | 889.94 | 878.88 | 937.91 | 55.36 | **5.90** | 5616.45 | 4757.60 | 286.28 | 71.02 | 0.0773 |  |
|  |  |  | Analyst II | 852.41 | 927.47 | 940.14 | 756.87 | 952.30 | 885.84 | 81.93 | **9.25** |  |  |  |  |  |  |
|  |  |  | Analyst III | 1004.35 | 884.21 | 1004.96 | 936.94 | 832.74 | 932.64 | 75.36 | **8.08** |  |  |  |  |  |  |
|  |  |  | Mean of Analysts | 934.39 | 942.23 | 968.14 | 861.25 | 887.97 | **Mean (general)** | | |  |  |  |  |  |  |
|  |  |  | SD of Analysts | 76.68 | 66.64 | 33.30 | 93.40 | 60.30 | **918.80** | | |  |  |  |  |  |  |
|  |  |  | **RSD of Analysts (%)** | **8.21** | **7.07** | **3.44** | **10.84** | **6.79** |  |  |  |  |  |  |  |  |  |

| **S.10.** continuous… | | | | | | | | | | | | | | | | | |
| --- | --- | --- | --- | --- | --- | --- | --- | --- | --- | --- | --- | --- | --- | --- | --- | --- | --- |
| **No** | **VOCs** | ***Spike Conc. µg/L*** | **Analysts** | **Recoveries, µg/L** | | | | | **Intra Days Control** | | | **Sd^2^** | **Sr^2^** | **S_L_^2^** | **S_WR_** | **RSD_WR_** | **RSD_WRpool_** |
|  |  |  |  | **1st Day** | **2nd Day** | **3rd Day** | **4th Day** | **5th Day** | **Mean** | **SD** | **RSD %** |  |  |  |  |  |  |

| 25 | Ethylbenzene | 100 | Analyst I | 81.16 | 81.31 | 98.28 | 77.32 | 83.22 | 84.26 | 8.13 | **9.65** | 45.99 | 40.18 | 1.94 | 6.49 | 0.0762 | **0.071** |
| --- | --- | --- | --- | --- | --- | --- | --- | --- | --- | --- | --- | --- | --- | --- | --- | --- | --- |
|  |  |  | Analyst II | 91.33 | 79.54 | 83.70 | 84.92 | 95.87 | 87.07 | 6.49 | **7.45** |  |  |  |  |  |  |
|  |  |  | Analyst III | 87.76 | 84.98 | 77.95 | 79.01 | 91.00 | 84.14 | 5.60 | **6.66** |  |  |  |  |  |  |
|  |  |  | Mean of Analysts | 86.75 | 81.94 | 86.64 | 80.42 | 90.03 | **Mean (general)** | | |  |  |  |  |  |  |
|  |  |  | SD of Analysts | 5.16 | 2.78 | 10.48 | 3.99 | 6.38 | **85.16** | | |  |  |  |  |  |  |
|  |  |  | **RSD of Analysts (%)** | **5.95** | **3.39** | **12.10** | **4.96** | **7.09** |  |  |  |  |  |  |  |  |  |
|  |  | 1000 | Analyst I | 902.60 | 864.97 | 877.35 | 931.09 | 871.15 | 889.43 | 27.33 | **3.07** | 1878.92 | 4047.73 | -722.94 | 57.66 | 0.0651 |  |
|  |  |  | Analyst II | 843.65 | 884.55 | 935.67 | 810.96 | 839.18 | 862.80 | 48.47 | **5.62** |  |  |  |  |  |  |
|  |  |  | Analyst III | 918.01 | 923.96 | 858.01 | 792.44 | 1030.43 | 904.57 | 88.23 | **9.75** |  |  |  |  |  |  |
|  |  |  | Mean of Analysts | 888.09 | 891.16 | 890.35 | 844.83 | 913.59 | **Mean (general)** | | |  |  |  |  |  |  |
|  |  |  | SD of Analysts | 39.25 | 30.04 | 40.43 | 75.27 | 102.44 | **885.60** | | |  |  |  |  |  |  |
|  |  |  | **RSD of Analysts (%)** | **4.42** | **3.37** | **4.54** | **8.91** | **11.21** |  |  |  |  |  |  |  |  |  |
| 26 | m,p-xylene | 100 | Analyst I | 74.16 | 93.35 | 88.60 | 88.58 | 85.70 | 86.08 | 7.21 | **8.37** | 30.20 | 50.74 | -6.85 | 6.62 | 0.0791 | **0.080** |
|  |  |  | Analyst II | 86.01 | 72.68 | 83.21 | 80.02 | 92.13 | 82.81 | 7.21 | **8.70** |  |  |  |  |  |  |
|  |  |  | Analyst III | 82.58 | 90.33 | 77.13 | 74.88 | 87.50 | 82.48 | 6.58 | **7.98** |  |  |  |  |  |  |
|  |  |  | Mean of Analysts | 80.91 | 85.45 | 82.98 | 81.16 | 88.45 | **Mean (general)** | | |  |  |  |  |  |  |
|  |  |  | SD of Analysts | 6.10 | 11.17 | 5.74 | 6.92 | 3.32 | **83.79** | | |  |  |  |  |  |  |
|  |  |  | **RSD of Analysts (%)** | **7.53** | **13.07** | **6.92** | **8.53** | **3.75** |  |  |  |  |  |  |  |  |  |
|  |  | 1000 | Analyst I | 892.60 | 832.87 | 903.39 | 791.75 | 913.59 | 866.84 | 52.39 | **6.04** | 6646.86 | 4504.20 | 714.22 | 72.24 | 0.0809 |  |
|  |  |  | Analyst II | 850.11 | 928.30 | 959.31 | 889.94 | 878.88 | 901.31 | 42.86 | **4.76** |  |  |  |  |  |  |
|  |  |  | Analyst III | 1055.48 | 920.57 | 874.75 | 756.87 | 952.30 | 911.99 | 109.24 | **11.98** |  |  |  |  |  |  |
|  |  |  | Mean of Analysts | 932.73 | 893.91 | 912.48 | 812.85 | 914.92 | **Mean (general)** | | |  |  |  |  |  |  |
|  |  |  | SD of Analysts | 108.41 | 53.01 | 43.01 | 69.00 | 36.73 | **893.38** | | |  |  |  |  |  |  |
|  |  |  | **RSD of Analysts (%)** | **11.62** | **5.93** | **4.71** | **8.49** | **4.01** |  |  |  |  |  |  |  |  |  |
| 27 | o-xylene | 100 | Analyst I | 75.06 | 97.22 | 86.18 | 98.25 | 90.33 | 89.41 | 9.44 | **10.56** | 115.63 | 75.23 | 13.47 | 9.42 | 0.1071 | **0.093** |
|  |  |  | Analyst II | 86.96 | 75.81 | 92.88 | 87.30 | 99.18 | 88.43 | 8.63 | **9.76** |  |  |  |  |  |  |
|  |  |  | Analyst III | 85.81 | 82.75 | 73.56 | 82.53 | 104.70 | 85.87 | 11.48 | **13.37** |  |  |  |  |  |  |
|  |  |  | Mean of Analysts | 82.61 | 85.26 | 84.21 | 89.36 | 98.07 | **Mean (general)** | | |  |  |  |  |  |  |
|  |  |  | SD of Analysts | 6.56 | 10.92 | 9.81 | 8.06 | 7.25 | **87.90** | | |  |  |  |  |  |  |
|  |  |  | **RSD of Analysts (%)** | **7.94** | **12.81** | **11.65** | **9.02** | **7.39** |  |  |  |  |  |  |  |  |  |
|  |  | 1000 | Analyst I | 882.60 | 870.93 | 881.16 | 835.84 | 915.404 | 877.19 | 28.52 | **3.25** | 4778.29 | 4801.63 | -7.78 | 69.24 | 0.0771 |  |
|  |  |  | Analyst II | 1015.01 | 864.95 | 959.32 | 889.94 | 878.88 | 921.62 | 63.61 | **6.90** |  |  |  |  |  |  |
|  |  |  | Analyst III | 852.40 | 1031.24 | 891.43 | 756.87 | 952.30 | 896.85 | 103.36 | **11.52** |  |  |  |  |  |  |
|  |  |  | Mean of Analysts | 916.67 | 922.37 | 910.63 | 827.55 | 915.53 | **Mean (general)** | | |  |  |  |  |  |  |
|  |  |  | SD of Analysts | 86.49 | 94.33 | 42.47 | 66.92 | 36.71 | **898.55** | | |  |  |  |  |  |  |
|  |  |  | **RSD of Analysts (%)** | **9.44** | **10.23** | **4.66** | **8.09** | **4.01** |  |  |  |  |  |  |  |  |  |

| **S.10.** continuous… | | | | | | | | | | | | | | | | | |
| --- | --- | --- | --- | --- | --- | --- | --- | --- | --- | --- | --- | --- | --- | --- | --- | --- | --- |
| **No** | **VOCs** | ***Spike Conc. µg/L*** | **Analysts** | **Recoveries, µg/L** | | | | | **Intra Days Control** | | | **Sd^2^** | **Sr^2^** | **S_L_^2^** | **S_WR_** | **RSD_WR_** | **RSD_WRpool_** |
|  |  |  |  | **1st Day** | **2nd Day** | **3rd Day** | **4th Day** | **5th Day** | **Mean** | **SD** | **RSD %** |  |  |  |  |  |  |

| 28 | Styren | 100 | Analyst I | 73.64 | 89.27 | 75.59 | 83.68 | 91.39 | 82.71 | 7.94 | **9.60** | 40.69 | 54.23 | -4.51 | 7.05 | 0.0859 | **0.089** |
| --- | --- | --- | --- | --- | --- | --- | --- | --- | --- | --- | --- | --- | --- | --- | --- | --- | --- |
|  |  |  | Analyst II | 88.41 | 72.17 | 83.93 | 78.61 | 84.06 | 81.43 | 6.24 | **7.66** |  |  |  |  |  |  |
|  |  |  | Analyst III | 77.52 | 95.11 | 72.17 | 84.14 | 82.00 | 82.19 | 8.56 | **10.41** |  |  |  |  |  |  |
|  |  |  | Mean of Analysts | 79.85 | 85.52 | 77.23 | 82.15 | 85.81 | **Mean (general)** | | |  |  |  |  |  |  |
|  |  |  | SD of Analysts | 7.66 | 11.92 | 6.05 | 3.07 | 4.94 | **82.11** | | |  |  |  |  |  |  |
|  |  |  | **RSD of Analysts (%)** | **9.59** | **13.94** | **7.83** | **3.73** | **5.76** |  |  |  |  |  |  |  |  |  |
|  |  | 1000 | Analyst I | 922.60 | 896.09 | 973.10 | 770.41 | 830.79 | 878.60 | 79.31 | **9.03** | 4746.34 | 7209.16 | -820.94 | 79.93 | 0.0918 |  |
|  |  |  | Analyst II | 768.68 | 904.15 | 776.59 | 903.33 | 1020.13 | 874.57 | 104.53 | **11.95** |  |  |  |  |  |  |
|  |  |  | Analyst III | 916.59 | 812.31 | 904.15 | 752.16 | 904.45 | 857.93 | 72.48 | **8.45** |  |  |  |  |  |  |
|  |  |  | Mean of Analysts | 869.29 | 870.85 | 884.61 | 808.63 | 918.46 | **Mean (general)** | | |  |  |  |  |  |  |
|  |  |  | SD of Analysts | 87.19 | 50.86 | 99.70 | 82.51 | 95.44 | **870.37** | | |  |  |  |  |  |  |
|  |  |  | **RSD of Analysts (%)** | **10.03** | **5.84** | **11.27** | **10.20** | **10.39** |  |  |  |  |  |  |  |  |  |
| 29 | Tribromomethane | 100 | Analyst I | 87.76 | 78.87 | 102.21 | 73.45 | 82.38 | 84.94 | 10.97 | **12.92** | 114.17 | 33.10 | 27.02 | 7.75 | 0.0906 | **0.090** |
|  |  |  | Analyst II | 81.59 | 88.59 | 87.04 | 80.67 | 94.91 | 86.56 | 5.78 | **6.68** |  |  |  |  |  |  |
|  |  |  | Analyst III | 89.52 | 82.43 | 88.60 | 75.06 | 90.09 | 85.14 | 6.41 | **7.53** |  |  |  |  |  |  |
|  |  |  | Mean of Analysts | 86.29 | 83.30 | 92.62 | 76.40 | 89.13 | **Mean (general)** | | |  |  |  |  |  |  |
|  |  |  | SD of Analysts | 4.17 | 4.92 | 8.35 | 3.79 | 6.32 | **85.55** | | |  |  |  |  |  |  |
|  |  |  | **RSD of Analysts (%)** | **4.83** | **5.90** | **9.01** | **4.96** | **7.09** |  |  |  |  |  |  |  |  |  |
|  |  | 1000 | Analyst I | 882.60 | 896.09 | 973.10 | 770.41 | 830.79 | 870.60 | 75.70 | **8.70** | 5112.76 | 6377.72 | -421.66 | 77.18 | 0.0886 |  |
|  |  |  | Analyst II | 768.68 | 891.43 | 776.59 | 903.33 | 1020.13 | 872.03 | 103.78 | **11.90** |  |  |  |  |  |  |
|  |  |  | Analyst III | 916.59 | 877.12 | 900.34 | 752.16 | 904.45 | 870.13 | 67.48 | **7.76** |  |  |  |  |  |  |
|  |  |  | Mean of Analysts | 855.96 | 888.21 | 883.34 | 808.63 | 918.46 | **Mean (general)** | | |  |  |  |  |  |  |
|  |  |  | SD of Analysts | 77.47 | 9.89 | 99.35 | 82.51 | 95.44 | **870.92** | | |  |  |  |  |  |  |
|  |  |  | **RSD of Analysts (%)** | **9.05** | **1.11** | **11.25** | **10.20** | **10.39** |  |  |  |  |  |  |  |  |  |
| 30 | Isopropylbenzene | 100 | Analyst I | 106.70 | 98.67 | 91.47 | 93.21 | 87.18 | 95.45 | 7.52 | **7.88** | 59.24 | 87.18 | -9.31 | 8.82 | 0.0936 | **0.087** |
|  |  |  | Analyst II | 91.75 | 109.90 | 88.79 | 88.16 | 82.77 | 92.27 | 10.37 | **11.24** |  |  |  |  |  |  |
|  |  |  | Analyst III | 84.27 | 89.18 | 110.96 | 98.55 | 92.65 | 95.12 | 10.27 | **10.80** |  |  |  |  |  |  |
|  |  |  | Mean of Analysts | 94.24 | 99.25 | 97.07 | 93.31 | 87.53 | **Mean (general)** | | |  |  |  |  |  |  |
|  |  |  | SD of Analysts | 11.42 | 10.37 | 12.10 | 5.20 | 4.95 | **94.28** | | |  |  |  |  |  |  |
|  |  |  | **RSD of Analysts (%)** | **12.12** | **10.45** | **12.47** | **5.57** | **5.65** |  |  |  |  |  |  |  |  |  |
|  |  | 1000 | Analyst I | 892.60 | 984.66 | 812.47 | 865.20 | 857.45 | 882.47 | 63.97 | **7.25** | 7735.94 | 4247.88 | 1162.69 | 73.56 | 0.0807 |  |
|  |  |  | Analyst II | 851.56 | 928.30 | 903.97 | 1035.64 | 905.51 | 925.00 | 67.95 | **7.35** |  |  |  |  |  |  |
|  |  |  | Analyst III | 810.62 | 1042.54 | 919.38 | 876.84 | 981.16 | 926.11 | 90.00 | **9.72** |  |  |  |  |  |  |
|  |  |  | Mean of Analysts | 851.59 | 985.17 | 878.60 | 925.89 | 914.71 | **Mean (general)** | | |  |  |  |  |  |  |
|  |  |  | SD of Analysts | 40.99 | 57.12 | 57.79 | 95.22 | 62.37 | **911.19** | | |  |  |  |  |  |  |
|  |  |  | **RSD of Analysts (%)** | **4.81** | **5.80** | **6.58** | **10.28** | **6.82** |  |  |  |  |  |  |  |  |  |

| **S.10.** continuous… | | | | | | | | | | | | | | | | | |
| --- | --- | --- | --- | --- | --- | --- | --- | --- | --- | --- | --- | --- | --- | --- | --- | --- | --- |
| **No** | **VOCs** | ***Spike Conc. µg/L*** | **Analysts** | **Recoveries, µg/L** | | | | | **Intra Days Control** | | | **Sd^2^** | **Sr^2^** | **S_L_^2^** | **S_WR_** | **RSD_WR_** | **RSD_WRpool_** |
|  |  |  |  | **1st Day** | **2nd Day** | **3rd Day** | **4th Day** | **5th Day** | **Mean** | **SD** | **RSD %** |  |  |  |  |  |  |

| 31 | 1,1,2,2-tetrachloroethane | 100 | Analyst I | 83.64 | 98.67 | 86.22 | 83.32 | 87.18 | 87.81 | 6.29 | **7.17** | 49.82 | 42.20 | 2.54 | 6.69 | 0.0780 | **0.077** |
| --- | --- | --- | --- | --- | --- | --- | --- | --- | --- | --- | --- | --- | --- | --- | --- | --- | --- |
|  |  |  | Analyst II | 80.22 | 81.97 | 88.79 | 77.92 | 82.77 | 82.33 | 4.06 | **4.93** |  |  |  |  |  |  |
|  |  |  | Analyst III | 97.51 | 89.18 | 80.33 | 76.24 | 92.65 | 87.18 | 8.76 | **10.05** |  |  |  |  |  |  |
|  |  |  | Mean of Analysts | 87.12 | 89.94 | 85.11 | 79.16 | 87.53 | **Mean (general)** | | |  |  |  |  |  |  |
|  |  |  | SD of Analysts | 9.16 | 8.38 | 4.34 | 3.70 | 4.95 | **85.77** | | |  |  |  |  |  |  |
|  |  |  | RSD of Analysts (%) | **10.51** | **9.32** | **5.10** | **4.67** | **5.65** |  |  |  |  |  |  |  |  |  |
|  |  | 1000 | Analyst I | 937.87 | 984.66 | 812.47 | 865.20 | 857.45 | 891.53 | 68.78 | **7.72** | 5080.73 | 4504.12 | 192.20 | 68.53 | 0.0765 |  |
|  |  |  | Analyst II | 1001.84 | 919.11 | 903.97 | 889.94 | 905.51 | 924.07 | 44.68 | **4.84** |  |  |  |  |  |  |
|  |  |  | Analyst III | 810.62 | 918.21 | 900.73 | 756.87 | 981.16 | 873.52 | 89.28 | **10.22** |  |  |  |  |  |  |
|  |  |  | Mean of Analysts | 916.78 | 940.66 | 872.39 | 837.34 | 914.71 | **Mean (general)** | | |  |  |  |  |  |  |
|  |  |  | SD of Analysts | 97.34 | 38.10 | 51.92 | 70.77 | 62.37 | **896.37** | | |  |  |  |  |  |  |
|  |  |  | **RSD of Analysts (%)** | **10.62** | **4.05** | **5.95** | **8.45** | **6.82** |  |  |  |  |  |  |  |  |  |
| 32 | Bromobenzene | 100 | Analyst I | 93.16 | 95.71 | 84.21 | 83.68 | 86.30 | 88.61 | 5.48 | **6.18** | 87.37 | 36.45 | 16.97 | 7.31 | 0.0830 | **0.101** |
|  |  |  | Analyst II | 75.95 | 91.30 | 92.61 | 77.32 | 85.74 | 84.58 | 7.72 | **9.12** |  |  |  |  |  |  |
|  |  |  | Analyst III | 82.57 | 99.76 | 89.47 | 84.92 | 98.78 | 91.10 | 7.87 | **8.64** |  |  |  |  |  |  |
|  |  |  | Mean of Analysts | 83.89 | 95.59 | 88.76 | 81.97 | 90.27 | **Mean (general)** | | |  |  |  |  |  |  |
|  |  |  | SD of Analysts | 8.68 | 4.23 | 4.25 | 4.08 | 7.37 | **88.10** | | |  |  |  |  |  |  |
|  |  |  | **RSD of Analysts (%)** | **10.35** | **4.43** | **4.78** | **4.97** | **8.17** |  |  |  |  |  |  |  |  |  |
|  |  | 1000 | Analyst I | 1082.60 | 960.92 | 870.93 | 752.82 | 1051.04 | 943.66 | 134.95 | **14.30** | 20535.32 | 7153.29 | 4460.68 | 107.77 | 0.1162 |  |
|  |  |  | Analyst II | 992.38 | 1060.95 | 829.03 | 830.71 | 910.24 | 924.66 | 101.66 | **10.99** |  |  |  |  |  |  |
|  |  |  | Analyst III | 872.65 | 914.32 | 1039.73 | 781.99 | 962.04 | 914.15 | 96.50 | **10.56** |  |  |  |  |  |  |
|  |  |  | Mean of Analysts | 982.54 | 978.73 | 913.23 | 788.51 | 974.44 | **Mean (general)** | | |  |  |  |  |  |  |
|  |  |  | SD of Analysts | 105.32 | 74.92 | 111.54 | 39.35 | 71.22 | **927.49** | | |  |  |  |  |  |  |
|  |  |  | **RSD of Analysts (%)** | **10.72** | **7.65** | **12.21** | **4.99** | **7.31** |  |  |  |  |  |  |  |  |  |
| 33 | n-propylbenzene | 100 | Analyst I | 81.16 | 91.11 | 92.48 | 79.01 | 93.76 | 87.51 | 6.88 | **7.86** | 63.79 | 46.56 | 5.75 | 7.23 | 0.0831 | **0.090** |
|  |  |  | Analyst II | 91.69 | 80.35 | 82.25 | 79.28 | 97.53 | 86.22 | 8.00 | **9.28** |  |  |  |  |  |  |
|  |  |  | Analyst III | 78.62 | 100.09 | 81.97 | 88.58 | 88.30 | 87.51 | 8.21 | **9.38** |  |  |  |  |  |  |
|  |  |  | Mean of Analysts | 83.82 | 90.52 | 85.57 | 82.29 | 93.20 | **Mean (general)** | | |  |  |  |  |  |  |
|  |  |  | SD of Analysts | 6.93 | 9.88 | 5.99 | 5.45 | 4.64 | **87.08** | | |  |  |  |  |  |  |
|  |  |  | **RSD of Analysts (%)** | **8.27** | **10.92** | **7.00** | **6.62** | **4.98** |  |  |  |  |  |  |  |  |  |
|  |  | 1000 | Analyst I | 927.87 | 942.15 | 879.07 | 901.07 | 903.32 | 910.70 | 24.65 | **2.71** | 11598.99 | 5806.84 | 1930.72 | 87.96 | 0.0960 |  |
|  |  |  | Analyst II | 826.00 | 937.15 | 833.23 | 861.51 | 1055.09 | 902.60 | 95.94 | **10.63** |  |  |  |  |  |  |
|  |  |  | Analyst III | 929.18 | 1135.93 | 918.59 | 791.89 | 900.92 | 935.30 | 124.82 | **13.35** |  |  |  |  |  |  |
|  |  |  | Mean of Analysts | 894.35 | 1005.07 | 876.97 | 851.49 | 953.11 | **Mean (general)** | | |  |  |  |  |  |  |
|  |  |  | SD of Analysts | 59.20 | 113.35 | 42.72 | 55.28 | 88.33 | **916.20** | | |  |  |  |  |  |  |
|  |  |  | **RSD of Analysts (%)** | **6.62** | **11.28** | **4.87** | **6.49** | **9.27** |  |  |  |  |  |  |  |  |  |

| **S.10.** continuous… | | | | | | | | | | | | | | | | | |
| --- | --- | --- | --- | --- | --- | --- | --- | --- | --- | --- | --- | --- | --- | --- | --- | --- | --- |
| **No** | **VOCs** | ***Spike Conc. µg/L*** | **Analysts** | **Recoveries, µg/L** | | | | | **Intra Days Control** | | | **Sd^2^** | **Sr^2^** | **S_L_^2^** | **S_WR_** | **RSD_WR_** | **RSD_WRpool_** |
|  |  |  |  | **1st Day** | **2nd Day** | **3rd Day** | **4th Day** | **5th Day** | **Mean** | **SD** | **RSD %** |  |  |  |  |  |  |

| 34 | 2-chlorotoluene | 100 | Analyst I | 83.16 | 88.93 | 78.41 | 80.02 | 94.93 | 85.09 | 6.81 | **8.00** | 50.35 | 30.03 | 6.77 | 6,07 | 0.0710 | **0.085** |
| --- | --- | --- | --- | --- | --- | --- | --- | --- | --- | --- | --- | --- | --- | --- | --- | --- | --- |
|  |  |  | Analyst II | 78.53 | 81.50 | 89.77 | 74.88 | 90.15 | 82.97 | 6.80 | **8.20** |  |  |  |  |  |  |
|  |  |  | Analyst III | 90.58 | 93.72 | 84.82 | 85.62 | 87.56 | 88.46 | 3.68 | **4.16** |  |  |  |  |  |  |
|  |  |  | Mean of Analysts | 84.09 | 88.05 | 84.33 | 80.18 | 90.88 | **Mean (general)** | | |  |  |  |  |  |  |
|  |  |  | SD of Analysts | 6.08 | 6.16 | 5.70 | 5.37 | 3.74 | **85.51** | | |  |  |  |  |  |  |
|  |  |  | **RSD of Analysts (%)** | **7.23** | **6.99** | **6.75** | **6.70** | **4.11** |  |  |  |  |  |  |  |  |  |
|  |  | 1000 | Analyst I | 896.40 | 942.15 | 879.07 | 901.07 | 903.32 | 904.40 | 23.14 | **2.56** | 11827.20 | 5779.93 | 2015.76 | 88,29 | 0.0970 |  |
|  |  |  | Analyst II | 826.00 | 914.33 | 833.23 | 861.51 | 1055.09 | 898.03 | 94.41 | **10.51** |  |  |  |  |  |  |
|  |  |  | Analyst III | 929.18 | 1135.93 | 878.47 | 791.89 | 900.92 | 927.28 | 127.42 | **13.74** |  |  |  |  |  |  |
|  |  |  | Mean of Analysts | 883.86 | 997.47 | 863.59 | 851.49 | 953.11 | **Mean (general)** | | |  |  |  |  |  |  |
|  |  |  | SD of Analysts | 52.72 | 120.71 | 26.29 | 55.28 | 88.33 | **909.90** | | |  |  |  |  |  |  |
|  |  |  | **RSD of Analysts (%)** | **5.96** | **12.10** | **3.04** | **6.49** | **9.27** |  |  |  |  |  |  |  |  |  |
| 35 | 1,3,5-trimethylbenzene | 100 | Analyst I | 110.80 | 98.93 | 88.41 | 90.23 | 94.93 | 96.66 | 8.91 | **9.22** | 61.54 | 113.54 | -17.33 | 9,81 | 0.1017 | **0.088** |
|  |  |  | Analyst II | 92.54 | 115.24 | 89.77 | 104.23 | 90.15 | 98.39 | 11.12 | **11.30** |  |  |  |  |  |  |
|  |  |  | Analyst III | 90.58 | 93.72 | 114.13 | 85.62 | 87.56 | 94.32 | 11.49 | **12.18** |  |  |  |  |  |  |
|  |  |  | Mean of Analysts | 97.97 | 102.63 | 97.44 | 93.36 | 90.88 | **Mean (general)** | | |  |  |  |  |  |  |
|  |  |  | SD of Analysts | 11.15 | 11.22 | 14.47 | 9.69 | 3.74 | **96.46** | | |  |  |  |  |  |  |
|  |  |  | **RSD of Analysts (%)** | **11.38** | **10.94** | **14.85** | **10.38** | **4.11** |  |  |  |  |  |  |  |  |  |
|  |  | 1000 | Analyst I | 891.40 | 980.13 | 844.80 | 782.93 | 998.49 | 899.55 | 90.76 | **10.09** | 6320.60 | 3060.02 | 1086.86 | 64,40 | 0.0716 |  |
|  |  |  | Analyst II | 911.96 | 918.14 | 914.24 | 856.02 | 894.28 | 898.93 | 25.67 | **2.86** |  |  |  |  |  |  |
|  |  |  | Analyst III | 813.42 | 860.24 | 927.06 | 895.97 | 1002.34 | 899.80 | 71.24 | **7.92** |  |  |  |  |  |  |
|  |  |  | Mean of Analysts | 872.26 | 919.51 | 895.36 | 844.97 | 965.04 | **Mean (general)** | | |  |  |  |  |  |  |
|  |  |  | SD of Analysts | 51.99 | 59.96 | 44.26 | 57.32 | 61.30 | **899.43** | | |  |  |  |  |  |  |
|  |  |  | **RSD of Analysts (%)** | **5.96** | **6.52** | **4.94** | **6.78** | **6.35** |  |  |  |  |  |  |  |  |  |
| 36 | 4-chlorotoluene | 100 | Analyst I | 82.06 | 95.75 | 80.98 | 79.86 | 90.65 | 85.86 | 6.98 | **8.13** | 61.96 | 29.53 | 10.81 | 6,35 | 0.0731 | **0.081** |
|  |  |  | Analyst II | 95.20 | 83.70 | 84.91 | 81.34 | 90.19 | 87.07 | 5.58 | **6.41** |  |  |  |  |  |  |
|  |  |  | Analyst III | 88.08 | 91.91 | 95.22 | 75.83 | 86.81 | 87.57 | 7.35 | **8.39** |  |  |  |  |  |  |
|  |  |  | Mean of Analysts | 88.45 | 90.45 | 87.04 | 79.01 | 89.21 | **Mean (general)** | | |  |  |  |  |  |  |
|  |  |  | SD of Analysts | 6.57 | 6.15 | 7.35 | 2.85 | 2.10 | **86.83** | | |  |  |  |  |  |  |
|  |  |  | **RSD of Analysts (%)** | **7.43** | **6.80** | **8.45** | **3.60** | **2.35** |  |  |  |  |  |  |  |  |  |
|  |  | 1000 | Analyst I | 842.60 | 980.13 | 844.80 | 782.93 | 998.49 | 889.79 | 94.40 | **10.61** | 9804.19 | 4690.64 | 1704.52 | 79,97 | 0.0890 |  |
|  |  |  | Analyst II | 911.96 | 834.17 | 914.24 | 856.02 | 894.28 | 882.14 | 35.53 | **4.03** |  |  |  |  |  |  |
|  |  |  | Analyst III | 813.42 | 1053.93 | 859.45 | 895.97 | 1002.34 | 925.02 | 100.24 | **10.84** |  |  |  |  |  |  |
|  |  |  | Mean of Analysts | 855.99 | 956.08 | 872.83 | 844.97 | 965.04 | **Mean (general)** | | |  |  |  |  |  |  |
|  |  |  | SD of Analysts | 50.62 | 111.84 | 36.60 | 57.32 | 61.30 | **898.98** | | |  |  |  |  |  |  |
|  |  |  | **RSD of Analysts (%)** | **5.91** | **11.70** | **4.19** | **6.78** | **6.35** |  |  |  |  |  |  |  |  |  |

| **S.10.** continuous… | | | | | | | | | | | | | | | | | |
| --- | --- | --- | --- | --- | --- | --- | --- | --- | --- | --- | --- | --- | --- | --- | --- | --- | --- |
| **No** | **VOCs** | ***Spike Conc. µg/L*** | **Analysts** | **Recoveries, µg/L** | | | | | **Intra Days Control** | | | **Sd^2^** | **Sr^2^** | **S_L_^2^** | **S_WR_** | **RSD_WR_** | **RSD_WRpool_** |
|  |  |  |  | **1st Day** | **2nd Day** | **3rd Day** | **4th Day** | **5th Day** | **Mean** | **SD** | **RSD %** |  |  |  |  |  |  |

| 37 | Tert-butylbenzene | 100 | Analyst I | 95.16 | 95.30 | 87.53 | 77.32 | 85.74 | 88.21 | 7.48 | **8.48** | 155.21 | 16.08 | 46.38 | 7.90 | 0.0902 | **0.088** |
| --- | --- | --- | --- | --- | --- | --- | --- | --- | --- | --- | --- | --- | --- | --- | --- | --- | --- |
|  |  |  | Analyst II | 95.26 | 94.21 | 85.88 | 79.93 | 83.64 | 87.78 | 6.70 | **7.64** |  |  |  |  |  |  |
|  |  |  | Analyst III | 88.04 | 91.15 | 93.26 | 69.78 | 91.33 | 86.71 | 9.65 | **11.13** |  |  |  |  |  |  |
|  |  |  | Mean of Analysts | 92.82 | 93.55 | 88.89 | 75.68 | 86.90 | **Mean (general)** | | |  |  |  |  |  |  |
|  |  |  | SD of Analysts | 4.14 | 2.15 | 3.87 | 5.27 | 3.98 | **87.57** | | |  |  |  |  |  |  |
|  |  |  | **RSD of Analysts (%)** | **4.46** | **2.30** | **4.36** | **6.97** | **4.58** |  |  |  |  |  |  |  |  |  |
|  |  | 1000 | Analyst I | 902.60 | 1055.61 | 940.13 | 845.44 | 896.46 | 928.05 | 78.88 | **8.50** | 7423.13 | 5160.25 | 754.29 | 76.91 | 0.0855 |  |
|  |  |  | Analyst II | 826.83 | 884.55 | 921.34 | 719.03 | 971.35 | 864.62 | 96.99 | **11.22** |  |  |  |  |  |  |
|  |  |  | Analyst III | 974.22 | 919.58 | 893.57 | 890.09 | 849.40 | 905.37 | 45.96 | **5.08** |  |  |  |  |  |  |
|  |  |  | Mean of Analysts | 901.22 | 953.25 | 918.35 | 818.19 | 905.73 | **Mean (general)** | | |  |  |  |  |  |  |
|  |  |  | SD of Analysts | 73.70 | 90.36 | 23.42 | 88.73 | 61.50 | **899.35** | | |  |  |  |  |  |  |
|  |  |  | **RSD of Analysts (%)** | **8.18** | **9.48** | **2.55** | **10.84** | **6.79** |  |  |  |  |  |  |  |  |  |
| 38 | 1,2,4-trimethylbenzene | 100 | Analyst I | 80.16 | 84.56 | 96.32 | 73.45 | 84.88 | 83.87 | 8.35 | **9.95** | 90.67 | 36.77 | 17.97 | 7.40 | 0.0875 | **0.077** |
|  |  |  | Analyst II | 88.59 | 79.36 | 82.02 | 80.67 | 97.79 | 85.69 | 7.64 | **8.92** |  |  |  |  |  |  |
|  |  |  | Analyst III | 85.13 | 88.38 | 78.56 | 75.06 | 92.82 | 83.99 | 7.21 | **8.58** |  |  |  |  |  |  |
|  |  |  | Mean of Analysts | 84.63 | 84.10 | 85.63 | 76.40 | 91.83 | **Mean (general)** | | |  |  |  |  |  |  |
|  |  |  | SD of Analysts | 4.24 | 4.53 | 9.41 | 3.79 | 6.51 | **84.52** | | |  |  |  |  |  |  |
|  |  |  | **RSD of Analysts (%)** | **5.01** | **5.38** | **10.99** | **4.96** | **7.09** |  |  |  |  |  |  |  |  |  |
|  |  | 1000 | Analyst I | 902.60 | 864.97 | 877.35 | 931.09 | 871.15 | 889.43 | 27.33 | **3.07** | 1878.92 | 4047.73 | -722.94 | 57.66 | 0.0651 |  |
|  |  |  | Analyst II | 843.65 | 884.55 | 935.67 | 810.96 | 839.18 | 862.80 | 48.47 | **5.62** |  |  |  |  |  |  |
|  |  |  | Analyst III | 918.01 | 923.96 | 858.01 | 792.44 | 1030.43 | 904.57 | 88.23 | **9.75** |  |  |  |  |  |  |
|  |  |  | Mean of Analysts | 888.09 | 891.16 | 890.35 | 844.83 | 913.59 | **Mean (general)** | | |  |  |  |  |  |  |
|  |  |  | SD of Analysts | 39.25 | 30.04 | 40.43 | 75.27 | 102.44 | **885.60** | | |  |  |  |  |  |  |
|  |  |  | **RSD of Analysts (%)** | **4.42** | **3.37** | **4.54** | **8.91** | **11.21** |  |  |  |  |  |  |  |  |  |
| 39 | Sec-butylbenzene | 100 | Analyst I | 84.64 | 90.31 | 76.57 | 84.63 | 92.41 | 85.71 | 6.16 | **7.19** | 28.22 | 26.46 | 0.59 | 5.20 | 0.0610 | **0.087** |
|  |  |  | Analyst II | 89.38 | 82.95 | 84.91 | 79.56 | 85.08 | 84.37 | 3.57 | **4.23** |  |  |  |  |  |  |
|  |  |  | Analyst III | 78.49 | 96.15 | 85.49 | 85.09 | 83.02 | 85.65 | 6.50 | **7.58** |  |  |  |  |  |  |
|  |  |  | Mean of Analysts | 84.17 | 89.80 | 82.32 | 83.10 | 86.83 | **Mean (general)** | | |  |  |  |  |  |  |
|  |  |  | SD of Analysts | 5.46 | 6.62 | 4.99 | 3.07 | 4.94 | **85.24** | | |  |  |  |  |  |  |
|  |  |  | **RSD of Analysts (%)** | **6.49** | **7.37** | **6.06** | **3.69** | **5.69** |  |  |  |  |  |  |  |  |  |
|  |  | 1000 | Analyst I | 892.60 | 931.93 | 953.64 | 731.89 | 847.41 | 871.49 | 87.91 | **10.09** | 11789.02 | 6796.83 | 1664.06 | 91.98 | 0.1069 |  |
|  |  |  | Analyst II | 745.62 | 901.53 | 761.06 | 858.16 | 1040.53 | 861.38 | 119.57 | **13.88** |  |  |  |  |  |  |
|  |  |  | Analyst III | 889.10 | 844.80 | 874.75 | 714.55 | 922.54 | 849.15 | 80.27 | **9.45** |  |  |  |  |  |  |
|  |  |  | Mean of Analysts | 842.44 | 892.75 | 863.15 | 768.20 | 936.83 | **Mean (general)** | | |  |  |  |  |  |  |
|  |  |  | SD of Analysts | 83.87 | 44.22 | 96.81 | 78.39 | 97.35 | **860.67** | | |  |  |  |  |  |  |
|  |  |  | **RSD of Analysts (%)** | **9.96** | **4.95** | **11.22** | **10.20** | **10.39** |  |  |  |  |  |  |  |  |  |

| **S.10.** continuous… | | | | | | | | | | | | | | | | | |
| --- | --- | --- | --- | --- | --- | --- | --- | --- | --- | --- | --- | --- | --- | --- | --- | --- | --- |
| **No** | **VOCs** | ***Spike Conc. µg/L*** | **Analysts** | **Recoveries, µg/L** | | | | | **Intra Days Control** | | | **Sd^2^** | **Sr^2^** | **S_L_^2^** | **S_WR_** | **RSD_WR_** | **RSD_WRpool_** |
|  |  |  |  | **1st Day** | **2nd Day** | **3rd Day** | **4th Day** | **5th Day** | **Mean** | **SD** | **RSD %** |  |  |  |  |  |  |

| 40 | 1,3-dichlorobenzene | 100 | Analyst I | 83.16 | 99.54 | 82.53 | 79.50 | 88.03 | 86.55 | 7.88 | **9.10** | 190.67 | 43.90 | 48.92 | 9.63 | 0.1121 | **0.112** |
| --- | --- | --- | --- | --- | --- | --- | --- | --- | --- | --- | --- | --- | --- | --- | --- | --- | --- |
|  |  |  | Analyst II | 73.67 | 84.82 | 90.76 | 73.45 | 87.45 | 82.03 | 8.01 | **9.77** |  |  |  |  |  |  |
|  |  |  | Analyst III | 80.09 | 103.75 | 81.50 | 80.67 | 100.75 | 89.35 | 11.83 | **13.24** |  |  |  |  |  |  |
|  |  |  | Mean of Analysts | 78.98 | 96.04 | 84.93 | 77.87 | 92.08 | **Mean (general)** | | |  |  |  |  |  |  |
|  |  |  | SD of Analysts | 4.84 | 9.94 | 5.08 | 3.87 | 7.52 | **85.98** | | |  |  |  |  |  |  |
|  |  |  | **RSD of Analysts (%)** | **6.13** | **10.35** | **5.98** | **4.97** | **8.17** |  |  |  |  |  |  |  |  |  |
|  |  | 1000 | Analyst I | 812.60 | 999.35 | 987.34 | 843.98 | 1072.06 | 943.07 | 110.24 | **11.69** | 16127.34 | 6919.40 | 3069.31 | 99.94 | 0.1123 |  |
|  |  |  | Analyst II | 962.61 | 796.35 | 812.45 | 789.17 | 928.44 | 857.80 | 81.42 | **9.49** |  |  |  |  |  |  |
|  |  |  | Analyst III | 846.47 | 950.89 | 828.85 | 742.89 | 981.28 | 870.08 | 96.61 | **11.10** |  |  |  |  |  |  |
|  |  |  | Mean of Analysts | 873.89 | 915.53 | 876.21 | 792.01 | 993.93 | **Mean (general)** | | |  |  |  |  |  |  |
|  |  |  | SD of Analysts | 78.67 | 106.02 | 96.59 | 50.61 | 72.64 | **890.32** | | |  |  |  |  |  |  |
|  |  |  | **RSD of Analysts (%)** | **9.00** | **11.58** | **11.02** | **6.39** | **7.31** |  |  |  |  |  |  |  |  |  |
| 41 | 4-isopropyltolune | 100 | Analyst I | 88.16 | 94.76 | 90.63 | 75.06 | 95.64 | 88.85 | 8.29 | **9.33** | 160.69 | 40.39 | 40.10 | 8.97 | 0.1019 | **0.108** |
|  |  |  | Analyst II | 88.94 | 87.28 | 80.60 | 75.31 | 99.48 | 86.32 | 9.15 | **10.60** |  |  |  |  |  |  |
|  |  |  | Analyst III | 76.26 | 104.09 | 89.92 | 84.15 | 90.06 | 88.90 | 10.19 | **11.47** |  |  |  |  |  |  |
|  |  |  | Mean of Analysts | 84.45 | 95.38 | 87.05 | 78.18 | 95.06 | **Mean (general)** | | |  |  |  |  |  |  |
|  |  |  | SD of Analysts | 7.11 | 8.42 | 5.60 | 5.18 | 4.73 | **88.02** | | |  |  |  |  |  |  |
|  |  |  | **RSD of Analysts (%)** | **8.41** | **8.83** | **6.43** | **6.62** | **4.98** |  |  |  |  |  |  |  |  |  |
|  |  | 1000 | Analyst I | 892.60 | 979.84 | 861.49 | 856.02 | 921.38 | 902.27 | 50.69 | **5.62** | 19246.16 | 6588.96 | 4219.07 | 103.96 | 0.1139 |  |
|  |  |  | Analyst II | 801.22 | 910.45 | 816.57 | 818.43 | 1076.19 | 884.57 | 115.47 | **13.05** |  |  |  |  |  |  |
|  |  |  | Analyst III | 901.30 | 1181.37 | 883.67 | 873.08 | 918.94 | 951.67 | 129.58 | **13.62** |  |  |  |  |  |  |
|  |  |  | Mean of Analysts | 865.04 | 1023.88 | 853.91 | 849.18 | 972.17 | **Mean (general)** | | |  |  |  |  |  |  |
|  |  |  | SD of Analysts | 55.44 | 140.73 | 34.19 | 27.96 | 90.09 | **912.84** | | |  |  |  |  |  |  |
|  |  |  | **RSD of Analysts (%)** | **6.41** | **13.74** | **4.00** | **3.29** | **9.27** |  |  |  |  |  |  |  |  |  |
| 42 | 1,4-dichlorobenzene | 100 | Analyst I | 84.16 | 102.89 | 103.71 | 85.72 | 96.82 | 94.66 | 9.28 | **9.80** | 44.00 | 61.91 | -5.97 | 7.48 | 0.0824 | **0.095** |
|  |  |  | Analyst II | 89.76 | 83.32 | 87.97 | 99.02 | 91.95 | 90.41 | 5.77 | **6.38** |  |  |  |  |  |  |
|  |  |  | Analyst III | 81.36 | 97.47 | 86.69 | 81.34 | 89.31 | 87.23 | 6.68 | **7.66** |  |  |  |  |  |  |
|  |  |  | Mean of Analysts | 85.10 | 94.56 | 92.79 | 88.69 | 92.70 | **Mean (general)** | | |  |  |  |  |  |  |
|  |  |  | SD of Analysts | 4.28 | 10.10 | 9.48 | 9.21 | 3.81 | **90.77** | | |  |  |  |  |  |  |
|  |  |  | **RSD of Analysts (%)** | **5.03** | **10.68** | **10.22** | **10.38** | **4.11** |  |  |  |  |  |  |  |  |  |
|  |  | 1000 | Analyst I | 801.60 | 1019.34 | 827.91 | 743.79 | 1018.46 | 882.22 | 128.43 | **14.56** | 16795.95 | 4385.00 | 4136.98 | 92.31 | 0.1058 |  |
|  |  |  | Analyst II | 884.60 | 825.65 | 895.95 | 813.22 | 912.17 | 866.32 | 44.13 | **5.09** |  |  |  |  |  |  |
|  |  |  | Analyst III | 789.01 | 894.65 | 793.58 | 851.17 | 1022.38 | 870.16 | 95.63 | **10.99** |  |  |  |  |  |  |
|  |  |  | Mean of Analysts | 825.07 | 913.21 | 839.15 | 802.73 | 984.34 | **Mean (general)** | | |  |  |  |  |  |  |
|  |  |  | SD of Analysts | 51.94 | 98.17 | 52.10 | 54.46 | 62.53 | **872.90** | | |  |  |  |  |  |  |
|  |  |  | **RSD of Analysts (%)** | **6.29** | **10.75** | **6.21** | **6.78** | **6.35** |  |  |  |  |  |  |  |  |  |

| **S.10.** continuous… | | | | | | | | | | | | | | | | | |
| --- | --- | --- | --- | --- | --- | --- | --- | --- | --- | --- | --- | --- | --- | --- | --- | --- | --- |
| **No** | **VOCs** | ***Spike Conc. µg/L*** | **Analysts** | **Recoveries, µg/L** | | | | | **Intra Days Control** | | | **Sd^2^** | **Sr^2^** | **S_L_^2^** | **S_WR_** | **RSD_WR_** | **RSD_WRpool_** |
|  |  |  |  | **1st Day** | **2nd Day** | **3rd Day** | **4th Day** | **5th Day** | **Mean** | **SD** | **RSD %** |  |  |  |  |  |  |

| 43 | n-butylbenzene | 100 | Analyst I | 100.80 | 99.58 | 79.36 | 105.46 | 92.46 | 95.53 | 10.17 | **10.64** | 24.91 | 73.80 | -16.30 | 7.58 | 0.0826 | **0.098** |
| --- | --- | --- | --- | --- | --- | --- | --- | --- | --- | --- | --- | --- | --- | --- | --- | --- | --- |
|  |  |  | Analyst II | 92.34 | 98.79 | 83.21 | 82.45 | 91.99 | 89.76 | 6.88 | **7.67** |  |  |  |  |  |  |
|  |  |  | Analyst III | 85.44 | 87.65 | 99.80 | 88.35 | 88.54 | 89.96 | 5.64 | **6.27** |  |  |  |  |  |  |
|  |  |  | Mean of Analysts | 92.86 | 95.34 | 87.46 | 92.09 | 91.00 | **Mean (general)** | | |  |  |  |  |  |  |
|  |  |  | SD of Analysts | 7.69 | 6.67 | 10.86 | 11.95 | 2.14 | **91.75** | | |  |  |  |  |  |  |
|  |  |  | **RSD of Analysts (%)** | **8.29** | **7.00** | **12.41** | **12.98** | **2.35** |  |  |  |  |  |  |  |  |  |
|  |  | 1000 | Analyst I | 871.40 | 1019.34 | 827.91 | 743.79 | 1018.46 | 896.18 | 121.06 | **13.51** | 19987.39 | 5691.68 | 4765.24 | 102.26 | 0.1120 |  |
|  |  |  | Analyst II | 1016.42 | 862.69 | 895.95 | 813.22 | 912.17 | 900.09 | 75.24 | **8.36** |  |  |  |  |  |  |
|  |  |  | Analyst III | 889.27 | 1096.09 | 853.97 | 851.17 | 1022.38 | 942.58 | 110.66 | **11.74** |  |  |  |  |  |  |
|  |  |  | Mean of Analysts | 925.70 | 992.70 | 859.28 | 802.73 | 984.34 | **Mean (general)** | | |  |  |  |  |  |  |
|  |  |  | SD of Analysts | 79.08 | 118.96 | 34.33 | 54.46 | 62.53 | **912.95** | | |  |  |  |  |  |  |
|  |  |  | **RSD of Analysts (%)** | **8.54** | **11.98** | **4.00** | **6.78** | **6.35** |  |  |  |  |  |  |  |  |  |
| 44 | 1,2-dichlorobenzene | 100 | Analyst I | 86.16 | 87.94 | 94.39 | 69.78 | 86.58 | 84.97 | 9.11 | **10.73** | 199.40 | 25.08 | 58.11 | 9.12 | 0.1060 | **0.117** |
|  |  |  | Analyst II | 85.93 | 89.61 | 80.38 | 76.64 | 99.75 | 86.46 | 8.95 | **10.35** |  |  |  |  |  |  |
|  |  |  | Analyst III | 82.58 | 91.92 | 93.19 | 71.31 | 94.68 | 86.73 | 9.83 | **11.34** |  |  |  |  |  |  |
|  |  |  | Mean of Analysts | 84.89 | 89.82 | 89.32 | 72.58 | 93.67 | **Mean (general)** | | |  |  |  |  |  |  |
|  |  |  | SD of Analysts | 2.01 | 2.00 | 7.76 | 3.60 | 6.64 | **86.06** | | |  |  |  |  |  |  |
|  |  |  | **RSD of Analysts (%)** | **2.36** | **2.22** | **8.69** | **4.96** | **7.09** |  |  |  |  |  |  |  |  |  |
|  |  | 1000 | Analyst I | 881.60 | 969.21 | 934.57 | 695.30 | 864.36 | 869.01 | 105.71 | **12.16** | 25177.86 | 5659.29 | 6506.19 | 110.30 | 0.1271 |  |
|  |  |  | Analyst II | 723.25 | 916.86 | 845.12 | 815.25 | 1061.34 | 872.37 | 126.43 | **14.49** |  |  |  |  |  |  |
|  |  |  | Analyst III | 862.42 | 878.59 | 953.54 | 678.82 | 940.99 | 862.87 | 110.05 | **12.75** |  |  |  |  |  |  |
|  |  |  | Mean of Analysts | 822.42 | 921.56 | 911.08 | 729.79 | 955.56 | **Mean (general)** | | |  |  |  |  |  |  |
|  |  |  | SD of Analysts | 86.42 | 45.49 | 57.90 | 74.47 | 99.30 | **868.08** | | |  |  |  |  |  |  |
|  |  |  | **RSD of Analysts (%)** | **10.51** | **4.94** | **6.36** | **10.20** | **10.39** |  |  |  |  |  |  |  |  |  |
| 45 | 1,2-dibromo-3-chloropropane | 100 | Analyst I | 86.16 | 93.92 | 75.04 | 80.40 | 94.26 | 85.96 | 8.41 | **9.78** | 116.47 | 25.42 | 30.35 | 7.47 | 0.0877 | **0.098** |
|  |  |  | Analyst II | 86.69 | 88.75 | 83.21 | 75.58 | 86.78 | 84.20 | 5.21 | **6.19** |  |  |  |  |  |  |
|  |  |  | Analyst III | 76.13 | 99.99 | 84.44 | 80.84 | 84.68 | 85.22 | 8.96 | **10.51** |  |  |  |  |  |  |
|  |  |  | Mean of Analysts | 83.00 | 94.22 | 80.89 | 78.94 | 88.57 | **Mean (general)** | | |  |  |  |  |  |  |
|  |  |  | SD of Analysts | 5.95 | 5.63 | 5.11 | 2.91 | 5.04 | **85.12** | | |  |  |  |  |  |  |
|  |  |  | **RSD of Analysts (%)** | **7.17** | **5.97** | **6.32** | **3.69** | **5.69** |  |  |  |  |  |  |  |  |  |
|  |  | 1000 | Analyst I | 892.60 | 931.93 | 953.64 | 731.89 | 847.41 | 871.49 | 87.91 | **10.09** | 11635.17 | 7033.98 | 1533.73 | 92.56 | 0.1074 |  |
|  |  |  | Analyst II | 745.62 | 874.75 | 761.06 | 858.16 | 1040.53 | 856.02 | 117.91 | **13.77** |  |  |  |  |  |  |
|  |  |  | Analyst III | 889.10 | 844.80 | 919.38 | 714.55 | 922.54 | 858.07 | 86.10 | **10.03** |  |  |  |  |  |  |
|  |  |  | Mean of Analysts | 842.44 | 883.83 | 878.03 | 768.20 | 936.83 | **Mean (general)** | | |  |  |  |  |  |  |
|  |  |  | SD of Analysts | 83.87 | 44.27 | 102.73 | 78.39 | 97.35 | **861.86** | | |  |  |  |  |  |  |
|  |  |  | **RSD of Analysts (%)** | **9.96** | **5.01** | **11.70** | **10.20** | **10.39** |  |  |  |  |  |  |  |  |  |

| **S.10.** continuous… | | | | | | | | | | | | | | | | | |
| --- | --- | --- | --- | --- | --- | --- | --- | --- | --- | --- | --- | --- | --- | --- | --- | --- | --- |
| **No** | **VOCs** | ***Spike Conc. µg/L*** | **Analysts** | **Recoveries, µg/L** | | | | | **Intra Days Control** | | | **Sd^2^** | **Sr^2^** | **S_L_^2^** | **S_WR_** | **RSD_WR_** | **RSD_WRpool_** |
|  |  |  |  | **1st Day** | **2nd Day** | **3rd Day** | **4th Day** | **5th Day** | **Mean** | **SD** | **RSD %** |  |  |  |  |  |  |

| 46 | 1,2,4-trichlorobenzene | 100 | Analyst I | 75.16 | 85.61 | 80.88 | 75.52 | 89.79 | 81.39 | 6.36 | **7.82** | 172.07 | 70.65 | 33.81 | 10.22 | 0.1194 | **0.124** |
| --- | --- | --- | --- | --- | --- | --- | --- | --- | --- | --- | --- | --- | --- | --- | --- | --- | --- |
|  |  |  | Analyst II | 91.36 | 74.41 | 88.95 | 69.78 | 89.20 | 82.74 | 9.90 | **11.96** |  |  |  |  |  |  |
|  |  |  | Analyst III | 87.56 | 94.37 | 101.59 | 76.64 | 102.77 | 92.59 | 10.81 | **11.68** |  |  |  |  |  |  |
|  |  |  | Mean of Analysts | 84.69 | 84.80 | 90.47 | 73.98 | 93.92 | **Mean (general)** | | |  |  |  |  |  |  |
|  |  |  | SD of Analysts | 8.47 | 10.00 | 10.44 | 3.68 | 7.67 | **85.57** | | |  |  |  |  |  |  |
|  |  |  | **RSD of Analysts (%)** | **10.00** | **11.80** | **11.54** | **4.97** | **8.17** |  |  |  |  |  |  |  |  |  |
|  |  | 1000 | Analyst I | 917.87 | 1039.33 | 967.59 | 801.78 | 1093.51 | 964.01 | 112.81 | **11.70** | 30413.35 | 4823.43 | 8529.97 | 115.56 | 0.1277 |  |
|  |  |  | Analyst II | 933.73 | 899.51 | 796.20 | 749.71 | 947.01 | 865.23 | 87.56 | **10.12** |  |  |  |  |  |  |
|  |  |  | Analyst III | 821.08 | 988.93 | 908.69 | 705.75 | 1000.90 | 885.07 | 123.51 | **13.95** |  |  |  |  |  |  |
|  |  |  | Mean of Analysts | 890.89 | 975.92 | 890.83 | 752.41 | 1013.81 | **Mean (general)** | | |  |  |  |  |  |  |
|  |  |  | SD of Analysts | 60.98 | 70.81 | 87.08 | 48.07 | 74.10 | **904.77** | | |  |  |  |  |  |  |
|  |  |  | **RSD of Analysts (%)** | **6.84** | **7.26** | **9.78** | **6.39** | **7.31** |  |  |  |  |  |  |  |  |  |
| 47 | Hexachloro-1,3-buthadiene | 100 | Analyst I | 85.80 | 98.55 | 88.81 | 71.31 | 97.55 | 88.41 | 11.02 | **12.46** | 320.07 | 42.64 | 92.48 | 11.62 | 0.1331 | **0.135** |
|  |  |  | Analyst II | 86.27 | 89.24 | 78.99 | 71.55 | 101.47 | 85.50 | 11.25 | **13.16** |  |  |  |  |  |  |
|  |  |  | Analyst III | 73.98 | 108.26 | 86.66 | 79.94 | 91.87 | 88.14 | 13.12 | **14.89** |  |  |  |  |  |  |
|  |  |  | Mean of Analysts | 82.02 | 98.68 | 84.82 | 74.27 | 96.96 | **Mean (general)** | | |  |  |  |  |  |  |
|  |  |  | SD of Analysts | 6.97 | 9.51 | 5.16 | 4.92 | 4.83 | **87.35** | | |  |  |  |  |  |  |
|  |  |  | **RSD of Analysts (%)** | **8.50** | **9.64** | **6.09** | **6.62** | **4.98** |  |  |  |  |  |  |  |  |  |
|  |  | 1000 | Analyst I | 788.22 | 1039.33 | 967.59 | 801.78 | 1093.51 | 938.09 | 138.12 | **14.72** | 30573.83 | 6948.50 | 7875.11 | 121.75 | 0.1376 |  |
|  |  |  | Analyst II | 933.73 | 828.20 | 796.20 | 749.71 | 947.01 | 850.97 | 86.38 | **10.15** |  |  |  |  |  |  |
|  |  |  | Analyst III | 821.08 | 988.93 | 812.27 | 705.75 | 1000.90 | 865.79 | 126.39 | **14.60** |  |  |  |  |  |  |
|  |  |  | Mean of Analysts | 847.68 | 952.15 | 858.69 | 752.41 | 1013.81 | **Mean (general)** | | |  |  |  |  |  |  |
|  |  |  | SD of Analysts | 76.31 | 110.26 | 94.66 | 48.07 | 74.10 | **884.95** | | |  |  |  |  |  |  |
|  |  |  | **RSD of Analysts (%)** | **9.00** | **11.58** | **11.02** | **6.39** | **7.31** |  |  |  |  |  |  |  |  |  |
| 48 | 1,2,3-trichlorobenzene | 100 | Analyst I | 79.16 | 103.56 | 77.78 | 100.19 | 87.64 | 89.66 | 11.83 | **13.19** | 58.59 | 68.23 | -3.21 | 8.06 | 0.0931 | **0.093** |
|  |  |  | Analyst II | 89.57 | 77.58 | 81.55 | 78.33 | 93.83 | 84.17 | 7.19 | **8.55** |  |  |  |  |  |  |
|  |  |  | Analyst III | 82.88 | 91.16 | 82.33 | 83.93 | 90.32 | 86.12 | 4.26 | **4.95** |  |  |  |  |  |  |
|  |  |  | Mean of Analysts | 83.87 | 90.76 | 80.55 | 87.48 | 90.60 | **Mean (general)** | | |  |  |  |  |  |  |
|  |  |  | SD of Analysts | 5.27 | 12.99 | 2.43 | 11.35 | 3.10 | **86.65** | | |  |  |  |  |  |  |
|  |  |  | **RSD of Analysts (%)** | **6.29** | **14.32** | **3.02** | **12.98** | **3.43** |  |  |  |  |  |  |  |  |  |
|  |  | 1000 | Analyst I | 918.87 | 1007.98 | 915.88 | 813.47 | 881.64 | 907.57 | 70.37 | **7.75** | 13081.85 | 3970.47 | 3037.13 | 83.71 | 0.0931 |  |
|  |  |  | Analyst II | 821.98 | 955.62 | 828.22 | 774.49 | 1082.57 | 892.58 | 125.64 | **14.08** |  |  |  |  |  |  |
|  |  |  | Analyst III | 836.55 | 913.74 | 900.49 | 872.68 | 959.81 | 896.65 | 46.05 | **5.14** |  |  |  |  |  |  |
|  |  |  | Mean of Analysts | 859.13 | 959.11 | 881.53 | 820.21 | 974.68 | **Mean (general)** | | |  |  |  |  |  |  |
|  |  |  | SD of Analysts | 52.24 | 47.22 | 46.80 | 49.44 | 101.28 | **898.93** | | |  |  |  |  |  |  |
|  |  |  | **RSD of Analysts (%)** | **6.08** | **4.92** | **5.31** | **6.03** | **10.39** |  |  |  |  |  |  |  |  |  |

| **S.11.** The values of VOCs in *CRM* (*X_CRM_*), recovery values of VOCs (*X_Exp_*), seven replicate standard deviation values (*S_Exp_*), *t* values, and *Z*−score. | | | | | | | | | |
| --- | --- | --- | --- | --- | --- | --- | --- | --- | --- |
| **No** | **VOCs** | **X_CRM_** | **X_Exp_** | **S _Exp_** | | ***t*** | ***t_critic_***^a^ | ***Z* −score** | |
| 1 | Trichlorofluoromethane | 397 | 425.2 | 35.0 | | 2.12 | 2.45 | 0.52 | |
| 2 | 1.1-dichloroethene | 382 | 406.3 | 27.6 | | 2.30 |  | 0.34 | |
| 3 | *Trans*-1,2-dichloroethene | 305 | 328.0 | 34.7 | | 1.75 |  | 0.39 | |
| 4 | 1,2-dichloroethane | 100 | 105.1 | 5.7 | | 2.32 |  | 0.22 | |
| 5 | *Cis*-1,2-dichloroethene | 531 | 495.1 | 39.1 | | 2.44 |  | -0.39 | |
| 6 | Bromochloromethane | 219 | 239.2 | 21.9 | | 2.42 |  | 0.45 | |
| 7 | Trichlromethane | 120 | 118.4 | 6.7 | | 0.79 |  | -0.08 | |
| 8 | 1,1,1-trichloroethane | 804 | 850.0 | 78.0 | | 1.56 |  | 0.35 | |
| 9 | Benzene | 825 | 906.3 | 88.7 | | 2.42 |  | 0.60 | |
| 10 | Tetrachloro methane | 429 | 467.1 | 41.6 | | 2.42 |  | 0.49 | |
| 11 | Trichloro ethene | 606 | 670.1 | 75.9 | | 2.23 |  | 0.61 | |
| 12 | 1.2-dichloro propane | 100 | 95.5 | 6.4 | | 2.07 |  | -0.22 | |
| 13 | Dibromo methane | 50 | 46.3 | 4.9 | | 2.16 |  | -0.32 | |
| 14 | Bromodichloromethane | 219 | 240.9 | 23.8 | | 2.33 |  | 0.48 | |
| 15 | 1.3-dichloro propene (*cis+trans)* | 390 | 419.1 | 35.9 | | 2.14 |  | 0.40 | |
| 16 | Toluen | 70 | 68.0 | 5.7 | | 0.93 |  | -0.12 | |
| 17 | 1.1.2-trichloro ethane | 250 | 247.3 | 10.8 | | 0.73 |  | -0.06 | |
| 18 | Tetrachloro ethene | 100 | 97.1 | 11.8 | | 0.67 |  | -0.13 | |
| 19 | 1.3-dichloro propane | 353 | 379.0 | 28.6 | | 2.41 |  | 0.39 | |
| 20 | Dibromochloromethane | 484 | 457.3 | 36.7 | | 1.95 |  | -0.31 | |
| 21 | 1.2-dibromoethane | 50 | 47.5 | 5.9 | | 1.35 |  | -0.24 | |
| 22 | Chlorobenzene | 743 | 792.1 | 56.4 | | 2.30 |  | 0.39 | |
| 23 | 1.1.1.2-tetrachloroethane | 50 | 47.2 | 3.5 | | 2.27 |  | -0.24 | |
| 24 | Ethylbenzene | 315 | 347.7 | 45.6 | | 1.86 |  | 0.53 | |
| 25 | *m.p*-xylene+*o*-xylene (total) | 1600 | 1450.6 | 164.3 | | 2.42 |  | -0.63 | |
| 26 | Styren | 626 | 673.3 | 61.5 | | 2.02 |  | 0.44 | |
| 27 | 1.1.2.2-tetrachloroethane | 100 | 90.2 | 10.9 | | 2.43 |  | -0.44 | |
| 28 | 1.3-dichlorobenzene | 520 | 568.3 | 55.8 | | 2.28 |  | 0.52 | |
| 29 | 1.4-dichlorobenzene | 80 | 78.1 | 6.6 | | 0.80 |  | -0.11 | |
| 30 | 1.2-dichlorobenzene | 290 | 319.0 | 34.6 | | 2.22 |  | 0.52 | |
| 31 | 1.2.4-trichlorobenzene | 150 | 147.4 | 6.8 | | 1.17 |  | -0.09 | |
| 32 | Hexachloro-1.3-buthadiene | 603 | 654.3 | 61.4 | | 2.20 |  | 0.49 | |
| ^a:^ p=0.05 and degrees of freedom,6 | | | | |  |  |  |  |  |


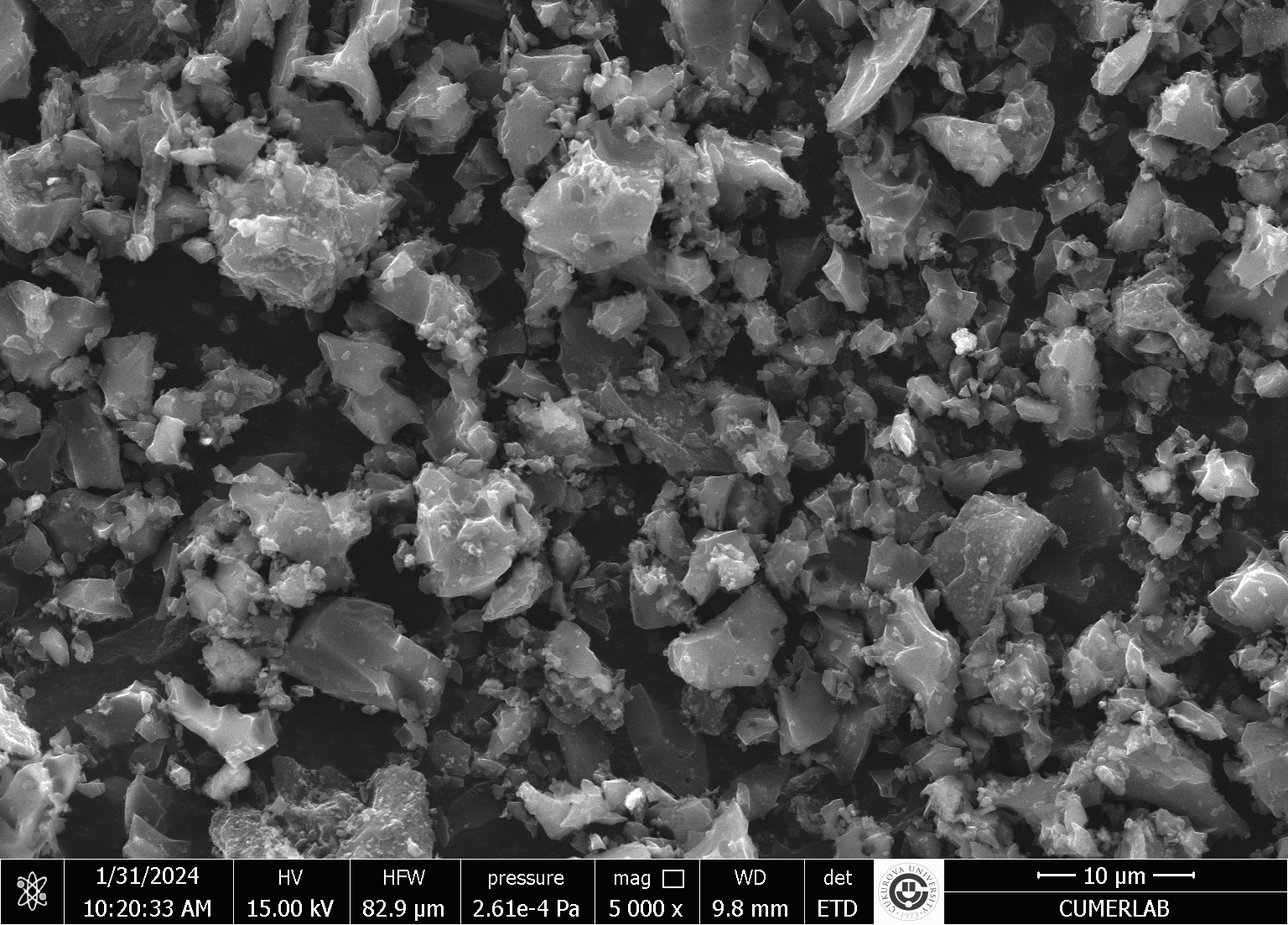


**S.12.** SEM images of the AC at 5,000X magnification (A)


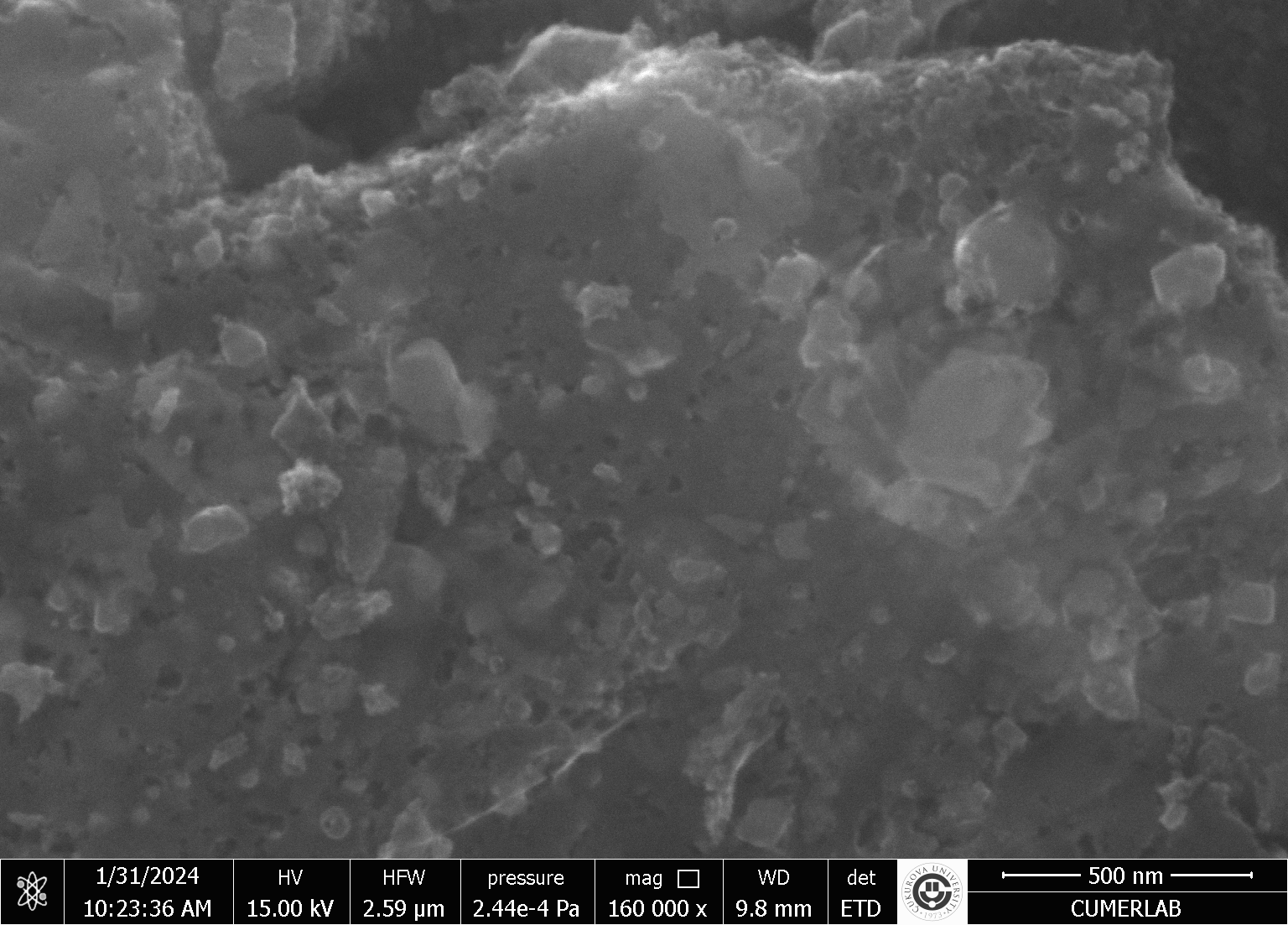


**S.13.** SEM images of the AC at 160,000X magnification (B)


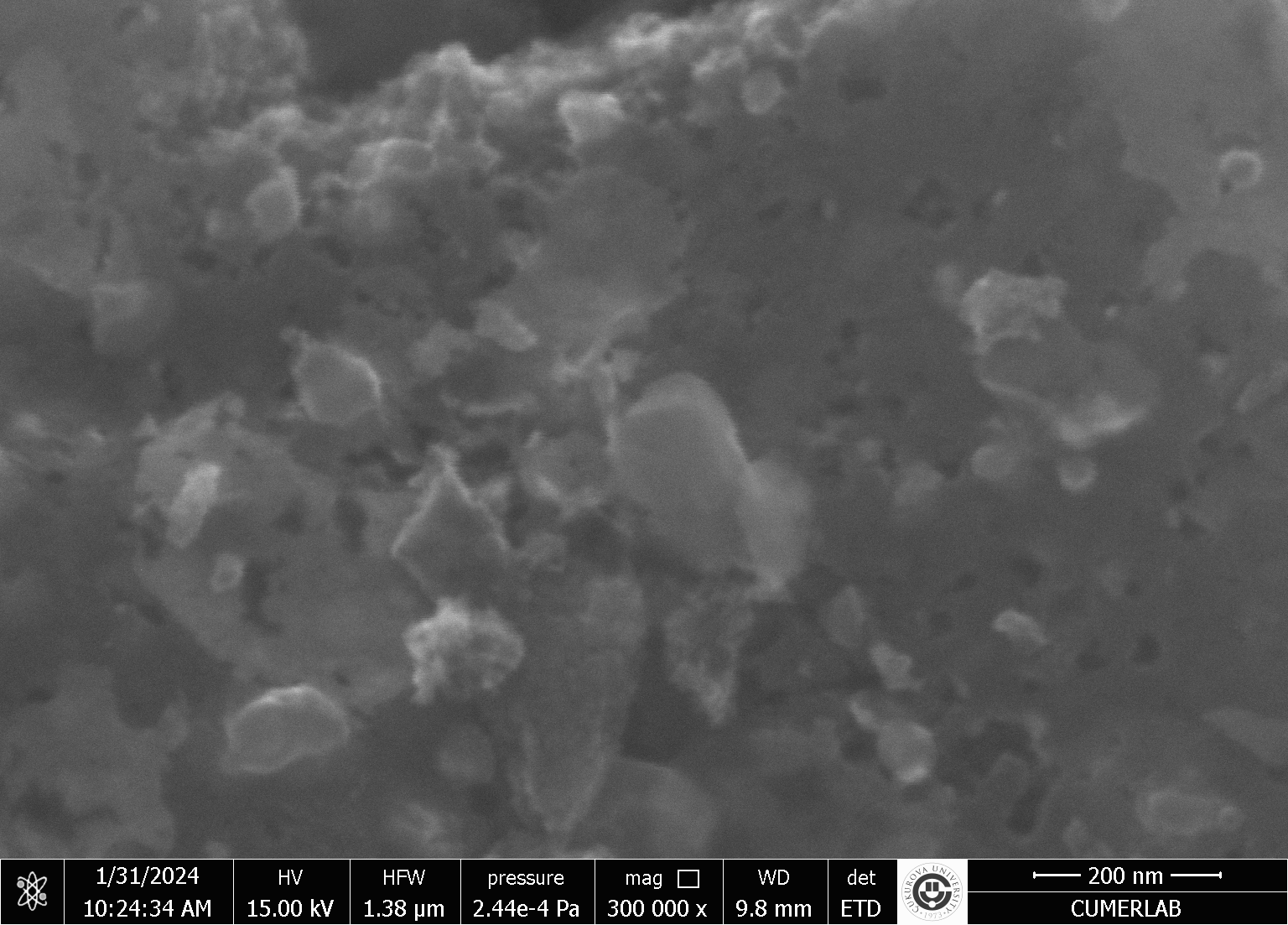


**S.14.** SEM images of the AC at 300,000X magnification (C)


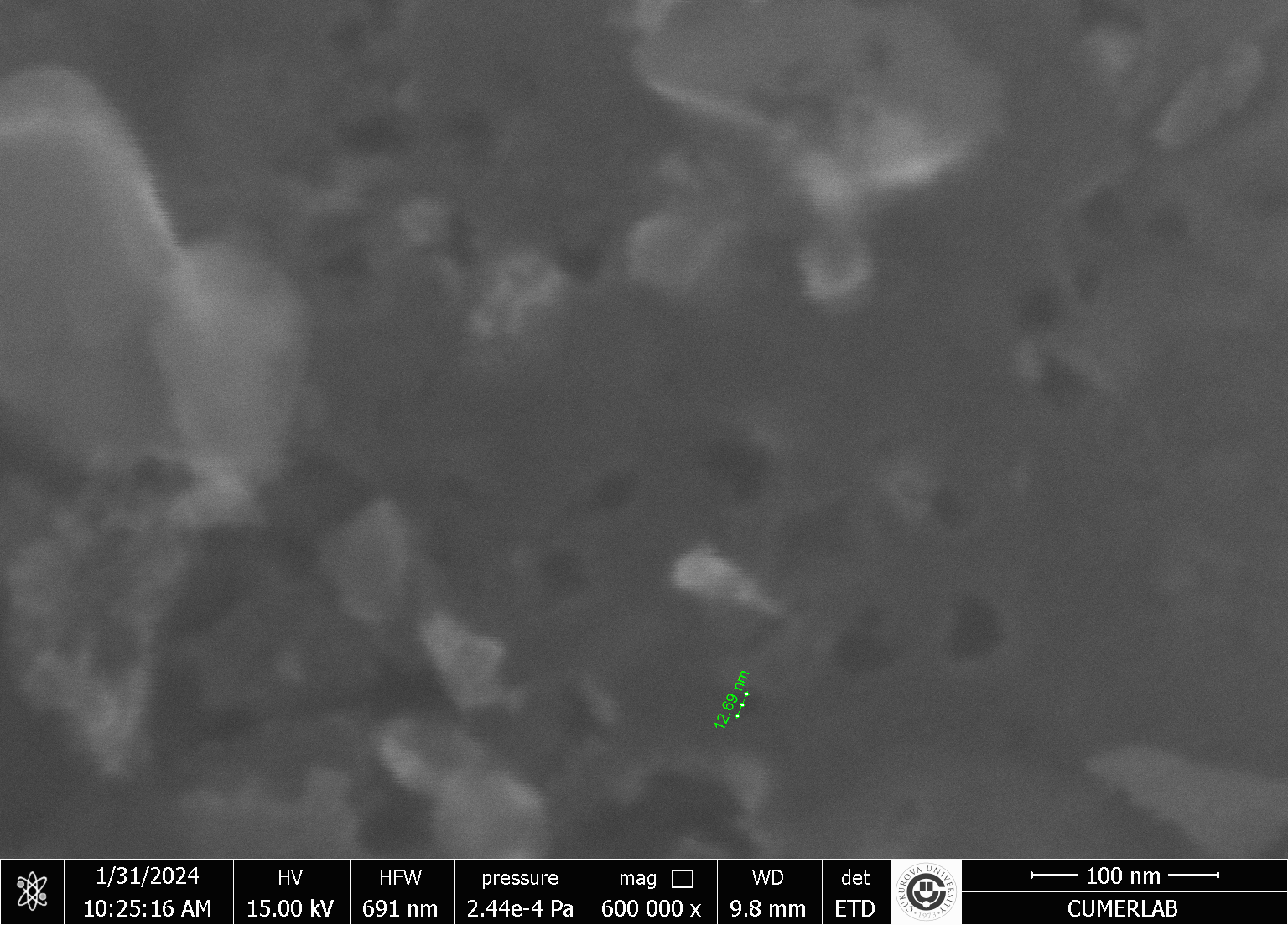


**S15.** SEM images of the AC at 600,000X magnification (D)


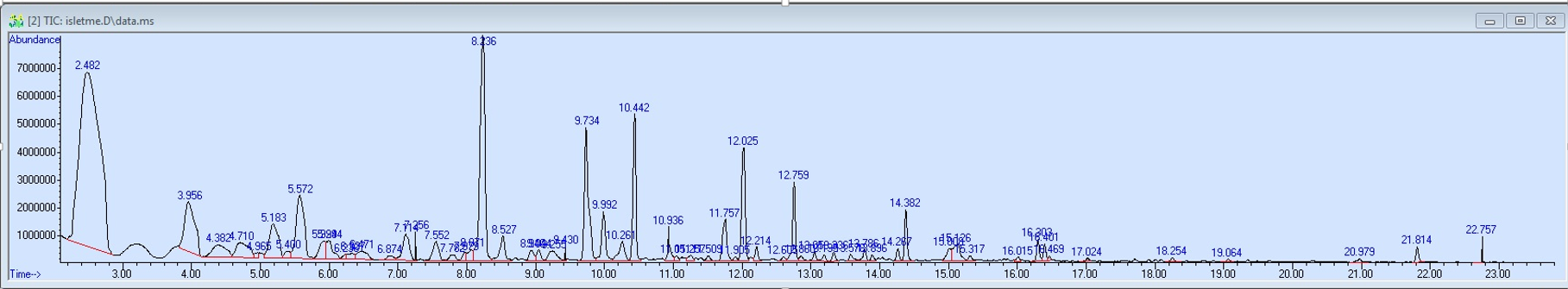


**S16.** Total Ion Chromatogram of the distillate from hazelnut shell at 500 °C

| **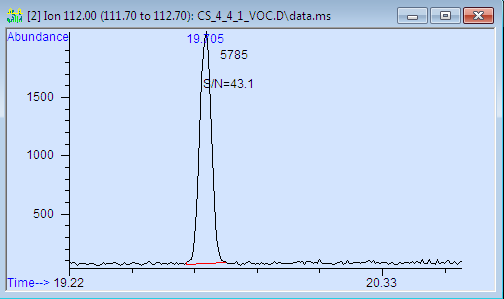** |  | **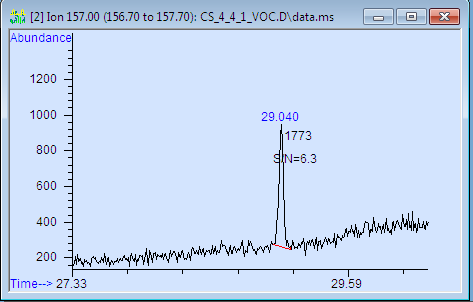** |
| --- | --- | --- |
| **S17.** The chromatograms of *Chlorobenzene* with the highest S / N ratio and 1,*2-dibromo-3-chloro propane* with the lowest S / N ratio at 100 µg/dm^3^ concentration | | |


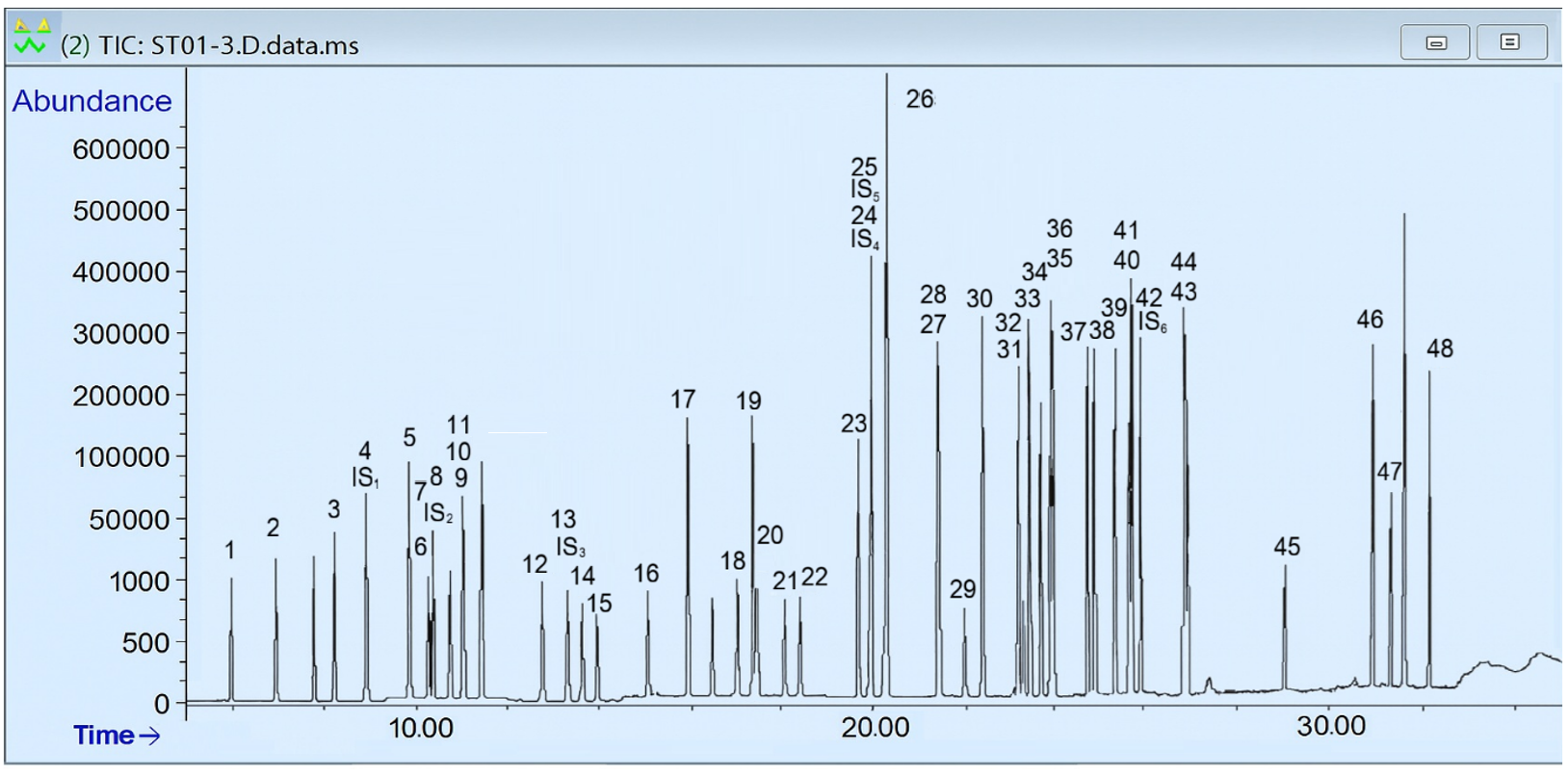


**S18.** The total ion chromatogram of 48 VOCs
