# Supplementary material for: Using activated carbon produced from hazelnut shells as an adsorbent for the quantitative analysis of volatile organic compounds by GC-MS
Source: Anal Bioanal Chem. 2026 Feb 18;418(9):2669–84. doi: 10.1007/s00216-026-06394-5 (PMC13079479; doi:10.1007/s00216-026-06394-5)
Supplement: Supplementary file 2 — Supplementary file2 (DOCX 20.5 MB) [file 216_2026_6394_MOESM2_ESM.docx]

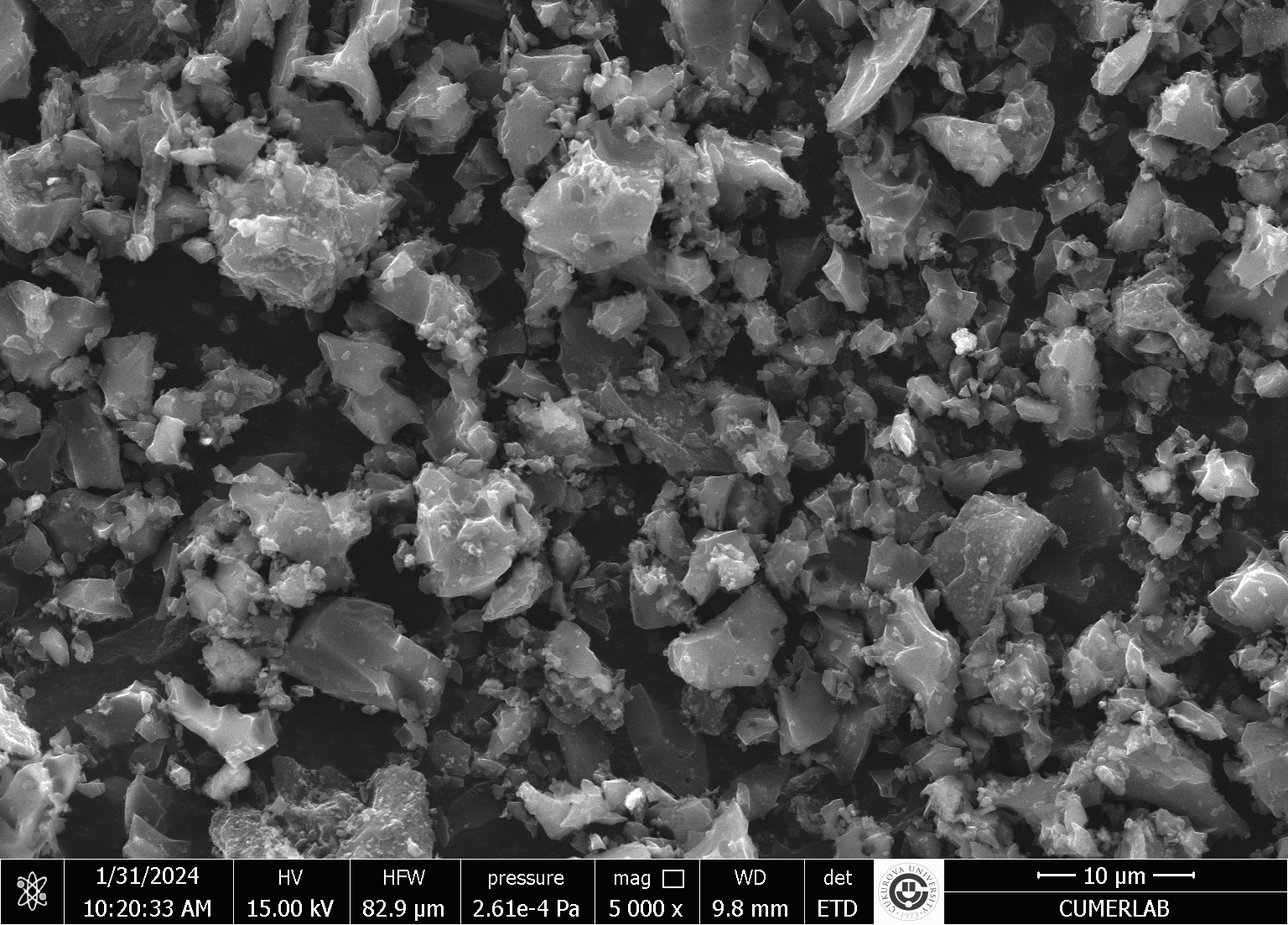


**S.12.** SEM images of the AC at 5,000X magnification (A)


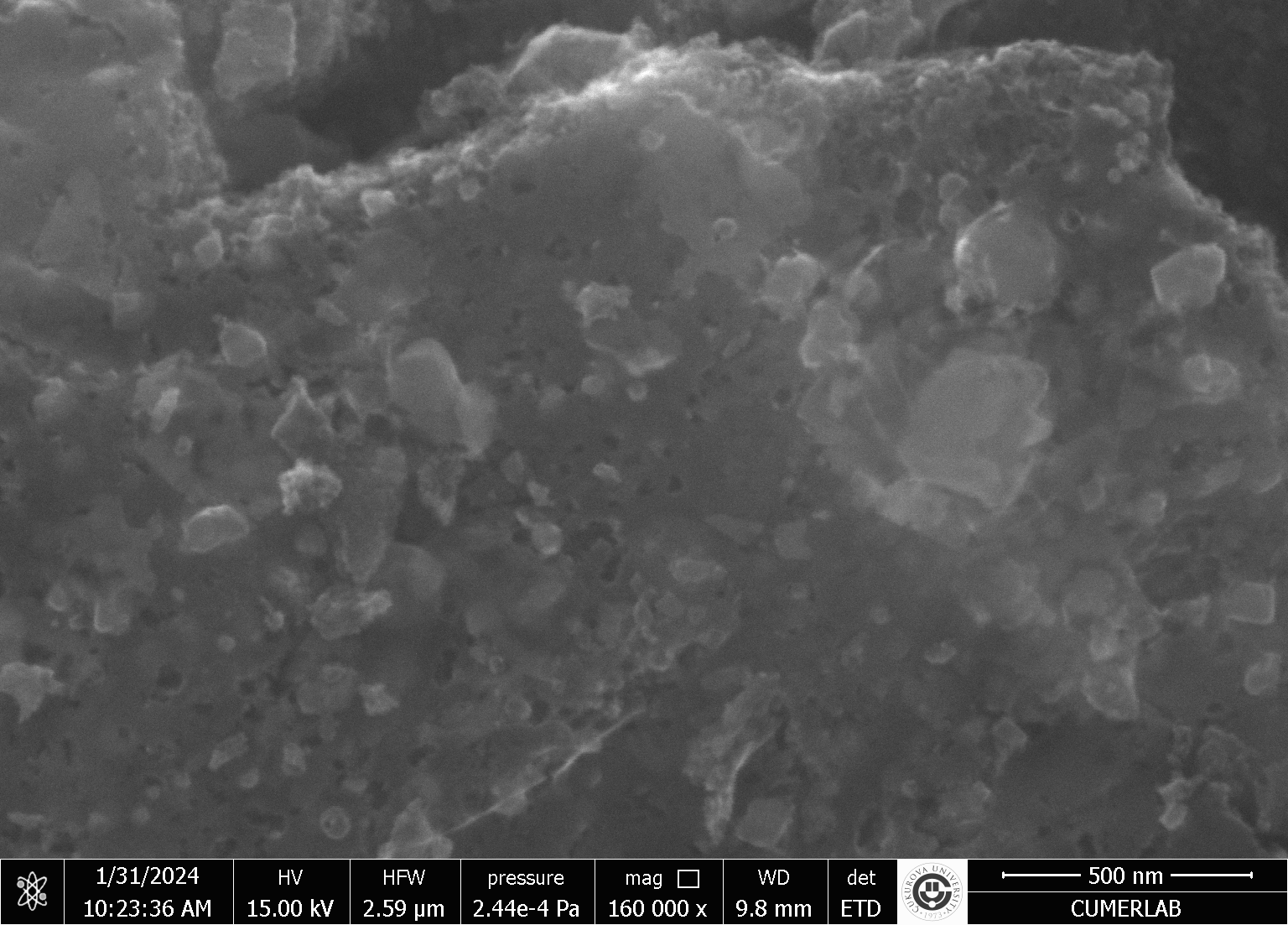


**S.13.** SEM images of the AC at 160,000X magnification (B)


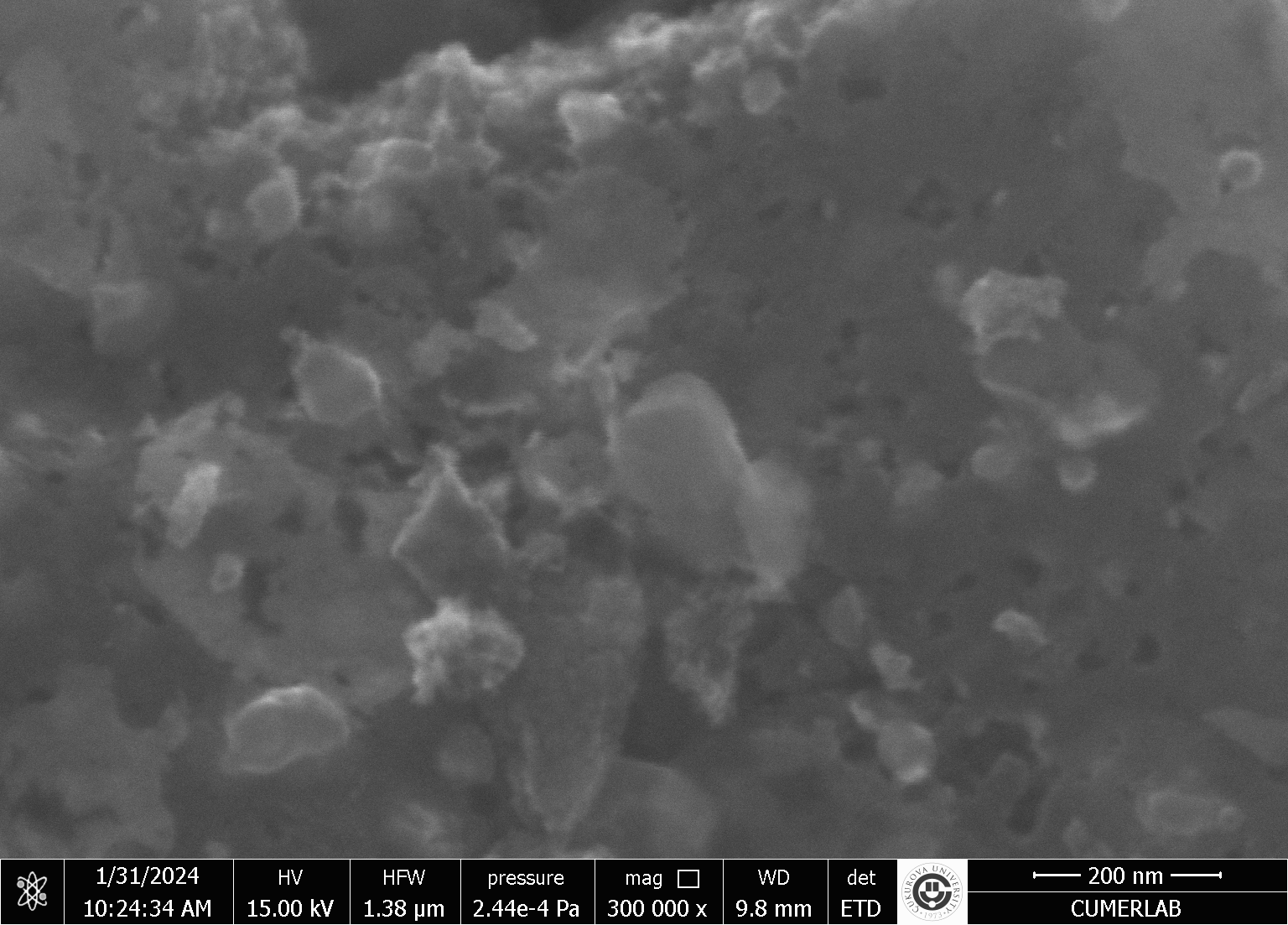


**S.14.** SEM images of the AC at 300,000X magnification (C)


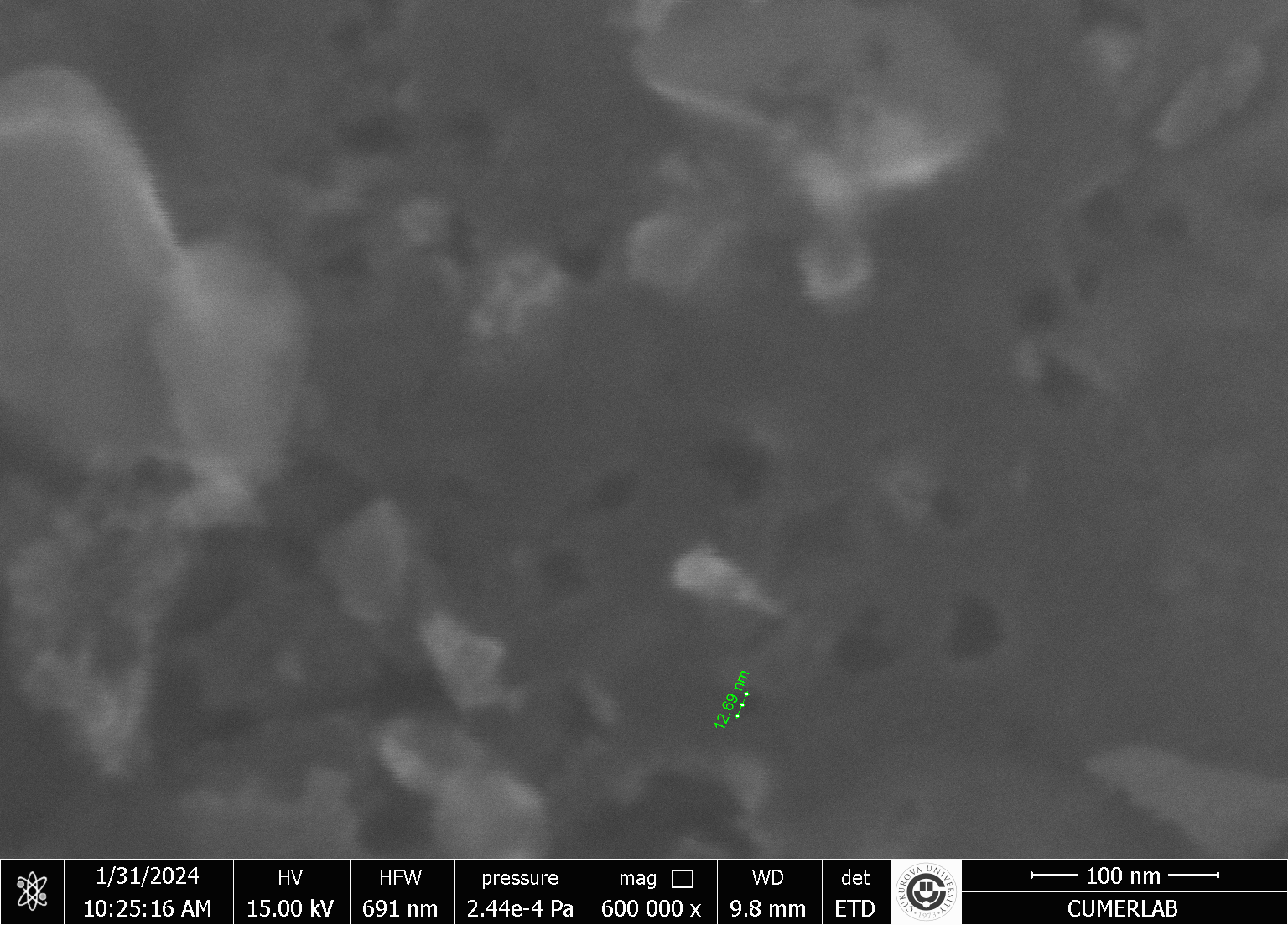


**S15.** SEM images of the AC at 600,000X magnification (D)


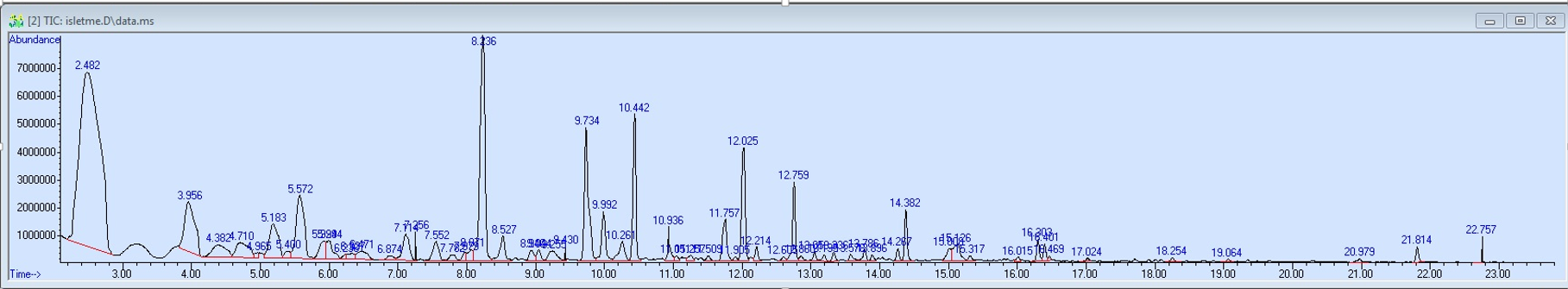


**S16.** Total Ion Chromatogram of the distillate from hazelnut shell at 500 °C

| **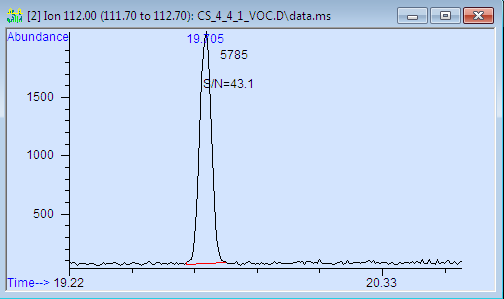** |  | **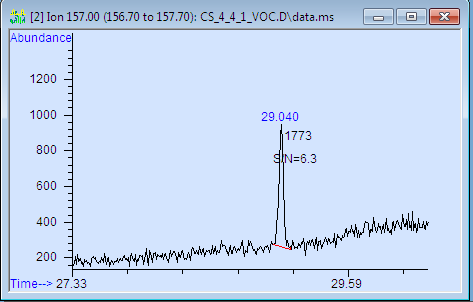** |
| --- | --- | --- |
| **S17.** The chromatograms of *Chlorobenzene* with the highest S / N ratio and 1,*2-dibromo-3-chloro propane* with the lowest S / N ratio at 100 µg/dm^3^ concentration | | |


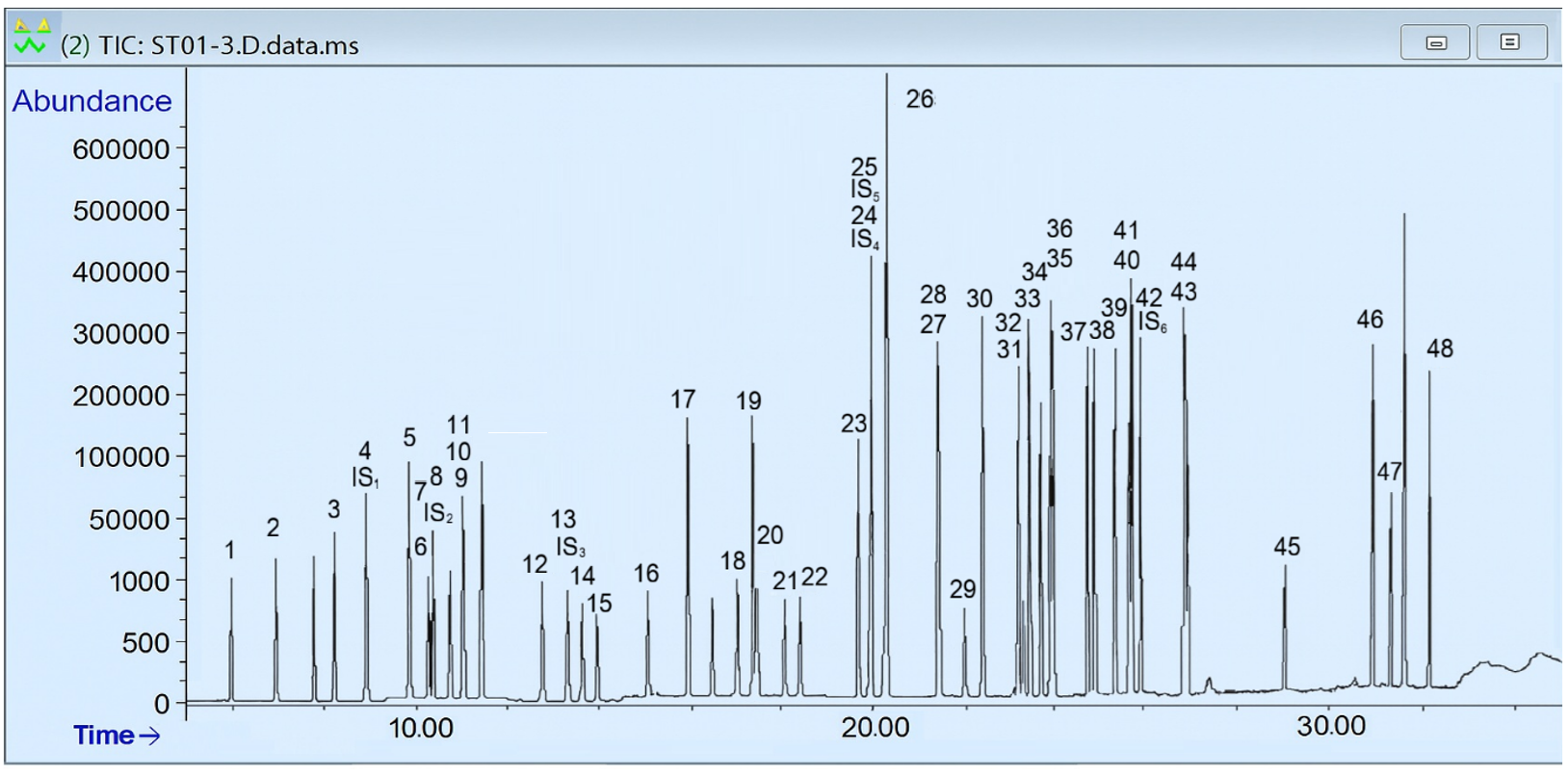


**S18.** The total ion chromatogram of 48 VOCs
